# Conductivity Spectroscopy for Investigation and Discovery of Photovoltaic Materials

**DOI:** 10.1021/acs.chemrev.5c00986

**Published:** 2026-05-19

**Authors:** Obadiah G. Reid, Bryon W. Larson

**Affiliations:** † Chemistry and Nanoscience Center, National Laboratory of the Rockies, Golden, Colorado 80401, United States; ‡ Renewable and Sustainable Energy Institute, 1877 University of Colorado Boulder, Boulder, Colorado 80309, United States

## Abstract

Conductivity spectroscopy is an extremely powerful set of methods for probing the properties of optoelectronic materials, especially photovoltaics, where photoconductivity is one of the best spectroscopic proxies for performance. Despite this power, they are substantially less commonly used than time-resolved photoluminescence (for instance) because they tend to be more expensive to implement (THz) and/or require specialized knowledge (GHz) to construct instruments, which are not widely available. The goal of this review is to illustrate the utility of these experiments in the discovery and study of photovoltaic absorber materials and simultaneously make them more accessible to the community by providing a central tutorial resource. We provide a comprehensive review of how conductivity spectroscopy has developed over the past decade and been applied in the discovery and development of photovoltaic materials, with a primary focus on emerging solution-processable technologies. Along the way we aim to demystify conductivity spectroscopy with focused tutorial sections that explain the physical models used to fit the data and illustrate how to think about “high-frequency conductivity”.

## Graphical abstract

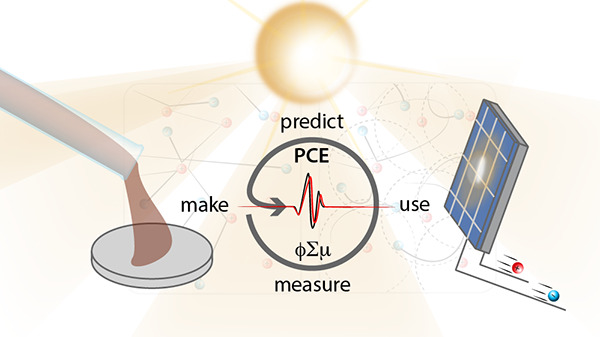

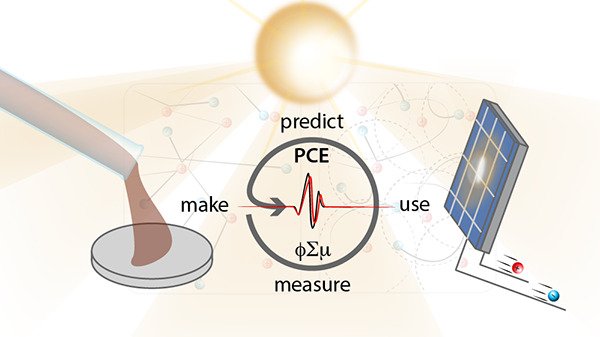

## 1 Introduction

Methylammonium lead iodide (MAPbI_3_) has been known since at least 1978,^1^ when Dieter Weber reported its structure and the fact that it did not self-dope like its earlier Sn-based analogs. This discovery took place in Stuttgart, only 570 km from Delft, where John Warman and colleagues were in the process of inventing what we now call time-resolved microwave conductivity (TRMC).^2,3^ Although the group at Delft did not apply their apparatus to study solid semiconductors for several years,^4^ the capability was there, and it had been known since at least the 1960s^5,6^ that this was a viable way of characterizing the photoconductivity of semiconductors; Kunst and Beck had already begun using it for quantitative measurements on silicon by the mid 80s.^7^

It is interesting to contemplate how different the world might be today, had John Warman and Dieter Weber been drinking buddies. The phenomenal semiconducting properties of MAPbI_3_ could have been revealed in 1980! Instead, it took 36 years from Weber’s report until photoconductivity spectroscopy finally unveiled MAPbI_3_s characteristics,^8,9^ only after it had been slowly revealed as a good photovoltaic material. This general problem of “imperfect knowledge” is a historic and ongoing tragedy for science and engineering: many discoveries and technologies could emerge much sooner if only the people making functional materials and the people with access to unique and powerful measurement tools could find each other more efficiently.

As such, we aim for this to be a pedagogical review article, written primarily for readers who may be unfamiliar with conductivity spectroscopy and what it can do. In particular, anyone with an interest in photovoltaic materials who might want to pick up AC photoconductivity methods as a tool in their research. Our aim is to teach the topic while retaining a comprehensive coverage of the main research area: advances in AC conductivity studies of emerging photovoltaic materials over the past decade.

This review is broken into five main sections. (1) We begin with an extended introductory tutorial that describes conductivity spectroscopy from the ground up including the statistical mechanical connection between diffusion and conductivity. (2) We discuss the standard models that have been adopted for interpreting complex conductivity spectra and make recommendations to new practitioners about those that are particularly insightful. We introduce kinetic modeling as the indispensable tool for understanding transient data, and explain the reason that transient photoconductivity kinetics will nearly always differ from transient photoluminescence. (3) We outline the basic design of common instruments and review recent developments in AC conductivity methods to gain fundamental physical insight into emerging photovoltaic materials. (4) We describe the models that have been developed to extract crucial material properties including implied open-circuit voltage (quasi-Fermi level splitting) and diffusion length, and relate the use of yield-mobility product as a figure of merit in and of itself for organic photovoltaics. (5) Finally, we discuss progress using AC conductivity methods in PV materials discovery, particularly in the emerging area of automated high-throughput laboratories, and demonstrate importance of combining photoconductivity with photoluminescence for this purpose. We employ a simple kinetic model to show why photoconductivity methods are particularly suitable for photovoltaic material discovery in the context of automated high-throughput laboratories.

Several topics are explicitly excluded from the scope of this review, except where they serve a particular illustrative purpose. These include microscopy, studies of semiconductors and nanomaterials not directly relevant to photovoltaic or photoelectrochemical applications, and low-frequency techniques such as impedance and electrochemical impedance spectroscopy.

## 2 An Elementary Introduction to Conductivity Spectroscopy

### 2.1 Complex Conductivity from the Classical Equations of Motion

The word “conductivity” usually conjures the image of metal wires connected to a sample, or some such device structure, and few are unfamiliar with equation

1 $$J=\sigma E=\frac{qN\mu V}{L}=\frac{V}{RA}$$

which gives the current density (*J*) through a material as a function of its conductivity (σ) and the applied electric field (*E*) or, equivalently, the electron charge (*q*), carrier density (*N*), mobility (μ), voltage (*V*), and sample thickness (*L*). Note that this is simply a more detailed expression of Ohm’s law (*V* = *IR*, in which *I* is the current and *R* is the electrical resistance) renormalized to the area (*A*) through which current passes (*J* = *I*/*A*, σ = *L*/*RA*, *E* = *V*/*L*). This equation describes the direct current (DC) limit, where the time variance of the electric field is negligible relative to the response of all elements of the electrical circuit or, equivalently, the materials therein. There is no ambiguity about the origin of DC electric currents: there is net charge motion through a material, either from ionic or electronic drift. However, as soon as the time dependence of the field begins to approach the response time of the circuit (or material), both the current and conductivity become time-dependent as well and must be expressed as complex quantities (e.g., σ̂ = σ′ – iσ″) in the frequency domain. We refer to this as an “AC” measurement.

As we will see, measuring AC conductivity comes with an enhancement in the information content and flexibility of the measurements, but some certainty about the origin of material and/or circuit response is lost because the nuclear motions of polar media can now contribute, as can bound electric charges that could not contribute to a net electric current at infinite time.

The loss in certainty on the physical origin of a measured AC conductivity is compensated by several new capabilities. AC measurements can be time-resolved; they can probe samples without electrical contacts, which need not even be monolithic solids (powders, liquid suspensions, etc.). Finally, frequency-resolved studies can pick apart the conductivity response and assign specific mechanisms to each part of the signal. These enhancements over DC methods are central to the value of AC conductivity measurements for accelerated discovery of semiconductors for photovoltaics.

The interpretation of the imaginary part of the conductivity is particularly difficult for those new to AC conductivity measurements, and it is worth dwelling on. The explanation for why conductivity becomes complex (has both real and imaginary parts) can be approached from the bottom up via the microscopic or molecular nature of the charges that are responding to the applied field, or top down from a circuit theory perspective. The circuit theory point of view holds that the imaginary part of the conductivity contributes current that is π/2 radians out of phase with the applied field, while the real part contributes in-phase current. Thus, complex numbers are needed to describe the full response waveform in polar coordinates and how it evolves with driving frequency. While true, this description is simply a definition; it tells how we mathematically categorize the electrical response of any arbitrary circuit. In the context of AC conductivity spectroscopy adopting this mathematical definition as a conceptual point of view obfuscates the insight into material properties that these quantities allow. Moreover, it is a major goal of AC conductivity measurements to eliminate purely circuit level effects from the instrument and isolate the response of the sample of interest. As such, we shall take the molecular or at least microscopic point of view throughout the remainder of this review, which we develop below to build your physical intuition for what an imaginary conductivity is.

The microscopic point of view starts with the study of a free electron. We take an entirely classical approach to this problem because the interpretation of room-temperature conductivity spectroscopy measurements rarely requires a quantum treatment. Where quantum effects are likely to intrude, we make specific note of this. We can write down the classical equation of motion as follows:

2 $$m\frac{d^{2}x}{dt^{2}}=-qE_{0}\cos\left(\omega t\right)$$

where we have formulated the problem in terms of force. The variables are the mass of the electron (*m*) the position of the electron (*x*), the electron charge (*q*), the applied electric field amplitude (*E*_0_), the radian frequency of that field (ω), and time (*t*). This is just Newton’s second law (*F* = *ma*) where the force applied is the field × charge on the right-hand side and the second derivative of position with respect to time is the acceleration. This equation, along with all subsequent development of AC conductivity in this section is available as a Python module in the Supporting Information.

Solving this equation for the velocity of the electron (d*x*/d*t*) leads to the waveforms shown in Figure 1b, where the black trace shows the applied electric field, and the blue trace the electron velocity. Velocity and current density are related by a constant (*Nq*). Thus, it is immediately clear that the response of the free electron to an AC electric field is an entirely imaginary conductivity by the definition given above, as the current (velocity) is exactly π/2 radians out of phase with the applied field. Why? Imagine an electron that starts classically “at rest”. The electric field (*E* = *E*_0_ cos­(ω*t*)) starts out pointing in one direction, and diminishes until ω*t* = π/2. This corresponds to the maximum velocity of the electron in that direction. Thereafter the field grows in the opposite direction, applying the opposite sign of acceleration to the electron, which comes back to “rest” at ω*t* = π. By the time ω*t* = 2π the electron will have both come back to its original position in space (*x* = 0) and be at rest (d*x*/d*t* = *v* = 0).

**Figure 1:**
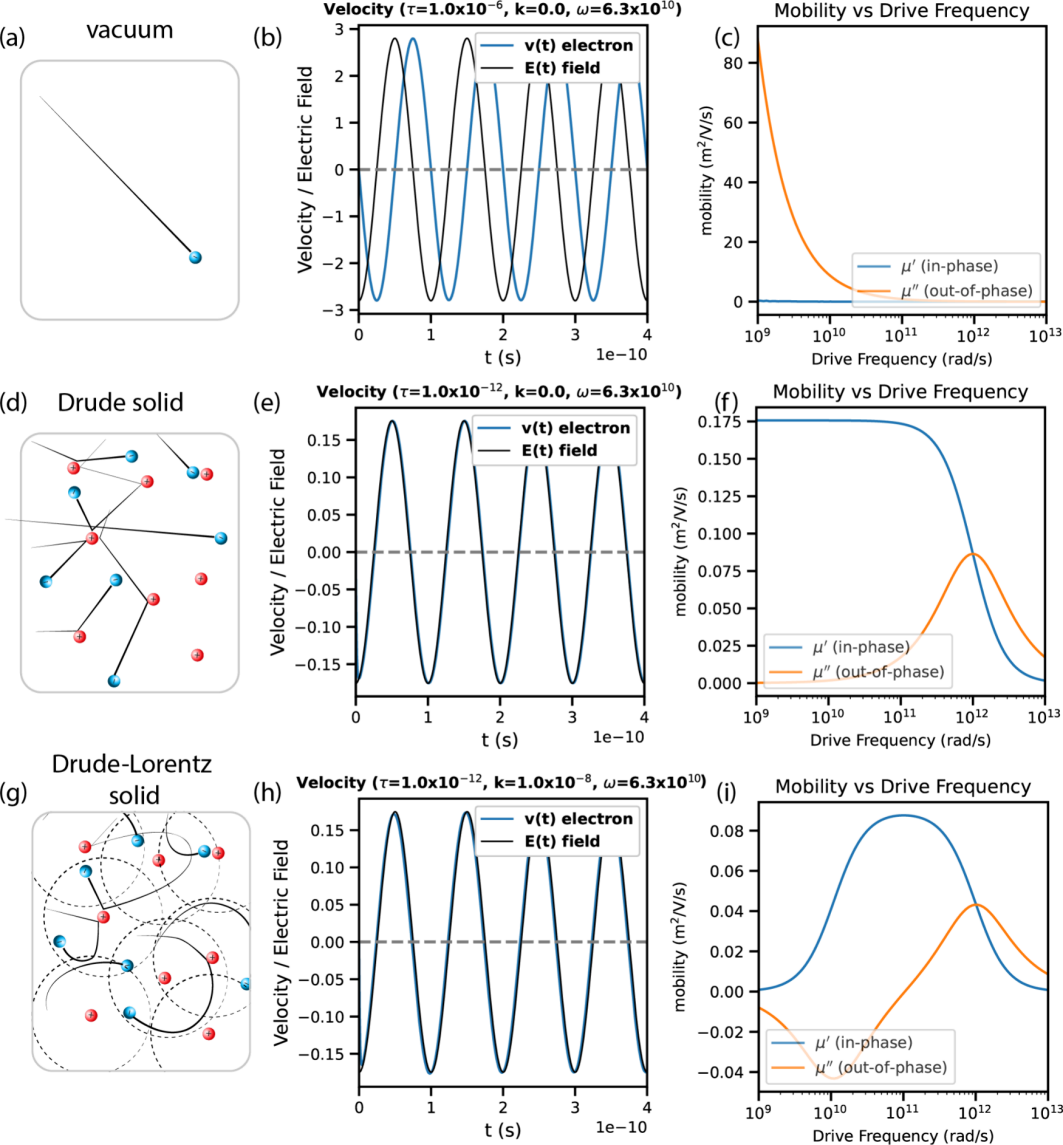
Examples of the classical motion of an electron driven by an AC electric field. (a) Cartoon of an electron traversing a vacuum. (b) Corresponding solution to eq 2 showing an electric current that is π/2 radians out of phase with the applied field. (c) Resulting complex mobility as a function of drive frequency when the equation is solved for many applied fields and a discrete Fourier transform is applied to extract the real and imaginary parts of the current. (d) Cartoon of a Drude-like solid, where electrons are subject to velocity-randomizing collisions with elements of the solid—ionized impurities in this example. (e) Corresponding solution to eq 3 when the average time between scattering events (τ) is set to 1 ps at a drive frequency where the resulting current (velocity) is mostly in-phase (*f* = 10 GHz). (f) Complex mobility as a function of drive frequency that results. (g) Cartoon of a Drude–Lorentz solid wherein the electrons are subject to both velocity-randomizing collisions and a harmonic restoring force (eq 5), illustrated in this case as a Coulomb attraction to the ionized impurities. (h) Corresponding current (velocity) of an electron in this system, also driven at *f* = 10 GHz. The response remains mostly in-phase but is slightly phase-shifted relative to that shown in (e). (i) Resulting complex mobility for this Drude–Lorentz model, where the mobility approaches 0 as the frequency approaches zero, as all charges are bound by a harmonic restoring force.

This is an important physical insight: imaginary conductivity arises from systems that do not dissipate energy, even though clearly an electron in a vacuum would have real measurable electrical conductivity at zero frequency—indeed, at zero frequency the resistance is zero, and the conductivity is infinite in the absence of space-charge effects.^10^

Why is no energy dissipated? Surely one cannot move an object without doing work! In fact, the electron has not moved on average. Another way of saying this is that by accelerating the electron in these oscillations it emits exactly as much electromagnetic energy as it absorbs, but with a phase shift that corresponds to its velocity waveform in Figure 1b. This radiated power (*P*) for any accelerated charged particle can be calculated from the Larmor formula (*P* = μ_0_ *q* ^2^ *a* ^2^/6π*c*), where *c* is the speed of light, μ_0_ is the permeability of free space, *q* is the electron charge, and *a* is the acceleration. As a result of all this, no energy is absorbed from the probing electric field, and the presence of the electron is sensed by the phase-shift it induces in the radiation. In contrast, real contributions to the conductivity result in power absorption.

In Figure 1c we can take this analysis one step further. We solve the equation of motion for many applied field frequencies, and use a wave mixing analysis to extract the real and imaginary parts of the electronic mobility using the definitions of real and imaginary parts discussed above. The in-phase (real) part of the response is obtained by taking a reference sinusoid that is exactly in phase with the applied field, multiplying it by the velocity waveform and taking the average over all time. The out of phase (imaginary) part is obtained via the same procedure but using a sinusoid that is phase shifted by π/2 relative to the reference (cosine waves vs sine waves). Some experimentalists will recognize this as the same algorithm applied internally by lock-in amplifiers. It also corresponds to a Fourier transform at one discrete frequency. The results show exactly the expected behavior: all conductivity is imaginary, and it increases monotonically toward lower frequencies because the electron can reach arbitrarily high velocities (from a classical point of view) if the field maintains one direction for longer.

Now we take a step in the direction of real materials that have electrical resistance and real AC conductivity. Paul Drude used a simple addition of dissipation to eq 2 to describe the electrical conductivity of real materials:

3 $$m\frac{d^{2}x}{dt^{2}}+\frac{dx}{dt}m/\tau =-qE_{0}\cos\left(\omega t\right)$$

where the new term provides a force acting on the electron that is proportional to its velocity and opposes it. This is the “resistance”, and it corresponds to energy dissipation from inelastic collisions between the moving electron and other components of a real material, such as ionized impurities. The new variable, τ, corresponds to the average time between these collisions at the thermal velocity of the electron (*v*_therm_ = $\sqrt{k_{B}T/m}$ for 1D motion). Solving this equation analytically leads to the Drude formula of AC conductivity:^11,12^

4 $$\mathrm{\sigma ̂}\left(\omega \right)=\frac{q^{2}\tau }{m\left(1-i\omega \tau \right)}=\frac{q^{2}\tau }{m\left(1+\omega^{2}\tau^{2}\right)}+i\omega \frac{q^{2}\tau^{2}}{m\left(1+\omega^{2}\tau^{2}\right)}$$

which can be applied with remarkable success to describe the AC conductivity of bulk semiconductors and metals. Figure 1f shows the (numerically calculated) AC mobility as a function of frequency for this system. Crucially, whether the mobility (conductivity) of the material appears to be all-real or mostly imaginary depends on the probing frequency. If the rate of collisions causing dissipation is low compared to the period of the probing field, then the imaginary part remains dominant. If, however, there are many collisions per period of the probe, then the signal eventually becomes all real: the peak of the imaginary mobility appears at ω = 1/τ.

The third and final physical situation we will treat using this simple equation of motion is that of a bound charge with a harmonic restoring force. One can readily imagine either a charge in a shallow coulomb trap or a soft phonon mode responding in such a way at microwave (GHz) or THz frequencies:

5 $$m\frac{d^{2}x}{dt^{2}}+\frac{dx}{dt}m/\tau +kx=-qE_{0}\cos\left(\omega t\right)$$

The new term here is simply a Hookean spring force with force constant *k*. Figure 1e,f shows the corresponding solution with both positive and negative contributions to the imaginary mobility. This sort of behavior can be quite important for explaining systems with trapping or polar nuclear contributions.^13^ It is tempting to conclude that this equation would also capture the contributions of excitons to photoconductivity spectra. This is true, but only to a point. Exciton states generally possess resonances that are higher in frequency than the THz or GHz fields commonly employed experimentally, and their contribution usually shows up as a broadband change in the electronic polarizability of the sample.^14,15^ The approach to modeling this using eq 5 would be to take a sum over an ensemble of oscillators whose resonance frequencies are much larger than the region of interest.^16^ However, when exciton resonances *do* appear in THz spectra they manifest as quantized transitions like those shown in THz spectroscopy of GaAs quantum wells (see ref 17 and Figure 4).

These concepts (eqs 2–5) map equally well onto rotating dipoles in solution,^15^ or polar lattice fluctuations in a solid.^18^ The rotational analog of the linear equations are included in the supporting Python module for those interested. Indeed, any polar charge distribution can couple to an applied electric field, and its motion can be used to compute the expected response as a conductivity. A classical dipole that rotates freely leads to a purely imaginary response, just like the free electron (except in this case the rotations of a gas phase molecule will clearly be quantized and lead to discrete rotational spectra). Similarly, a hindered dipole whose rotational velocity is subject to scattering leads to an identical rotational analog of the response shown for linear motion in Figure 1f. These behaviors (polar nuclear motions versus the motion of electrons) are not distinguishable purely through AC conductivity measurements, leading to the ambiguity alluded to above. Is the sample response to an AC field really a “conductivity”, or not? Up until now we have not introduced the concept of the dielectric constant, or more generally the dielectric response function. Usually the dielectric response of a material is attributed to the polarization of polar nuclear structures or bound electrons, while conductivity is attributed to shared mobile electrons. This distinction is physically and conceptually important, but examination of Maxwell’s equations shows the same result stated above: electrical conductivity and permittivity are not actually distinguishable material properties from the point of view of an AC electromagnetic field. They are linearly interchangeable in Ampere’s Law,

6 $$\nabla \times H=J_{f}+\left(\sigma^{\prime }+i\omega {\text{ϵ}}_{r}^{\prime }{\text{ϵ}}_{0}\right)E$$

leading to the following equivalences:^19^

7 $${\text{ϵ}}_{r}^{\prime \prime }=\frac{\sigma^{\prime }}{\omega {\text{ϵ}}_{0}}$$

8 $$\sigma^{\prime \prime }=\omega {\text{ϵ}}_{0}{\text{ϵ}}_{r}^{\prime }$$

This equivalence and indistinguishability comes up more frequently in the TRMC literature than the THz literature, partly because polar modes are more common contributors at GHz frequencies than THz and partly because the THz spectrum allows better separation of these contributions based on their characteristic frequency response. We will return to this topic in discussing the advantages and disadvantages of typical THz versus GHz experiments.

### 2.2 Complex Conductivity from the Velocity Autocorrelation Function

Here we turn to the statistical mechanical linear response theory of AC conductivity and how it connects more sophisticated microscopic models with measured conductivity spectra. Most simply put, linear response theory is the idea that for small driving fields the response of the system is linear in the field strength (as is eq 1) and that *motions driven by the applied field are fundamentally the same as those that arise from thermal fluctuations*. This means that all information about the field response (conductivity) of an electron in the material can be inferred from the random thermal motion it exhibits without any applied field. This linear response approximation is generally valid for the AC conductivity measurements we focus on here, since most THz and GHz spectroscopy setups employ very low electric field strengths.

How low/weak does the field need to be for linear response to apply? The formal condition of the theory^20^ is that the system be “close to equilibrium”. In the present context of electronic transport this means that the work done on the electron by the applied field *between* scattering events must not exceed the average thermal energy exchanged *at* each scattering event, i.e., *qE* *l*_free_ ≤ *k*_B_ *T*, where *l*_free_ is the mean free path between scattering events. Compare this with a typical TRMC experiment. Here, the AC field amplitude is only 1000 V/m, which means that a carrier must drift 25 μm in the field in order to gain 1*k*_B_ *T* in kinetic energy at 300 K. In contrast, the mean-free path of electrons in common semiconductors varies from a few nanometers in metal halide perovskites to a few hundred nanometers in GaAs.

Consider Figure 1d–f again to understand how this works. We show an electron in a typical material, which scatters off defects at random. Between scattering events the electron has a definite velocity, but each collision sends it off in a new random velocity vector, consistent with thermal equilibrium. This is the essence of the Drude model of conductivity already described from the point of view of charge motion in an applied field. But in the absence of that field the electron is not at rest, it is traveling on a “random walk” with the average thermal velocity already noted, *v*_therm_ = $\sqrt{k_{B}T/m}$ for 1D motion. This random motion is characterized graphically by Figure 2a, where the velocity vector changes at random according to a characteristic scattering time (τ), in a distribution governed by Boltzmann statistics (velocities are drawn from a zero-mean Gaussian distribution with standard deviation, *v*_therm_). To characterize this behavior better we can calculate something called the “velocity autocorrelation function” (VACF). You take an arbitrary point in time to be *t*_0_ = 0 and then calculate how likely the electron is to be moving with the same velocity at some later time *t*:^21^

$$C_{\mathrm{VACF}}\left(t\right)=\left\langle v\left(t_{0}\right)v\left(t\right)\right\rangle =\frac{1}{N}\overset{N}{\underset{n=1}{\sum }}v_{n}\left(t_{0}\right)v_{n}\left(t\right)$$ 9

where the sum over *n* runs over *N* discrete trajectories. For a single electron on a single random trajectory, or even several, this seems to be nonsense. But when you average over many electrons in the material or many trajectories of the same electron (an ensemble), you find that a clear functional form emerges. These results are shown in Figure 2b. It should perhaps not be surprising that this particular VACF has the form of an exponential decay with the same time constant (τ) as was chosen for the characteristic scattering event rate (1 ps). Also note that the maximum value corresponds to the mean squared velocity of the electron undergoing random thermal motion (*v*_therm_^2^) rather than being normalized to 1 to express a probability of correlation. It turns out that all that is needed to calculate the conductivity of this ensemble of electrons is this VACF, using the relationship:

10 $$\mathrm{\mu ̂}\left(\omega \right)=\frac{q}{k_{B}T}\int_{t_{0}}^{\infty }\left\langle v\left(t_{0}\right)v\left(t\right)\right\rangle e^{i\omega t}\;dt$$

**Figure 2:**
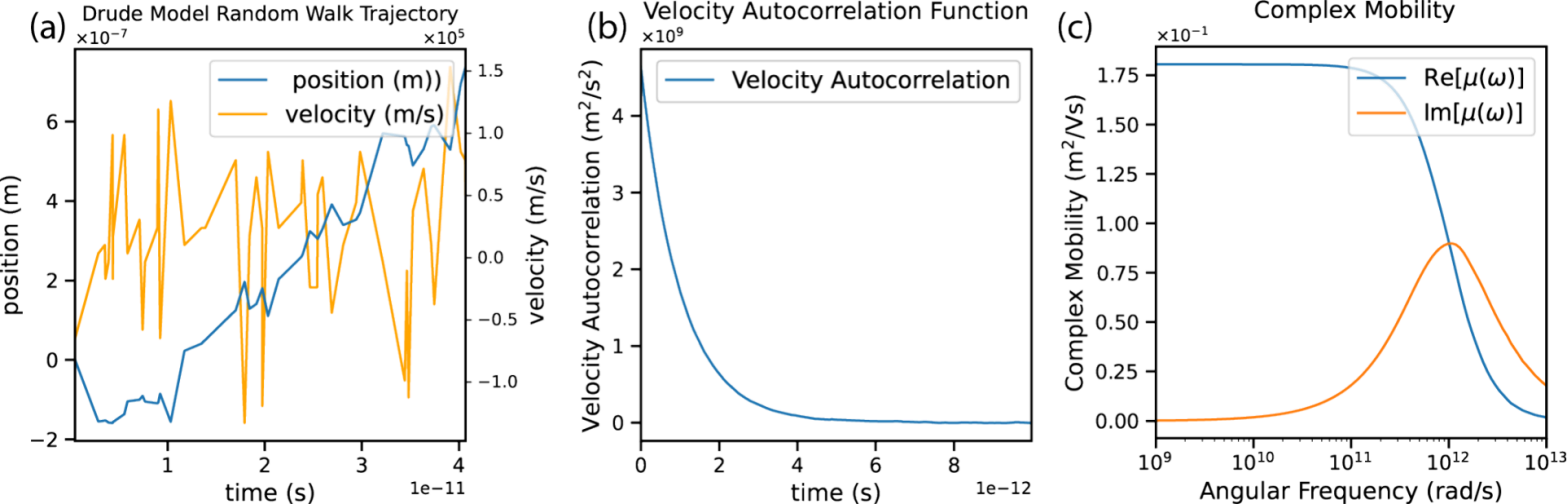
Example using the Kubo formula and the velocity autocorrelation function of a diffusing electron to calculate the complex mobility. (a) Short example segment of the electron’s position (*x*) and velocity (*v*) trajectory calculated by kinetic Monte Carlo with a characteristic scattering time of τ = 1 ps, just as in Figure 1e,f. (b) Corresponding velocity autocorrelation function (VACF) calculated from a much longer kinetic Monte Carlo (kMC) trajectory (1 × 10^6^ steps), showing the emergence of the exponentially decaying behavior with a time constant given by the mean scattering interval τ. (c) Complex mobility obtained by applying the Kubo formula to the VACF in (b).

The result is shown in Figure 2c and is *exactly the same* as what we calculated using the classical equation of motion in Figure 1f. Why is this true? At first it seems almost magical that any analysis of the random velocity fluctuations displayed Figure 2a could yield the same results as solving the equation of motion (eq 3). The full mathematical treatment of this result is beyond the scope of the present review, and the astonishing nature of this connection is a tribute to the brilliance of Melville Green,^22^ and Ryogo Kubo^20^ as well as Einstein,^23^ Smoluchowski,^24^ and Sutherland,^25^ who first realized this connection between thermal motion and ionic mobility in the form of the Einstein relation:

11 $$\mu =\frac{qD}{k_{B}T}$$

where *D* is the diffusion coefficient of the electron. Note that the integral of the VACF over all time *is* the diffusion coefficient, and the Kubo–Green equation (eq 10) reduces to the Einstein relation (eq 11) in the low-frequency limit.

The intuitive picture has already been noted. If an electron traverses a material full of scattering centers under the influence of a weak electric field, the field causes the velocity vector of the electrons to bend in its direction during their free-flight periods, leading to net charge motion in that direction. But they are still subject to the same frequency of scattering events as before the field was applied; as long as the field is weak, the perturbation to their motion is very slight. The mechanism that impedes the electron from flowing rapidly in the field direction is precisely the same scattering mechanism that generates the correlation function when the field is absent. Note also the information content of the VACF. Its peak value is the mean-squared thermal velocity of the electron, which reports on the effective mass thereof. The rate at which the VACF decays gives the scattering frequency, τ. These two parameters wholly determine mobility in the Drude model—though the Kubo formula is valid for any transport process that occurs in the weak-field (linear response) regime.

It is this latter point that makes the Kubo–Green relationship (eq 10) so useful. Any simulation that yields a VACF can be used to calculate the complex conductivity spectrum of the system, providing an easy way to employ much more sophisticated microscopic models of electrical transport in order to interpret AC conductivity spectroscopy results.

The reason that we present this point of view in the present section is that thinking of transport in this way provides a clearer physical picture of what exactly is happening in an AC conductivity measurement, and why the mobility is both complex and frequency-dependent. We are not forcing the electron through the material so much as watching its natural diffusion processes, and what we measure is determined by how “long” the measurement is, as determined by the period of the oscillating electric field we employ. Short observations (high frequencies) are more likely to “observe” free-electron-like motion that is mostly imaginary, while low-frequency measurements “observe” many collisions and lead to a real mobility. These outcomes underscore the importance of carefully considering what information about a sample, perhaps a novel semiconductor, a researcher is after when choosing GHz versus THz measurements, which we discuss in more detail in section 3.1.

Finally, we note that there is a third way of approaching this problem, which is often taken for its mathematical convenience. That is the impulse response method, in which the motion of the electron induced by an instantaneous impulse (a delta-function like force) is used to calculate the complex frequency-dependent conductivity (or mobility) via a Fourier transform. This is another general mathematical approach: any real signal in the time domain can be transformed to the frequency-domain through the Fourier series, and the result will generally be complex. Since the Fourier transform of a delta function (infinitely short impulse) is an even, infinite, distribution in frequency space, the time-domain impulse response completely characterizes the frequency-domain spectrum. We do not cover the impulse response formalism in any further detail here because it does not provide additional microscopic insight into the source of a given conductivity spectrum. Viewed the right way, however, the Kubo–Green analysis above is simply conceptualizing one arbitrary collision event as this impulse, and then averaging over a great many of them to obtain sensible results that are above the noise.

### 2.3 Model of Carrier Confinement in Nanostructures

The Drude model that we derived above from either the classical equations of motion or the velocity autocorrelation function turns out to be inadequate for fitting much experimental data. In particular, it was early observed in certain liquid metals that the real conductivity is peaked rather than monotonically decreasing with frequency.^11^ The so-called Drude–Smith model was originally derived to describe such behavior; the hypothesis was that there is a backscattering bias rather than the purely isotropic carrier scattering assumed in the Drude model.^12^ A so-called “backscattering” or “localization” parameter, *c*, was introduced via an impulse response formalism combined with an argument that the probability of such preferential backscattering followed a Poisson distribution.

The resulting Drude–Smith formula possesses the mathematical flexibility to fit most conductivity spectra of semiconductors adequately with just one additional parameter, *c*, but the physical meanings of *all* the parameters obtained by Drude–Smith fits become unclear.^26,27^ Even in the first instance considering liquid metals, the formula arises from truncating a series expansion in a way that is not physically justified.^12^ Its application to explain the behavior of nanostructured semiconductors is even more problematic. Much of modern materials science and chemistry research concerns nanostructured semiconductors, in particular that of emerging photovoltaic absorbers which often have limited grain or particle sizes in the early stages of material discovery. This confinement of the electron to a particle *also* results in peaked real (photo)­conductivity for the intuitive reason that DC (zero-frequency) conductivity must vanish if transport out of the particle is impossible. In this situation the backscattering probability does not follow Poisson statistics. Thus, if the goal of fitting conductivity spectra is to obtain accurate measurements for the carrier scattering time and the confinement length scale, fitting with the Drude–Smith model is fruitless. For this reason, we do not provide the formula in this review.

Fortunately, much effort has been devoted in the last 20 years to finding better alternatives, and these are now well-established. Kužel and Němec have recently reviewed this progress superbly,^17^ and we will not restate their full discussion here. Instead, we amplify a few of their key points and use examples from the Python module provided in the Supporting Information to illustrate the discussion.

There are two physical limits to consider: classical and quantum behavior. The conductivity spectra of most emerging semiconductors can be treated classically because carrier scattering is frequent enough that quantum transitions are masked even for small particle sizes. The fundamental requirement shown by Kužel and Němec^17^ for quantum effects to become dominant are that (1) the lowest-energy quantum transition (*E*_1_) must be larger than *k*_B_ *T* and the ratio of *ℏ* to the intrinsic scattering time (τ) (*E*_1_ > *k*_B_ *T* and *E*_1_ > *ℏ*/τ); (2) simultaneously there must be few random scattering events per average transit of the carrier across a confined grain or particle (the mean-free path must be equal to or larger than the typical grain or particle size). These conditions can be met in, for example, GaAs quantum wells, or possibly experiments studying emerging photovoltaic materials at cryogenic temperatures, but rarely in those conducted at room-temperature. We thus confine our brief discussion of models for interpreting conductivity spectroscopy to classical approaches.

The gold standard of classical models is kinetic Monte Carlo (kMC) simulation,^17,26,27^ as this is an exact numerical method. We have already introduced the general approach in the illustration provided by Figure 2. There, because of the details of the calculation (isotropic scattering on an infinite lattice) the result was Drude conductivity. The virtue of this approach is that any arbitrary process can be introduced without the potential need to find a new way of solving more complex equations. The kMC method always works; it is just a question of describing the desired physics correctly in the model and how long it takes to resolve the features of interest to the desired level of accuracy.

Figure 3 shows the autocorrelation functions (a) and complex conductivities (b) for three confinement regimes, calculated using the Drude kMC model provided in the supporting Python module. These correspond to confinement lengths, *L*, that are 1000, 50, and 10 × the mean-free path (*l*_free_), where the system is modeled in one dimension with perfectly reflecting boundaries. These calculations mirror reasonably those given previously,^26^ showing a peak developing in the real conductivity, an overall suppression of the measured AC mobility, and a shift of that peak to higher frequency as confinement is increased and boundary-scattering comes to dominate the overall scattering time of the system. Note the similarity between these conductivity spectra and those of the Drude–Lorentz system of Figure 1g–i but also that it is weaker in forcing the low-frequency conductivity to zero and asymmetric in shape. Hence the term “weakly confined” systems to describe free carriers confined to a nanostructure, as distinct from electrostatically bound carriers that respond as Lorentz oscillators.

**Figure 3:**
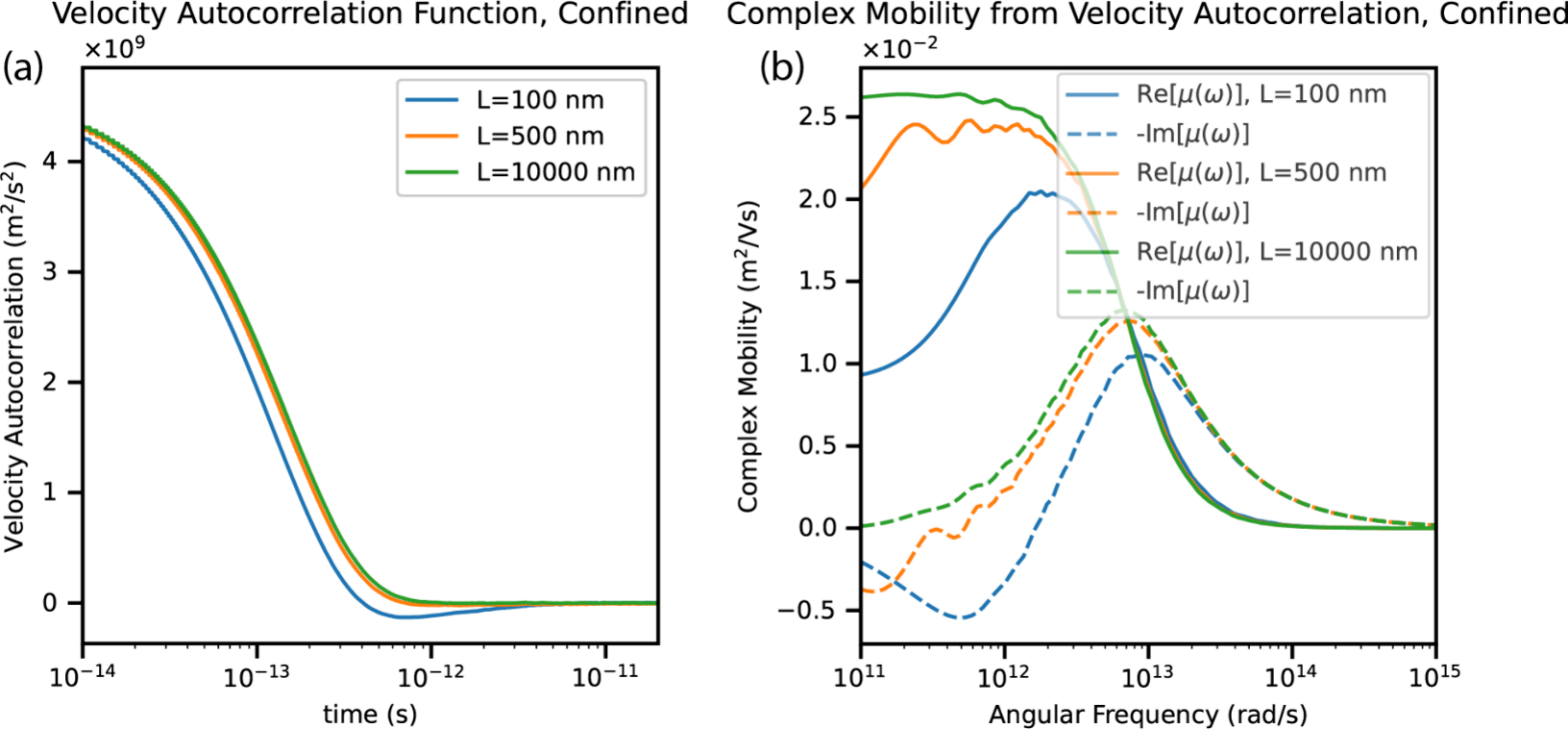
kMC simulations from the Python module in the Supporting Information, showing 1D simulations of (a) the velocity autocorrelation function and (b) the corresponding complex mobility. The intrinsic scattering time was chosen (τ = 148.3 fs) in this 1D simulation such that the mean-free path, *l*_free_, is 10 nm. Thus, the overall confinement lengths of 100, 500 and, 10,000 nm correspond to *L*/*l*_free_ = 10, 50, and 1000. Ten million time steps were used to calculate each trajectory. The oscillations that are particularly evident for *L* = 500 (but present in all the traces) are caused by finite integration bounds on the VACF. In practice, a careful balance must be found between a too-long VACF integration, which introduces excessive noise, and a too-short one, which causes spurious oscillations.

One of the early rational explanations of this low-frequency suppression of conductivity due to physical confinement arises from the TRMC literature rather than THz spectroscopy.^28^ There, the authors only had access to a single (or a few) microwave frequencies, but employed varying lengths of conjugated polymers to elucidate this phenomenon rather than measuring the conductivity spectrum in detail. Their model is derived by coupling diffusion equation solutions to the Kubo formula, and thus does not directly invoke the scattering time of the electron at all, but does serve to model how the observed AC mobility is suppressed by physical confinement.

A notable difference between their results and later Drude simulations is that the real conductivity increases monotonically with frequency for a given confinement length before saturating, and cannot reproduce the high-frequency decline in mobility predicted by Drude models—a consequence of not including the fundamental transport physics. This is appropriate when the scattering frequency in the material is far higher than the experimentally accessible radiation, which is often the case in TRMC experiments. The model has been very successfully employed to explain confinement effects in both conjugated polymers,^29–31^ fullerene clusters,^32^ covalent organic frameworks,^33^ hybrid halide perovskite grains,^34^ and emerging Zintel phase absorbers.^35^ More recently, we have found good agreement between this confined diffusion model (CDM) and the more recent “modified Drude–Smith” formula (MDS)^27^ in studying the doping-induced conductivity of single-walled carbon nanotubes in solution at GHz frequencies.^36^ However, one important discrepancy that arises between the kMC and MDS models on the one hand, and the behavior of the CDM on the other is that the former models predict negative imaginary contributions to the photoconductivity signal, whereas the CDM does not.

The review by Kužel and Němec^17^ contained an excellent figure comparing the more modern closed form models of conductivity spectra to full quantum mechanical and kMC based descriptions. This figure is reproduced as Figure 4. Here they showed that although the Drude–Smith model can fit the kMC and quantum-mechanical calculation, the parameters produced do not match those input to the numerical simulations. In contrast, the MDS model^27^ and a newer semiclassical (SC) formula^38^ both perform much better and do not include variable parameters different from those driving the simulations. As these newer models are straightforward to implement there is little justification for using the Drude–Smith model in circumstances where physical confinement can reasonably be expected to influence the conductivity spectra. We thus make the strong recommendation that either the MDS^27^ or SC^38^ model be used in future analyses of complex conductivity spectra, especially in cases where the goal of the experiment is to extract meaningful physical properties in an emerging semiconductor. These models are implemented for the reader’s convenience in the Python modules in the Supporting Information. While the full quantum and kMC approaches no doubt remain the most accurate, both the human and computational effort involved in implementing them and fitting data necessarily reconcile them to niche studies where this more detailed approach is truly required.

**Figure 4:**
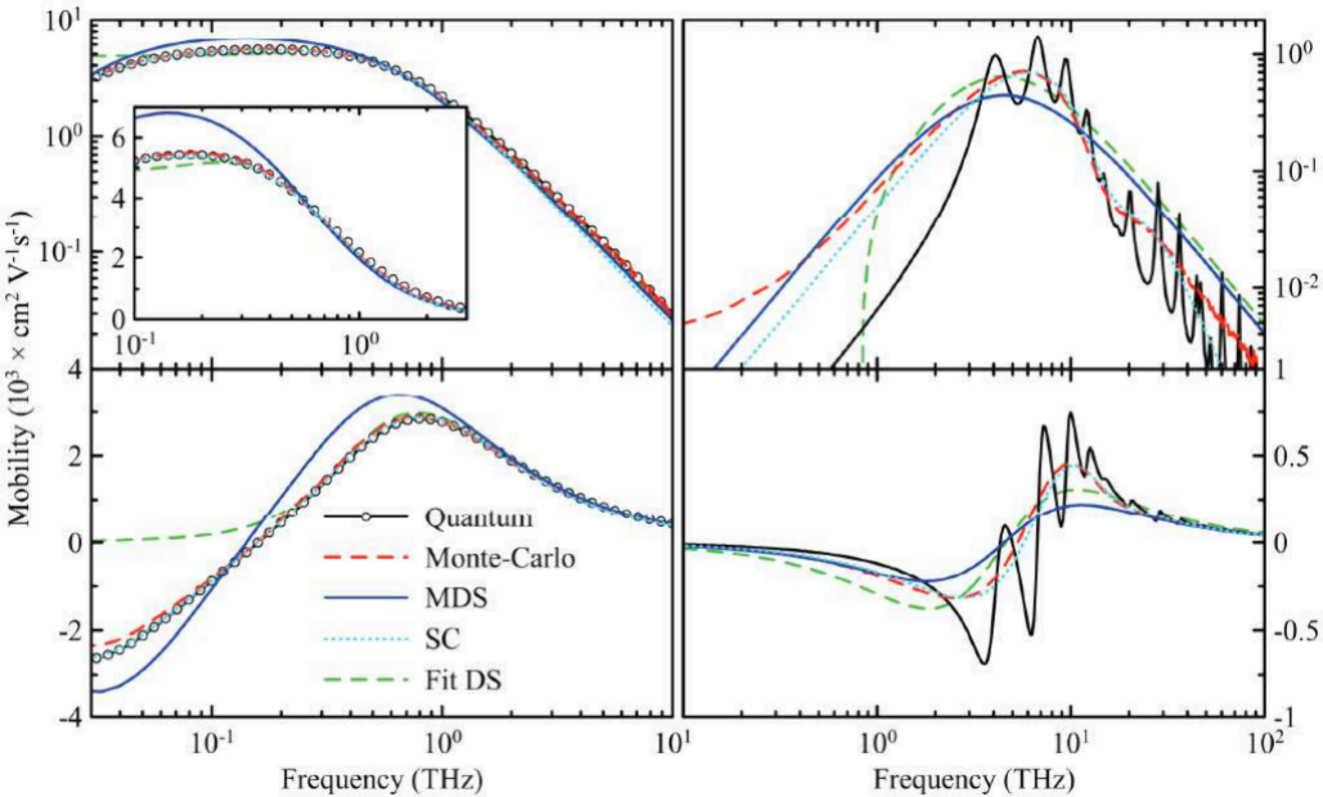
Calculated real (top) and imaginary (bottom) part of the mobility of cubic-shaped GaAs nanocrystal with carrier scattering time τ = 270 fs, temperature *T* = 300 K, and NC size *d* = 1024 nm (left panel), 32 nm (right panel). Various models are used: black solid line and symbols, full quantum model μ_QC_;^37^ red dashed line, Monte Carlo simulations μ_MC_;^26^ solid blue line, modified Drude–Smith model μ_MDS_;^27^ dotted cyan line, semiclassical model μ_SC_;^38^ green dashed line, fit of μ_MC_ by the Drude–Smith model.^12^ The figure and caption were reproduced with permission from ref 17. Copyright 2020 Wiley-VCH.

### 2.4 Charge Carrier Kinetics and Why τ_PL_ ≠ τ_σ_

Equally important to understanding the origin of conductivity spectra are charge carrier kinetics. The spectroscopist is often asked whether one method, (e.g., time-resolved photoluminescence, TRPL) is “better” than another (e.g., TRMC) for measuring carrier lifetime. The answer generally is “both”. This is because these different transient measurements nearly always provide complementary information.^39^ One must consider the differences in information about charge carrier dynamics each technique provides.

Figure 5 provides an important case in point. The Herz and Johnston team constructed a combined TRMC/TRPL spectrometer based on a quasi-optical approach^40^ rather than the more typical waveguide-based approach to handling microwave signals (which we discuss in the next section).^41,42^ This allows them to more easily utilize an optical cryostat to simultaneously obtain TRMC, TRPL, and PL spectra as a function of temperature. The key point made in Figure 5c,d, however, is that TRMC kinetics and TRPL kinetics are not the same, even if measured with the same laser pulse, simultaneously, as shown here. Instead, they are different manifestations of the interconnected kinetic processes of carrier transport, recombination, and trapping. As a rule, photoluminescence signals are proportional to the *rate* of charge recombination (*dn*/d*t*, d*p*/d*t*), whereas photoconductivity signals are proportional to the *concentration* (*n*, *p*). When the carrier kinetics is first-order (decays exponentially), this is a difference without a distinction; an exponential is its own derivative. However, this is neither the ideal circumstance nor a common one.

**Figure 5:**
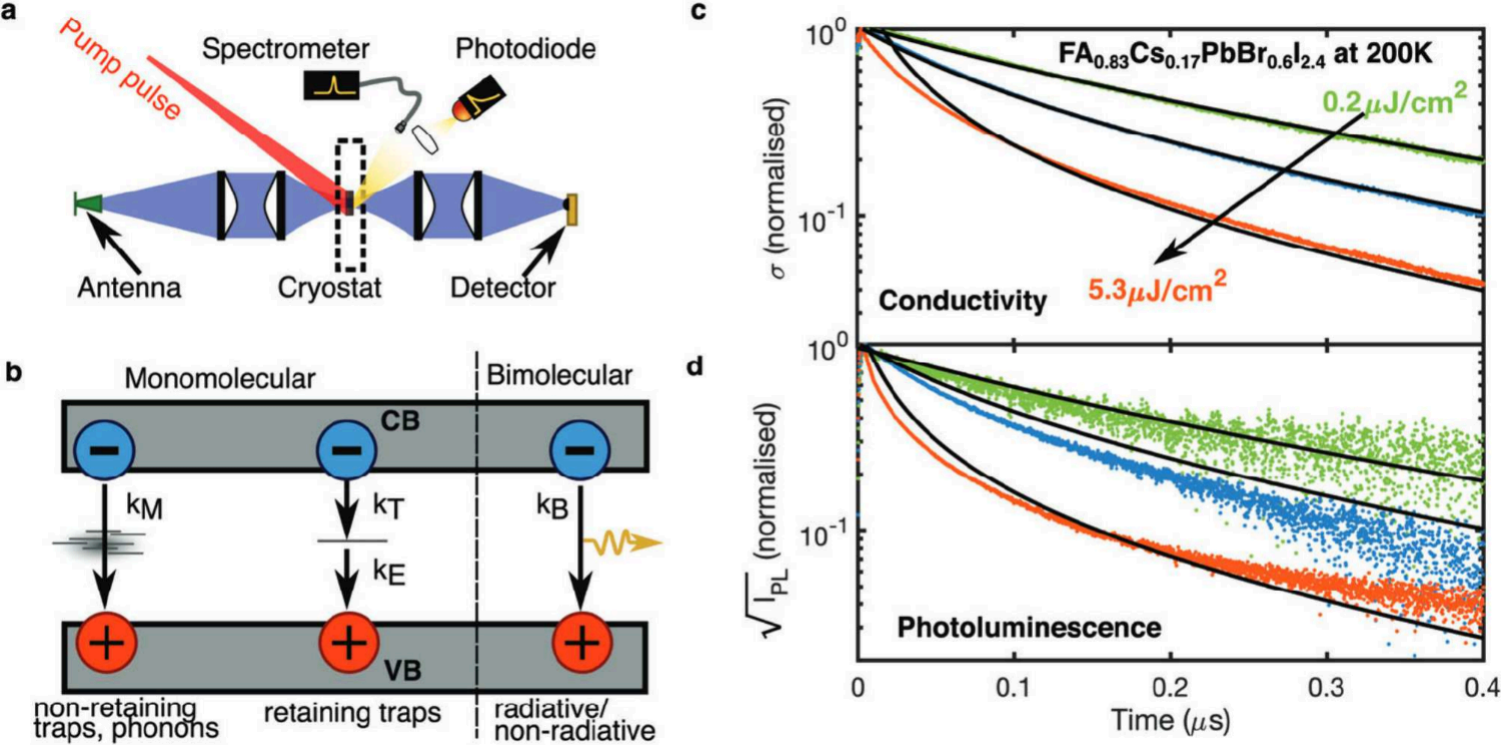
(a) A simplified diagram of the combinatorial experimental setup used to acquire photoconductivity decay traces, TRPL data, and PL spectra in situ, showing the feed horn antenna for launching microwave radiation into free space, the fibre-coupled spectrometer and a free-space fast photodiode for PL measurements, and the Schottky diode used to detect transmitted microwave (MW) radiation. (b) An illustrated model describing charge-carrier recombination in metal–halide perovskites, encompassing monomolecular and bimolecular recombination routes, and differentiating between chargeretaining and non-retaining traps. The model is used to fit the microwave photoconductivity decay traces shown in (c) globally across different excitation fluences (indicated in the color of the data points) and the obtained parameters of the fits (shown in black solid lines) were used to predict the photoluminescence decay dynamics shown in (d). Such predicted PL transients are shown as solid black lines on top of the experimental data (presented as the square root of the PL intensity) with the comparison enabling an assessment of the validity of the assumptions presented in the model. The figure and caption were reproduced from ref 40. CC BY 4.0.

Instead of being compared 1:1, photoluminescence and photoconductivity measurements need to be connected and modeled through an appropriate kinetic scheme. In the case of Figure 5c,d, they use the following system of equations:

12 $$\frac{dn}{dt}=-\gamma_{r}np-k_{m}n-k_{T}n$$

13 $$\frac{dn_{T}}{dt}=k_{T}n-k_{E}n_{T}p$$

where *n* and *p* are the concentrations of free photogenerated electrons and holes, respectively, and *n*_T_ is the trapped electron population. Free charge carriers recombine radiatively via the rate γ_r_ *np*, decay nonradiatively via the rate *k*_m_ *n*, and are trapped at the rate *k*_T_ *n*. The trapped electrons then also recombine with the rate *k*_E_ *n*_T_ *p*. Note that here *p* = *n* + *n*_T_ (since the number of photogenerated electrons and holes must be equal) and the generation term representing the input laser pulse has been omitted.

The TRPL and TRMC kinetics are then calculated by solving this scheme numerically, where the TRMC signal is proportional to

14 $$\Delta \sigma \left(t\right)=q\left[\mu_{e}n\left(t\right)+\mu_{h}p\left(t\right)\right]$$

whereas the TRPL is proportional to

15 $$I_{\mathrm{PL}}=\gamma_{r}n\left(t\right)p\left(t\right)$$

In this particular case, the authors fit the TRMC kinetics to the model and then calculated what the corresponding PL should be, producing good agreement. Note the square-root *Y* axis of the PL that makes the TRPL kinetics look more similar to the TRMC kinetics than is actually the case.

This general approach, combining photoconductivity and photoluminescence kinetics has been very broadly applied and quite successful at extracting detailed kinetic parameters that help understand the fundamental processes that govern carrier kinetics in emerging photovoltaic materials.^43–49^

## 3 Fundamentals of Terahertz and Microwave Conductivity Measurement Hardware

At this point, we hope the reader has a foundational understanding of what AC conductivity measurements can provide. Here we summarize typical experimental implementations, contrasting microwave and terahertz methods. Thus, far we have discussed microwave and terahertz spectroscopy mostly as one. This is appropriate from a fundamental physical perspective: both experiments measure the complex conductivity of a sample by passing low-energy electromagnetic radiation through it and analyzing the transmitted waveform, possibly as a function of time after photoexcitation (or other stimulus). In both cases careful electromagnetic modeling either from numerical solutions to the electric wave equation^41^ or another approximate theory,^19,50^ are needed to recover the complex conductivity from changes in the transmitted or reflected waveform. The distinction between them only arises when we come to discuss their hardware implementations, which are *very* different.

In this section we do not aim to provide full detail on either TRMC or TRTS/OPTP implementations. Numerous previous reviews have covered that ground very well for both microwave^41,42^ and terahertz conductivity.^50–53^ Instead, we use this section to show how these experiments are both like, and unlike one another; discuss the reasons a researcher might choose one over the other, and highlight some recent innovations of importance for studying emerging photovoltaic materials.

### 3.1 Comparison of Typical Microwave and Terahertz Experiments

Figure 6 shows a comparison of typical (simplified) hardware setups for both microwave (left) and terahertz (right) photoconductivity measurements. The key elements shown in each case are (1) probe radiation source, (2) technique of radiation transfer to the sample, (3) probe detection and time-resolution method, and (5) typical data outputs. See the figure caption for further detail.

**Figure 6:**
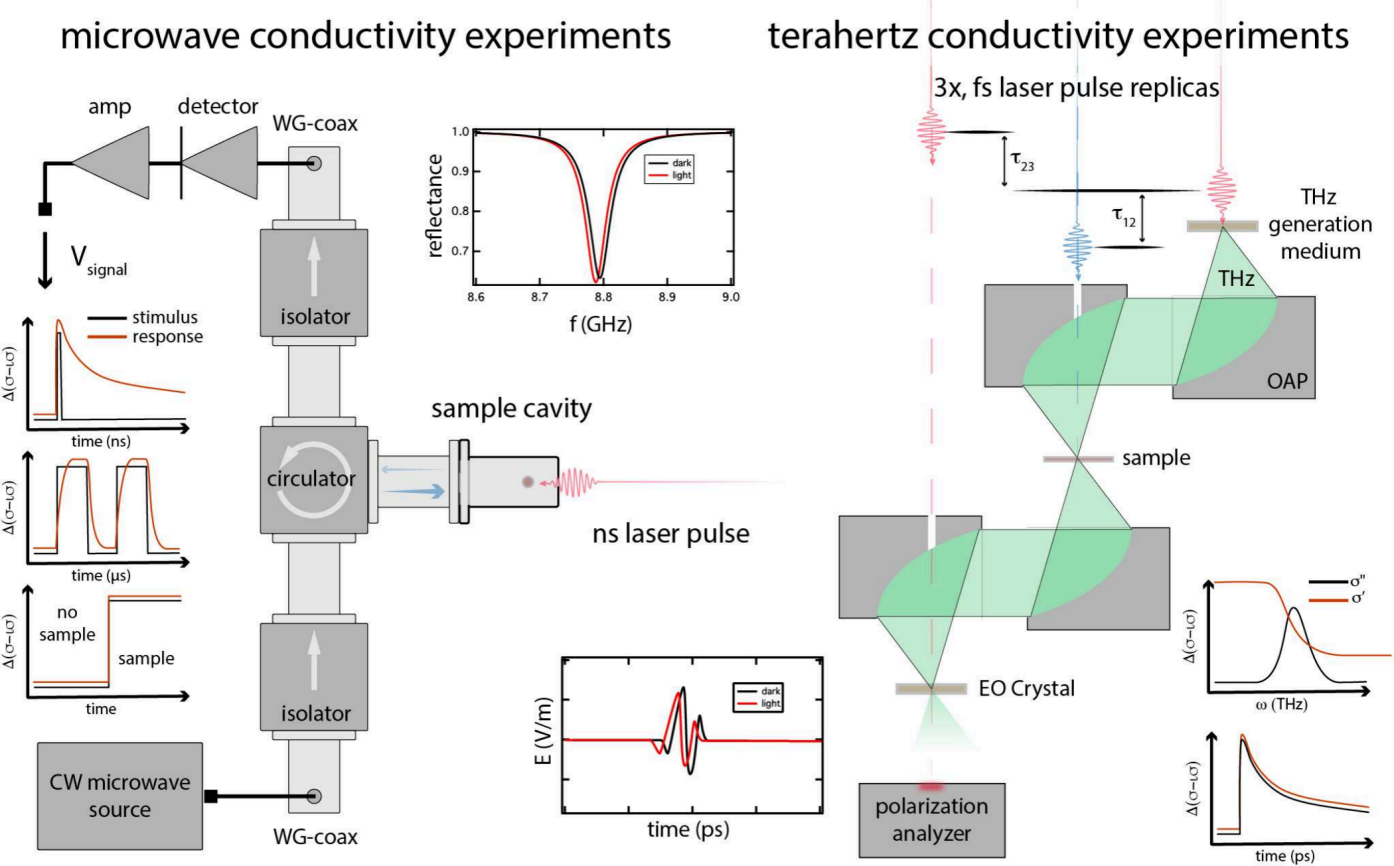
Typical experimental apparatus for (left) microwave conductivity and (right) terahertz conductivity experiments. Although terahertz and microwave conductivity operate using very different hardware implementations, their fundamental physical principles are the same. Electromagnetic radiation is passed through a sample, and the transmitted wave is analyzed to measure the equilibrium or transient complex conductivity. Typical microwave conductivity measurements use a CW microwave source at a single frequency or narrow band of frequencies and waveguide structures to handle the radiation. A cavity structure is generally used to enhance sensitivity, and the real and imaginary parts of the conductivity (at a single frequency) can be obtained by analyzing the shift in resonator properties as a function of sample introduction or excitation. Transient signals are directly resolved in the (nanosecond) time domain with a fast diode detector and digitizer. TRTS/OPTP uses an ultrafast laser pulse interacting with a nonlinear electro-optical (EO) crystal, a photoconductive antenna, or a spintronic emitter material to generate a short broadband THz pulse. Quasi-optical methods are used to relay the pulse through the sample and to an EO crystal, wherein the THz electric field strength modulates the birefringence of the crystal and is thereby directly sampled in the time domain using another ultrafast laser pulse replica whose polarization rotation is detected as a function of time delay (τ_23_, sometimes called τ_TDS_). A third pulse replica, possibly frequency shifted via an OPA, serves to excite the sample, and time-resolved data are obtained by varying the delay between it and the probe pair (τ_12_, sometimes called τ_pump_).

Each of these instruments is capable of three related experiments that go by different acronyms, but which are all just different facets of the same measurement. Terahertz time-domain spectroscopy (THz-TDS) is the act of using just the probe generation and analysis pulses to measure the equilibrium conductivity spectrum of the sample, usually by comparing the THz transmission spectrum through the sample to that through a reference. Optical-pump terahertz-probe (OPTP) spectroscopy consists of choosing one delay in the THz-TDS system (corresponding to the time-domain *E*-field maximum, for instance) and resolving its time evolution relative to an optical pump using standard pump–probe methods. Time-resolved terahertz spectroscopy (TRTS) is a combination of the two, where the THz-TDS delay is scanned at (at least) some subset of the pump–probe time delays identified from OPTP measurements. TRTS can also be employed at every time delay to create a 2D map of how the conductivity spectrum evolves in time, but this is often prohibitively time-consuming.

In microwave experiments we often refer to dark microwave conductivity (DMC), time-resolved microwave conductivity (TRMC), and time-resolved dielectric loss (TRDL) (often also just called “TRMC”).^15,54,55^ These map neatly onto the definitions of THz-TDS, OPTP, and TRTS given above. DMC is the act of comparing the microwave cavity resonance characteristics with and without a sample of interest to obtain its complex equilibrium conductivity. TRMC consists of monitoring the time-evolution of microwave power reflected from the cavity at a single microwave frequency. TRDL adds the frequency dimension, measuring TRMC signals over a set of microwave frequencies to time-resolve the full evolution of cavity characteristics and thereby recover the time-dependent complex conductivity, though still effectively at a single frequency rather than the spectrum obtained in TRTS.

A key point connected to these different hardware implementations is the relative cost, complexity, and ultimately the suitability of these two measurements for achieving different scientific objectives. THz spectroscopy has some significant advantages in capability over single-frequency microwave measurements, which come with a corresponding increase in cost and experimental complexity. Primarily:

1. THz measurements provide a complex conductivity *spectrum* whereas standard microwave conductivity implementations only provide a single frequency measurement. The spectrum is very useful for evaluating fundamental transport characteristics of a material, such as momentum scattering time and physical confinement, as already discussed.
2. THz measurements provide picosecond time resolution, whereas TRMC is typically limited to ≥0.5 ns. The greater time resolution of THz spectroscopy is often important for understanding dynamic carrier localization, cooling, or excitonic trapping processes.

On the other hand, all forms of THz measurements based on the THz-TDS probe *require* an ultrafast laser system, and OPTP and TRTS involve timing three ultrafast pulses. Microwave measurements in contrast employ robust CW sources of probe radiation and mere electronic delays to achieve nanosecond resolution. Many practical advantages for microwave conductivity measurements flow from this difference in design:

1. Microwave conductivity measurements are much higher in sensitivity (at least 100×) relative THz-TDS based measurements due to the use of a cavity structure. This allows low-fluence (pump energy) transient measurements that minimize the influence of higher order annihilation processes that sometimes mask the signals of interest.
2. Microwave conductivity setups are much lower cost. The basic microwave hardware can be had for ∼$15k, with simple nanosecond lasers at an additional $10–15k, and the scope to digitize signals at ∼$20k. This total system cost (∼$50k) is ∼1/4 that of just the ultrafast laser needed for TRTS, and likely to be ≤1/10th the cost of an overall TRTS setup.
3. Simplicity confers high experimental throughput. Microwave conductivity systems can easily be engineered to be turn-key instruments, making them more accessible to scientists who are not dedicated laser spectroscopists but require conductivity spectroscopy for their research objectives.
4. Microwave conductivity systems require very little maintenance, and optical beam alignment is extremely simple due to the practice of expanding the laser beam to fill the waveguide cross-section in which the sample is mounted.

Clearly, these are complementary experiments. Microwave experiments excel in projects where a large family of samples needs to be investigated,^56–59^ and/or the high sensitivity is very important.^58,60^ They are also very suitable for high-throughput experiments in automated lab environments, and as a standard characterization tool for materials scientists and chemists who need fast feedback on the optoelectronic properties of their materials, especially when processing affects performance.^61^ Terahertz experiments excel at providing fundamental physical details of transport processes via the shape of the conductivity spectrum, and, for example, the ultrafast dynamics of carrier cooling and interaction with phonon modes. When it comes to the question of which to use or which is better, the right answer comes down to what scientific objective you have and what you want to know about the samples involved.

### 3.2 Recent Innovations in Measurement Configuration

In this section we address recent innovations that have had an impact on the diversity of samples that can measured, or the information that can be obtained, primarily using microwave conductivity, but encompassing terahertz experiments as well. We do not treat recent advances in microwave or terahertz technology, but instead focus on physical measurements of photovoltaic and related materials. A surprising outcome of applying these techniques to an expanding breadth of material and semiconductor types is that the relative difficulty of connecting ultrafast time scale conductivity dynamics to applied properties has pushed technique development in the direction of “ultraslow” experiments (described next), where measuring the equilibrium or steady-state properties of photovoltaic materials on the relatively slow time scale of micro- and milli-seconds turns out to be highly informative for semiconductor material discovery and development.

#### 3.2.1 Dark Conductivity

A surprisingly important development in our own lab and others in the last ten years has been the standardization of equipment, analysis methods, and sample configuration for dark microwave conductivity experiments. We and others have established standard geometries and simulation packages that allow interrogation of the dark equilibrium properties of thin-films,^18,62–65^ solutions,^15,36,54^ semiconductor powders,^35,66–69^ and single crystals.^41,70^ These experiments allow us to, for example, establish the carrier mobility, doping density, and dielectric constant of new emerging photovoltaic absorbers before thin film samples are available if TRMC and DMC are applied in concert.^35,68^ Another example of the surprising power of this approach comes from Tom Savenije’s group, who used DMC to measure the oxidation induced doping density of Cs_0.25_FA_0.75_Sn_0.5_Pb_0.5_I_3_, quantifying the influence that SnF_2_ treatment has on the electronic carrier density of the resulting thin films.^71^

Note, however, that while some papers refer to equilibrium measurements in the dark as “steady-state microwave conductivity (SSMC)”,^18,71,72^ we propose that this phrase (SSMC) should be reserved for situations involving slowly modulated or continuous illumination that drive the sample to kinetic *steady state*.^65,73^ This latter technique (slow optical modulation that achieves quasi-steady-state conditions) has been employed more and more frequently of late and will be discussed further in the following section.

THz-TDS has also been used quite extensively to measure the dark equilibrium conductivity spectrum of emerging photovoltaic absorbers, with a focus on Sn-based perovskite formulations because of the large conductivities that they often present through adventitious doping caused by Sn­(II) → Sn­(IV) oxidation.^74–77^ Importantly, it was through this work on Sn-perovskite compositions that the Hertz and Johnston team discovered standard thin-film approximations used for computing the THz conductivity from the transmitted waveform accumulate serious errors when the dark conductivity of the sample becomes too high. They propose, and verify through numerical simulation, a new formula that yields much more accurate results under these conditions.^19^

Comparing the DMC and THz-TDS results serves to highlight the trade-off being made when one chooses terahertz versus microwave spectroscopy. The THz-TDS spectra allow unique physical insight including resolving phonon modes and assessing whether the conductivity is in fact Drude-like.^75,77^ On the other hand, the sensitivity limit of the DMC approach employed by Savenije’s team^71^ on similar materials shows sensitivity to carrier concentrations down to ∼10^15^ cm^–3^, whereas that reported via THz-TDS is limited to a detection limit of ∼10^18^ cm^–3^ in this case.^74,75^ Notably, it is the similarity of these perovskite compositions that allows such a direct comparison in terms of carrier density, as the measured quantity, σ = μ*Nq* is always a product of mobility (μ) and carrier density (*N*).

#### 3.2.2 Steady-State Photoconductivity

While time-resolved measurements such as OPTP and TRMC are extremely useful, it sometimes happens that the processes governing photovoltaic or photoelectrochemical device performance operate on a time scale well beyond what is typically accessed in these experiments. This may be particularly true in perovskite formulations that undergo significant ion migration during operation,^78^ or in photoelectrochemical applications where the chemical reaction at the surface to form the product (e.g., H_2_) is intrinsically slow.^79^

Steady-state photomodulation measurements provide a very sensitive way of extending microwave—and potentially terahertz—photoconductivity experiments to microseconds–seconds time scale. This is because these measurements are conducted in the frequency domain, where long-lived signal contributions are enhanced in amplitude and one can take full advantage of the noise rejection offered by lock-in amplification. In contrast, in transient time domain experiments long tails are always hard to measure due to the overall decay of signal amplitude, and there are intrinsic limitations of optical delay lines, and contributions from the ambient 1/*f* noise spectrum.

These experiments consist of amplitude modulating an optical excitation beam (sometimes white light, sometimes monochromatic) and recording the change in microwave absorption using a lock-in amplifier referenced to the modulation source. This is directly analogous to “frequency-domain fluorometry” and we refer the interested reader to an excellent textbook that describes this approach to spectroscopy in detail.^81^ To our knowledge this specific methodology has not been used to study the steady-state terahertz photoconductivity spectrum of emerging photovoltaic materials, and so the remainder of this discussion is confined to microwave experiments.

We originally developed this methodology in our lab for the express purpose of collecting microwave photoconductivity action spectra, wherein photoconductivity is recorded as a function of excitation wavelength—akin to recording a photocurrent action spectrum of a solar cell but without the need for a complete device.

Our implementation of this technique (steady-state microwave conductivity, SSMC) employs a much higher quality factor resonator than typically used for TRMC (*Q*_SSMC_ ≈ 1000 vs *Q*_TRMC_ ≈ 200) and a homodyne circuit that differs from that shown in Figure 7 in that it includes a reference arm to form a microwave interferometer.^80^ Design considerations for tuning resonator quality factor have been detailed previously.^41^ The higher quality factor and the homodyne circuit combine to substantially improve signal/noise ratio. We have since used this approach to understand charge transfer between perovskite solar cells and an organic charge transport layer,^82^ and study band tailing in promising BaCdP_2_ photovoltaic materials.^35^ More recently we have used a white light LED to investigate the modulation-frequency dependence of the signal to study, e.g., organic photovoltaic composites under photoelectrochemical conditions.^83^

**Figure 7:**
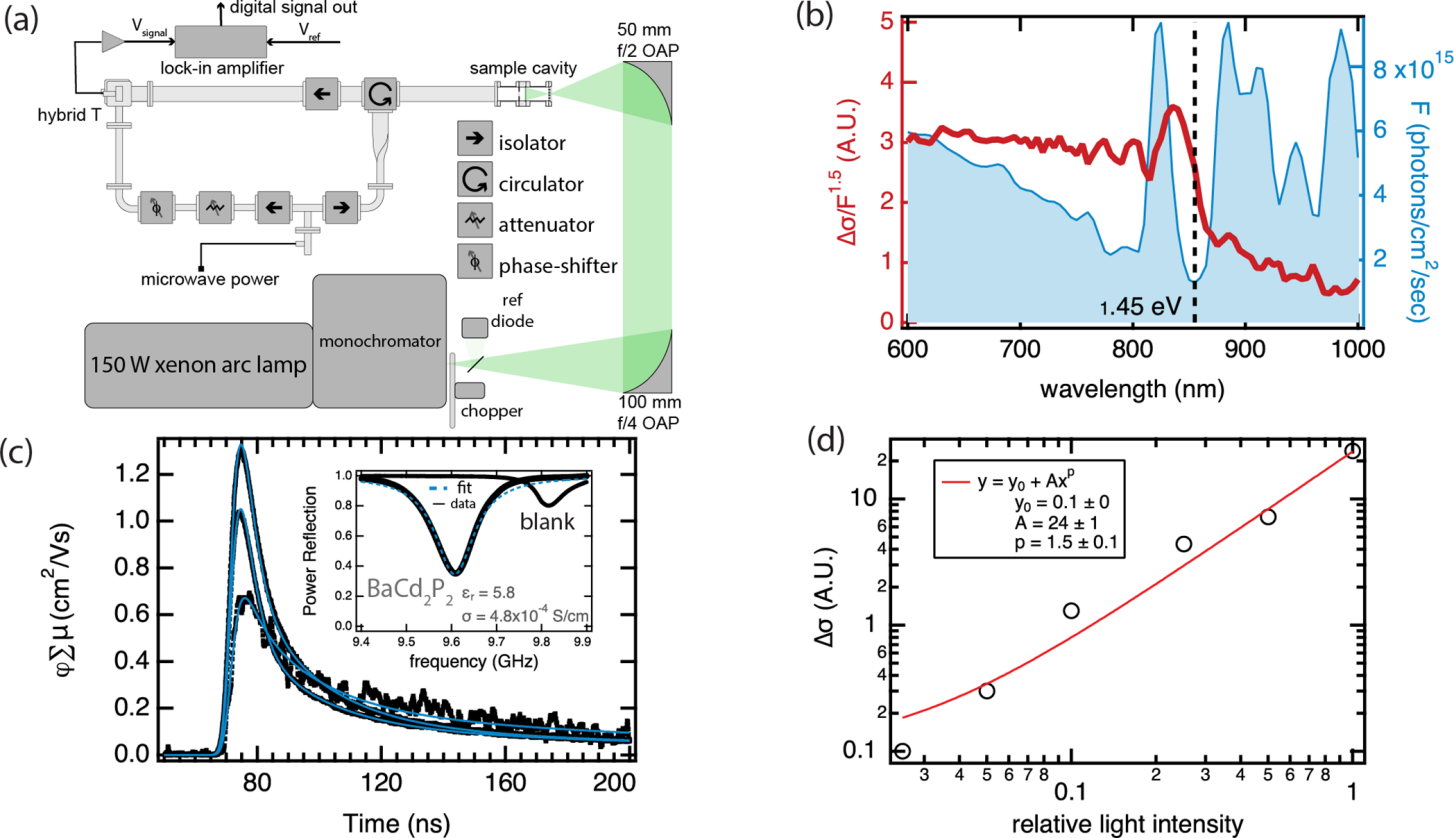
(a) Diagram of the microwave homodyne circuit used to conduct steady-state microwave conductivity (SSMC) experiments at NLR. (b) Steady-state photoconductivity action spectrum normalized by the linearized photon flux (red line, left axis) and the incident photon flux (blue shaded area, right axis) used in this measurement. The calculated absorption edge is shown via the black dashed line at 855 nm/1.45 eV. (c) Three representative photoconductivity transients (black) and triexponential fits (blue) as a function of laser fluence (600 nm, 7 ns fwhm), expressed as the product of carrier yield (ϕ) and mobility sum (Σμ). The inset shows the microwave cavity resonance curves for the empty EPR tube (blank) and that filled with the sample (BaCd_2_P_2_ powder). The real relative dielectric constant and AC conductivity that correspond to the fit curve (blue dashed line) obtained via electromagnetic modeling of the cavity are noted in the inset. (d) Intensity-dependence of the steady-state photoconductivity signal (circles), fit with a power law (red trace). The *y*-intercept was fixed at the experimentally noted baseline signal, while the power and prefactor were freely fit to the data. The power law provides the linearized flux with which the spectrum in (b) is normalized to remove lamp intensity variations from contributing to the extracted spectral shape. (a) Reproduced from ref 80. Copyright 2019 American Chemical Society. (b–d) Reproduced with permission from ref 35. Copyright 2024 Elsevier.

The Labram and Savenije groups have also developed similar capabilities, though based more closely on the original TRMC circuit design.^65,73,84^ Both groups have demonstrated the ability to simultaneously measure steady-state and time-resolved microwave conductivity. Notably, Labram employed this methodology^85^ to study the light-soaking evolution of FA_0.83_Cs_0.17_Pb­(I_0.9_Br_0.1_)_3_ (FACs) and a triple-cation composition: (FA_0.83_MA_0.17_)_0.95_Cs_0.05_Pb­(I_0.9_Br_0.1_)_3_ (FAMACs), where FA is formamidinium and MA is methylammonium. On both of these high performance materials the photoconductivity steadily rises over 18 h of illumination. Because they cleverly combine the TRMC and SSMC experiments, it is clear that the mobility is constant, and this increase must be attributed to a light-soaking induced increase in steady-state carrier concentration. Moreover, because these were contactless measurements, they prove that light-soaking induced increases in the photovoltaic performance of these compositions cannot be attributed to external DC bias stress, or other effects of the contact layers—the evidence suggest signal shifts are intrinsic to changes in the perovskite structure/composition during illumination.

#### 3.2.3 Light-Biased Transient Photoconductivity

The combination of SSMC and TRMC described in the last section is closely related to the more common practice of introducing a continuous wave bias illumination (BI) into transient measurements in order to assess whether the transient dynamics are likely to be different under steady-state operating conditions of a solar cell.^86^

These sorts of measurements are quite important. While purely transient dynamics obtained from an equilibrium baseline can sometimes be used to derive a complete kinetic model that makes correct predictions of photovoltaic performance at steady state,^87^ it is hard to ensure that the scheme is truly complete. Testing that the kinetic model can reproduce the dynamics of both transient perturbations from steady state as well as from equilibrium provides a very useful check.

This principle has been demonstrated quite clearly by Savenije’s team in the study of perovskite/contact layer interfaces. In one example they compared SpiroOMeTAD and C_60_ contact layers for MAPbI_3_.^65^ Only with a light bias present do they observe clear distinctions between the active layer kinetics in comparing the C_60_ ETL layer to SpiroOMeTAD. They found that the C_60_ layers prevent the development of a large trap population under bias illumination, that takes place in both bare MAPbI_3_ and that with Spiro. Additionally, with Spiro the recombination rate constant increases under bias light. This is a case where not only do the kinetics change under bias illumination due to the increased carrier density, but *rate constants* also change because the material is dynamically changing under illumination.

In another example, Savenije’s team studied charge extraction from Cs_0.05_MA_0.10_FA_0.85_Pb­(I_0.97_Br_0.03_)_3_ (CsMAFA) to three different transport layers (Spiro, C_60_, and PTAA, in different configurations) and used bias light and steady-state microwave conductivity to show how these different layers influence the quasi-Fermi level splitting under constant illumination.^73^

These results highlight the importance of using a bias light, in particular when the goal is to compare contact layers and understand how they influence device performance. Without the bias light TRMC experiments reveal the kinetics due to displacement from, and recovery to equilibrium, which is dominated by charge transfer between the active layer and the contact(s). When the bias light is present, TRMC can be used to observe the kinetics dominant at steady state, and reveal otherwise obscured trapping and recombination processes that the contact layers influence.

In the THz space, the Johnston and Herz team has used bias illumination to study light-induced halide segregation in MAPb­(I_0.5_Br_0.5_)_3_ by OPTP.^78^ Here, the primary influence of the bias light is quite different from that investigated by Savenije’s team. The bias light drives halide segregation in the film without noticeably changing the charge carrier density and connected dynamics because the pump fluence for OPTP is (26.6–1.5 μJ/cm^2^, 400 nm; 5 × 10^12^ to 3 × 10^11^ cm^–2^) is high enough to create a transient carrier density much larger than that caused by the CW bias light. Instead, they observe enhanced phonon features in the THz spectra for the iodine segregated domains that suggest enhanced anharmonicity of the lattice.

Multiple transient pulses have also been used to obtain similar information to that derived from light bias experiments. For instance, a novel dual pulse excitation (DPE-)­TRMC experiment has recently been used to reveal pernicious long-lived, immobile, trapped charges in Cs_2_AgBiBr_6_.^88^ Here, Savenije’s team employed two nanosecond laser pulses and digital difference detection to investigate the influence that the first carrier population has on the next excitation event. They find that there are very long-lived trap states which can be filled by the first pulse, causing an enhancement in the magnitude of the second one. In contrast, the influence of the first pulse is negative for MAPbI_3_, because the previously generated electrons only bleach the transition and/or accelerate recombination.

In the same vein, optical pump-push–THz-probe experiments have revealed *negative* real photoconductivity in either light doped, or chemically doped FA_0.83_Cs_0.17_SnI_3_.^89^ These results were attributed to stimulated THz emission (SE) as these interband (push) excitations lead to nonthermal carrier populations that relax on a ps time scale. The shape of the photoconductivity spectrum is Lorentzian for both light induced and chemically induced carriers, consistent with previous observations of THz SE.^89^

Interestingly, negative real photoconductivities have also been observed in mixed-composition Sn-based perovskite materials using microwave conductivity, but in these cases the negative contribution often appears delayed in time,^66,90^ much too long to be attributed to hot carrier relaxation and stimulated microwave emission. In these cases this behavior has been attributed to a reduction in dielectric loss caused by trapped charges that “stiffen” the lattice.

As with the dark conductivity section above, the comparison in findings from TRMC versus OPTP studies involving light bias and/or multipulse experiments is very illustrative of their respective applications. The kinetics observed in light biased TRMC experiments are highly sensitive to presence and intensity of the bias light, because the transient carrier density is a small perturbation (10^9^ cm^–2^) on top of the carrier density induced by the bias light. The OPTP measurements with their much higher injection fluence (5 × 10^12^ to 3 × 10^11^ cm^–2^) are better suited to studying the changing structure of the material itself, or resolving ultrafast processes such as carrier cooling. Again, the choice of which technique to use boils down to which one aligns with the scientific objective at hand, and the experimental information required to achieve it.

## 4 What Conductivity Spectroscopy Reveals About Photovoltaic Materials

The core capability of conductivity spectroscopy is to reveal the lifetime and yield-mobility product of photogenerated charges without the need to apply electrical contacts, and there is a very large body of literature that does just that. We do not attempt to summarize all of these reports here, but rather focus on studies or analysis approaches that use conductivity spectroscopy to provide maximal physical insight. It turns out that combining conductivity spectroscopy with a modest number of other measurements (e.g., photoluminescence) can provide *all* the information one needs to assess the quality and potential of a photovoltaic material.

### 4.1 Quasi-Fermi Level Splitting

The quasi-Fermi level splitting achieved under 1-sun illumination is a vital characteristic of any solar absorber material, as it gives the upper limit for the open-circuit voltage that can be obtained in a device. Importantly, it is the open-circuit voltage that has the most room for improvement across a broad range of photovoltaics materials, with CdTe and Organic Photovoltaics having the largest gap to close before reaching the theoretical efficiency limit.^91^ In the silicon world noncontact photoconductivity tools have long been used to obtain such “implied” open-circuit voltage values,^92^ but only recently have researchers begun using TRMC or OPTP measurements to do the same.^87,93^ The basic equation is^92,94^

16 $$\mu_{F}=\frac{k_{B}T}{q}\;\ln\frac{\left(\Delta n+n_{0}\right)\left(\Delta p+p_{0}\right)}{{n_{i}}^{2}}$$

where μ_F_ is the quasi-Fermi level splitting (eV), Δ*n* and Δ*p* are the additional electron and hole concentrations (cm^–3^) that result from illumination, *p*_0_ and *n*_0_ are the thermal equilibrium electron and hole concentrations, and *n*_i_ is the intrinsic carrier concentration. Note that the product *n*_0_ *p*_0_ satisfies *n*_0_ *p*_0_ ≡ *n*_i_^2^ but that *p*_0_ and *n*_0_ may each inversely deviate from *n*_i_ due to either intentional or adventitious doping. Using this equation, any measurement that can quantify the carrier density established at steady state under 1-sun illumination can be used to estimate μ_F_.

There are two basic approaches that one can take to calculate μ_F_ from photoconductivity data. Either a kinetic model can be developed from fitting transient data^87,93,95^ and then solved to steady state to find the relevant carrier densities (Δ*n*, Δ*p*), or a steady-state photoconductivity measurement under 1-sun illumination can be combined with the mobility obtained from TRMC or OPTP to calculate it from more direct observations.^65,73,87^ This is quickly becoming a very important application of both TRMC and SSMC measurements and it is worth amplifying certain details.

A first look at eq 16 suggests that *n*_i_, *p*_0_, and *n*_0_ may be unknown and difficult-to-measure quantities. In fact, they turn out to be not so challenging. *p*_0_ and *n*_0_ are often very small and can be neglected when the injection density is high enough.^73^ If not, they may be readily measured via DMC (or THz-TDS) methods combined with TRMC (or OPTP) measurements or obtained as kinetic fit parameters to injection-density-dependent transient data.^96^

The intrinsic carrier concentration (*n*_i_) is more problematic. It has been estimated to be between 10^3^ and 10^6^ cm^–3^ for a variety of perovskite compositions^97,98^ (ref 73 used 10^6^ cm^–3^ in their calculations). It has also been calculated from effective masses drawn from band-structure calculations (*m*_e_^*^ = 0.16 and *m*_h_^*^ = 0.15)^99^ using^94^

17 $$n_{i}=\sqrt{N_{C}N_{V}}e^{-E_{g}/2k_{B}T}$$

where *N*_C_ and *N*_V_ are the effective densities of states in the conduction and valence bands, respectively, given by

$$N_{C}={2\left(\frac{2\pi m_{e}^{*}k_{B}T}{h^{2}}\right)}^{3/2}$$ 18

$$N_{V}={2\left(\frac{2\pi m_{h}^{*}k_{B}T}{h^{2}}\right)}^{3/2}$$ 19

The effective mass can also sometimes be measured by specialized time-resolved THz methods.^100,101^

However, the most straightforward noncontact method to measure *n*_i_ is to combine a carrier density measurement like SSMC/TRMC with external radiative efficiency (ERE) under the same illumination conditions. Then *n*_i_ can be calculated by measuring Δ*n*, Δ*p*, *n*_0_, *p*_0_, and the absorbed flux from the light source (*G*_s_), derived from the extinction coefficient spectrum of the semiconductor (α­(*E*)) and the flux spectrum of the light source.

In the simplest case, the carrier density measured under illumination at steady state is related to the net recombination rate coefficient by

20 $$\Delta n\Delta p+\Delta np_{0}+\Delta pn_{0}=\frac{G_{s}}{\gamma_{r}+\gamma_{\mathrm{nr}}}$$

where γ_r_ and γ_nr_ are the radiative and nonradiative rate constants for charge recombination. A measurement of the external radiative efficiency (ERE) allows the equation

21 $$\mathrm{ERE}=\frac{\gamma_{r}}{\gamma_{r}+\gamma_{\mathrm{nr}}}$$

to be solved for γ_r_, which then allows *n*_i_ to be calculated for any given temperature by the overlap between the blackbody radiation spectrum and the absorption spectrum of the material using^102^

22 $${n_{i}}^{2}\gamma_{r}=G_{0}\left(T\right)$$

where *G*_0_(*T*) is the generation rate of electron–hole pairs in the material due to absorption of blackbody radiation. Algebraic simplification of eqs 20–22 gives

23 $${n_{i}}^{2}=\frac{G_{0}\left(\Delta n\Delta p+\Delta np_{0}+\Delta pn_{0}\right)}{G_{s}ERE}$$

where we have dropped the explicit argument “(*T*)” from *G*_0_. In the large injection limit, where Δ*p* = Δ*n* ≫ *p*_0_, *n*_0_, this equation simplifies to

24 $${n_{i}}^{2}\approx \frac{G_{0}\Delta n^{2}}{G_{s}ERE}$$

Substitution of eq 23 for *n*_i_^2^ into eq 16 leads to an expression for μ_F_ that depends only on the measured ERE and absorption spectrum (via *G*_s_ and *G*_0_), as the carrier density terms cancel:

25 $$\mu_{F}=\frac{k_{B}T}{q}\;\ln\left(\frac{G_{s}}{G_{0}}\cdot \mathrm{ERE}+1\right)$$

Moreover, by combining eq 25 with eq 23 and again taking the high-injection limit, it can also be shown that

26 $${n_{i}}^{2}\approx \Delta n^{2}e^{-q\mu_{F}/k_{B}T}$$

which is the more commonly used result for calculating *n*_i_.^103^

Equation 25 for the quasi-Fermi level splitting may also be obtained in fewer steps from the detailed balance equation for the ideal current density–voltage (*J*–*V*) characteristics of a solar cell^102,104^ when it is expressed using the same variables as above:

27 $$J\left(V\right)=\left[G_{s}-\frac{G_{0}}{\mathrm{ERE}}\left(-1+e^{qV/k_{B}T}\right)\right]qL$$

wherein the only new variable is *L*, the semiconductor thickness. Setting *J*(*V*_OC,SQ_) = 0 and solving for *V*_OC,SQ_ gives

28 $$V_{\mathrm{OC,SQ}}=\frac{k_{B}T}{q}\;\ln\left(\frac{G_{s}}{G_{0}}\cdot \mathrm{ERE}+1\right)$$

thus proving that our derivation of eqs 23–26 is equivalent to the detailed balance model.

Clearly, ERE measurements alone are sufficient to calculate μ_F_ = *V*_OC,SQ_. However, combining ERE and carrier density measurements allows direct determination of *n*_i_ and γ_r_, whereas ERE alone does not. Moreover, once established for a given semiconductor, the *n*_i_ or γ_r_ values determined in this way enable photoconductivity experiments to be used to estimate μ_F_ independently, and these are often easier to accomplish under broadband solar illumination than ERE, where the emitted light can be very hard to quantify.

### 4.2 Diffusion Length

Diffusion length, *L*_*D*_, is another vital characteristic of a photovoltaic material, which characterizes the average distance that charges diffuse before they recombine. It can be used to calculate the fraction of photogenerated charges that will be collected for a given device thickness. At steady state in one effective dimension, the diffusion length is given by

29 $$L_{D}=\sqrt{D\tau }$$

where *D* is the diffusion coefficient and τ is the effective lifetime of the charges *at the charge density of an operating solar cell*.^65^ Note the slight difference here from the value one expects from the statistical random walk, which predicts *L*_D_ = $\sqrt{2nD\tau }$ , where *n* is the dimensionality. The difference arises because the former description is for the decay length of the concentration profile established at steady state (appropriate to a solar cell in operation), whereas the latter describes the time-dependent displacement after a transient injection event.

It is immediately evident that TRMC and OPTP measurements are germane, particularly with an illumination bias applied to simulate 1-sun operating conditions. In the simplest case, the transient dynamics provide the lifetime, and we can calculate the diffusion coefficient from the mobility using the Einstein relation (eq 11). It turns out to be slightly more complicated than this statement implies, but not dramatically so. There are three complications that we phrase as “which lifetime?”, “which mobility?”, and “what about grain boundaries?”. We address them in order.

Even under steady-state illumination the transient dynamics may not be single exponential, making the selection of τ ambiguous, leaving aside whether one should use a photoconductivity or photoluminescence lifetime.^93^ Connected with this, it is sometimes found that the peak mobility measured by OPTP is a *lot* higher than that measured by TRMC. This can arise either because confinement effects in finite semiconductor grains lead to increasing mobility with frequency (as we showed in Figure 3 and the connected discussion) or because the mobility itself (e.g., via the effective mass)^89^ is time dependent. Such dynamic localization is very commonly observed in metal oxides of interest for photoelectrochemical applications^105–108^ and certain “perovskite inspired” semiconductors of recent years.^109–114^

Fortunately Hempel’s team has discovered a rather elegant solution to the first two problems.^115^ It turns out that you do not need to know whether photoconductivity decay is due to a loss in carrier density or a time-dependent mobility, nor is it necessary to assign a single characteristic lifetime in order to calculate the diffusion length. Starting from the diffusion equation they derived and numerically verified, they obtained a simple average expression that can be used to calculate *L*_D_ by direct integration of photoconductivity transients using

30 $$L_{D}\approx 0.75\sqrt{2\frac{k_{B}T}{q}\int_{0}^{t}\frac{\Delta \sigma_{\Sigma }\left(t^{\prime }\right)}{\Delta n_{s0}}\;dt^{\prime }}$$

where Δσ_Σ_(*t*′) is the measured sheet photoconductivity including the sum (Σ) of electron and hole contributions (as is always the case in TRMC and OPTP measurements), Δ*n*_s0_ is the initially injected sheet carrier density, and the other symbols have their usual meaning.

The only reason that this is an approximate expression is the above-mentioned limitation that electron and hole mobilities are not independently measured (the assumption here is that they are balanced, μ_e_ = μ_h_) and that there is a subtlety with regard to whether the shape of the carrier distribution is Gaussian or exponential. The latter results in only 6% possible error,^115^ and eq 30 provides the average expected result. Verification of this description through comparison with numerical simulations is given in Figure 8.

**Figure 8:**
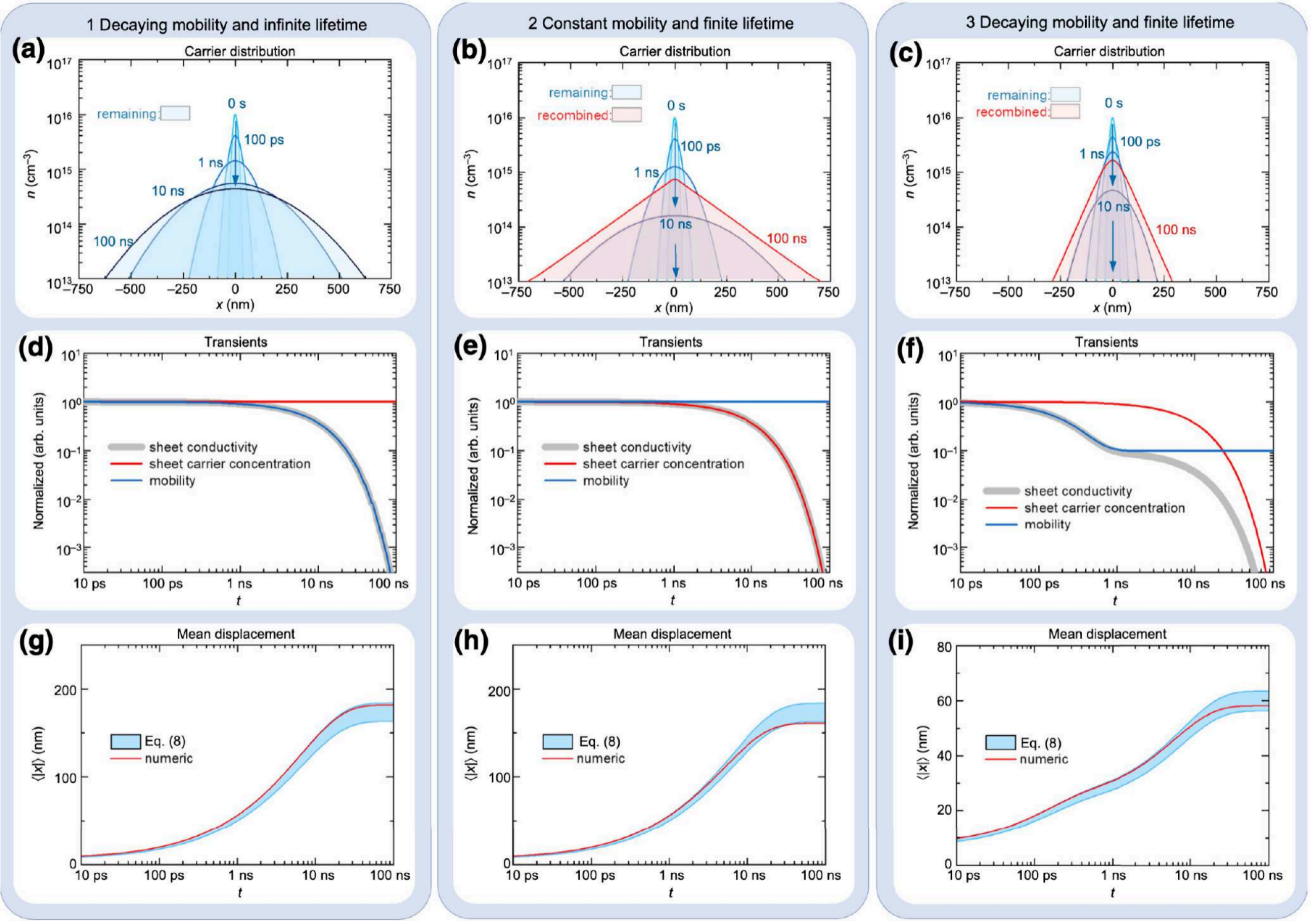
Confirming the generalized diffusion length by numerical modeling. (a–c) Evolution of Gaussian carrier distributions modeled numerically with eq 1 in ref 115 for (a) mobility decaying with a decay time, τμ, of 10 ns from an initial value of 1 cm^2^ V^–1^ s^–1^; (b) constant mobility of 1 cm^2^ V^–1^ s^–1^ and a lifetime, τ, of 10 ns; and (c) mobility decaying with a decay time of 100 ps from an initial value of 1 cm^2^ V^–1^ s^–1^ and a lifetime of 10 ns. Final distribution of the recombined carriers is estimated at *t* = 100 ns, ≫τ, and is shown in red. (d–f) Normalized transients of the mobility (blue line), sheet carrier concentration (red line), and sheet conductivity (thick gray line). (g–i) Evolution of the mean absolute displacement. Precise numerical value (red line) is, in general, between the values of analytical eq 8 in ref 115 with the factor $\sqrt{1/2}$ < *a* < $\sqrt{2/\pi }$ . For decaying mobility and without recombination (case 1), *a* = $\sqrt{2/\pi }$ . For constant mobilities and recombination (case 2), it converges to a diffusion length with a factor *a* = $\sqrt{1/2}$ . The figure and caption were reproduced from ref 115. CC BY 4.0.

Hempel’s team further tested this expression experimentally by stitching together both OPTP and several variants of TRMC experiments to characterize the whole time domain from picoseconds to milliseconds for crystalline silicon (c-Si), amorphous silicon (a-Si:H), a triple-cation lead halide perovskite, and bismuth vanadate (BiVO_4_).

The remaining unaddressed challenge is that of grain boundaries. We have already discussed at length how grain boundaries can localize transport, and lead to mobilities that increase markedly with probe frequency (Figure 3 and associated discussion). Hempel’s team showed this quite clearly as well in follow up work on grain/particle size limited systems, where there is a clear discontinuity between TRMC and OPTP transients.^107,114^ We suggest caution with any form of diffusion length calculation where such signs are clearly evident, either from combined TRMC/OPTP studies or from experiments that employ grain-size variations.^34^ The CDM, MDS, or SC models (defined in the models section) could be used to compute the intrinsic (unconfined) mobility with which to renormalize the photoconductivity data and compute *L*_D_ via eq 30. However, this would be a notional value of what the diffusion length might be in a single crystal of the same material. Simply integrating the measured photoconductivity data (eq 30) would lead to a lower value, but probably still overestimate the *actual* diffusion length present in a nanocrystalline film. In the absence of other data we suggest comparing the calculated diffusion length to the observed grain size in structural measurements, and conservatively assuming that the smaller of these two is the most representative of *L*_D_. Many emergent semiconductors are characterized as disordered, multiphasic, and/or nanostructured, underscoring the importance of the how and why in this section when evaluating the diffusion length of photogenerated carriers in your sample.

### 4.3 Yield–Mobility Product in Organic Photovoltaics

Organic photovoltaics are a special subcategory of materials because the semiconductors involved do not intrinsically generate free electron–hole pairs upon photoexcitation.^116^ Their charge carrier mobility is also nearly always much lower (10^–4^ to 10^–1^ cm^2^ V^–1^ s^–1^) than that observed in hybrid perovskites (10^–2^ to 10^2^ cm^2^ V^–1^ s^–1^) or other bulk inorganic semiconductors (10^1^ to 10^4^ cm^2^ V^–1^ s^–1^). This puts sensitivity at a premium and makes microwave spectroscopy a more natural choice, although terahertz experiments are also used.

In this context, the fact that we measure Δσ­(*t*) ∝ ϕΣμ­(*t*) with conductivity spectroscopy becomes quite important. In 3D organic–inorganic perovskites, semiconducting oxides, and other inorganic semiconductors the assumption that ϕ = 1 (every absorbed photon produces a free electron hole pair, even if trapping or exciton formation follows later) is generally a good one. When it comes to organic semiconductors, it usually is not.^116^ A practical outcome of these differences translates to “when” along the research timeline is most effective “to use” techniques like TRMC. In the case of hybrid perovskites, yields are assumed 1 and mobilities are high enough that processing conditions significantly affect both peak photoconductivity and the transient lifetime, unlike OPVs. As such, TRMC is highly beneficial in assisting with optimizing the processing conditions for a fixed perovskite composition, while it is biggest impact for OPV is in identifying which highly promising active layer compositions to pursue in device fabrication.

In this section we describe both progress in using conductivity spectroscopy for the discovery and development of new organic photovoltaic composites using ϕΣμ­(*t*) as the figure of merit, and how microwave measurements of ϕ (once μ is known) are providing unique physical insight into how these devices operate.

#### 4.3.1 ϕΣμ­(*t*) for Fundamental Mechanistic Studies

A unique capability of all conductivity spectroscopy methods is the ability to investigate samples that do not have bicontinuous transport networks, or even electrodes. We have used this capability to its utmost in work at NLR, aimed at understanding the fundamental operating principles of organic photovoltaics. This vein of work was launched in ca. 2012, with the discovery of Marcus-like^117^ inverted behavior in the photoconductivity of model organic photovoltaic composites.^56^ These experiments were conducted by dilutely sensitizing a series of different conjugated polymer donors with a homologous family of fullerene acceptors, taking advantage of the fact that after electron transfer only one of the two charges is mobile in the host film.

Subsequently, Marcus-like inverted behavior has been demonstrated in a truly diverse array of organic semiconductor systems: carbon nanotube donor films sensitized by fullerene acceptors;^57^ polycrystalline pentacene donor films (triplet exciton states) sensitized with (primarily) perylene diimide acceptors;^58,60^ fullerene acceptor films sensitized with phthalocyanine and naphthalocyanine donors.^59,118,119^

The key design feature in all of these studies is the isolated donor and the simplification of the yield mobility product Δσ­(*t*) ∝ ϕΣμ­(*t*) to ϕμ_e,h_(*t*). While this simplification may seem trivial, it has the key impact of eliminating morphological changes in one phase of the donor–acceptor system as a reason that the photoconductivity signal might vary in magnitude.

Ultimately, conducting experiments on samples where we *know* the GHz-frequency mobility in the host material have proved crucial. Our study of the fullerene host matrix^59^ showed that the inverted regime we observe—while it is caused by the Marcus rate equation—actually arises more directly from a competition between long and short-range electron transfer events.^119^

This was a case where *only* conductivity spectroscopy could have been used to conduct such work; in particular only TRMC possess the combination of selective sensitivity to mobile charges and a noncontact nature such that dilute samples could be measured. Both our model of this phenomenon^59,119^ and experimental efforts to observe similar effects in devices^120^ suggest that it is masked in device structures by the high density of states available for charge transfer. Similarly, our work on charge separation from triplet states on pentacene, relevant to solar cells beyond the detailed balance limit,^121^ *required* the low excitation fluence accessible via TRMC due to efficient triplet-charge annihilation processes in that system.^58,60^

One of the things that this work highlights is the crucial need to measure the mobility independently from the carrier yield in organic semiconductors. It is fortunate therefore that work is well underway to solve this problem by combining various kinds of device structures with GHz and THz measurements to access the AC mobility. These efforts began with a charge modulation based approach,^122–127^ and have since diversified to include time-of-flight (TOF),^128^ and current extraction by linearly increasing voltage ramp (CELIV)^129^ experiments conducted simultaneously with TRMC measurements. Notably, a recent report has extended this idea into the THz range.^130^

#### 4.3.2 ϕΣμ­(*t*) as a Figure of Merit for Organic Photovoltaics

Organic semiconductors, the underlying active layer materials in organic photovoltaics (OPVs), organic light-emitting diodes (OLEDs), organic field effect transistors, and other organic optoelectronic devices, exhibit an infinite design space for their molecular or polymeric structures. Although the expansive applications of organic semiconductors are not the focus of this review, the situation of “how to progress faster” toward technologies built upon such an infinite semiconductor design space is useful to discuss in the context of conductivity spectroscopy. To those deeply familiar with the field of organic electronics it remains an inspiring fact that OLEDs and OPVs, for example, are so mature while at the same time the deepest details of their operating physics exist in ongoing debate.^59,118,119,131–133^ Conductivity spectroscopy has, and should continue to, accelerate progress on both the fundamental and applied fronts of organic semiconductor technology.

History shows that material design evolution for organic semiconductors is the common thread across all major technological advances in the field of OPVs. Chlorophyll-mimicking porphyrins gave way to semiconducting polymers, whose design continues to evolve, fullerenes came and went, “small molecule” donors and acceptors have become very *large* molecules, and the building block approach of linking variations of electron rich and electron poor subunits in molecule and polymer forms has expanded dramatically. Power conversion efficiencies across all OPV active layer material-types (polymer-molecule, all polymer, all molecule) are now in the 18–20% range. History also shows that device optimization for new OPV active layers is a long (roughly 7 years on average), expensive, and effort-intensive process. Traditionally, device fabrication and optimization is required to evaluate new OPV materials for performance, but material structure/design is inextricably bound to device processing variables. This situation results in slow, random and often serendipitous decision-making on the design of new champion OPV molecular structures. Since evaluating new molecular designs involves the long and expensive task of device optimization, it is clear to see why progress speed is limited by the ability to ‘vet’ new structures in the vastness of available options. Conductivity spectroscopies like TRMC can shift the paradigm on the traditional cycle of progress.

With respect to functional material and device-relevant properties, we have focused on GHz microwave conductivity to evaluate the temporal resolution of electrical processes occurring on the nanosecond to microsecond time scale, relevant to photocurrent generation and collection in devices. In basic terms, an OPV film that exhibits efficient photon-to-charge yields, long mobile carrier lifetimes, and high mobilities (all measured directly by TRMC, for example, via ϕΣμ­(*t*)) exhibits the same properties that predict high performance *potential* in a photovoltaic material. Rapid assessment of material performance (RAMP) is a screening protocol developed at NLR that incorporates fast and high-fidelity spectroscopies to evaluate whether a photoactive semiconductor is promising enough to invest the substantial time and effort involved with turning a newly discovered or developed semiconductor into a high-performance material around which functional devices can be optimized, up front. Using RAMP, only two measurements, UV–vis–NIR and TRMC, are carried out on a standalone film (fabricated using less than 5 mg of semiconductors) in order to decide whether the process of device optimization is warranted for a new OPV active layer. Since electron scattering rates are very high in organic semiconductor solids, and AC mobilities are low, ϕΣμ­(*t*) is practically microstructure-independent in many materials,^134^ meaning RAMP evaluation of new OPV active layers is process-variable-independent.^135^

Contrast a direct and rapid assessment of the intrinsic PV performance potential of a newly invented OPV material combination with the venture of trying to find out by building devices; an OPV device engineer can fabricate a 20% power conversion efficiency device and a 2% device from the same OPV active layer and not know whether a process or fabrication issue caused poor performance or if the active layer itself simply is not viable for high power conversion efficiency. It is no wonder why the field generally focuses on optimizing materials where a high performance device result has already been demonstrated. Device-processing-independent ϕΣμ­(*t*) data are unique to and especially powerful for OPV material screening considering infinite molecular design variable space. These data can be collected in under 10 min, and when coupled with automated film fabrication, and one day with hypothesis driven synthesis-in-the-loop, the process of discovering highly valuable new semiconductor structures will be compressed from decade to days. Exciting progress toward such a future are described in the following section. Although device optimization is still required to transform a new compound or active layer composition into a functional material, we can convert the traditional problem of infinite organic semiconductor design space into an advantage by incorporating contactless screening protocols like RAMP in between the steps of semiconductor synthesis and device optimization.

## 5 Using Conductivity Spectroscopy to Discover New Materials

We have seen throughout this review that conductivity spectroscopy is an ideal tool for the noncontact evaluation of photovoltaic materials physics, and quality. In particular, there is work showing analytically how these metrics of carrier lifetime and mobility can be connected to predict device performance.^93^

The measurements of quasi-Fermi level splitting (μ_F_) and diffusion length (*L*_D_) described above already represent most of what one needs to know about a photovoltaic material. For instance, the 1-sun short-circuit current density can be roughly estimated as *J*_SC,D_ = *G*_sf_e^–*L*/*L*_D_^, where *G*_sf_ is the flux that would be absorbed by a slab of the semiconductor under study at thickness *L* and the exponential term serves to estimate the fraction of these carriers that will be collected as photocurrent. Once μ_F_ and *J*_SC,D_ are known, the fill factor (FF) may be numerically computed using the detailed balance equation for the *J*–*V* characteristics of an ideal solar cell (eq 27). Note that if external radiative efficiency (ERE) was not measured to obtain μ_F_, it may be estimated using eq 28 (or equivalently eq 25), and that *G*_s_ *q*/*L* is set equal to *J*_SC,D_. An estimated power conversion efficiency (PCE) is then given by

31 $$\mathrm{PCE}∼\frac{\mu_{F}J_{\mathrm{SC,D}}\cdot \mathrm{FF}}{100}$$

where the product μ_F_ *J*_SC,D_ must have units of mW/cm^2^ to correspond with the included solar intensity of 100 mW/cm^2^. Equivalently, the PCE may be obtained directly from the implied *J*–*V* curve calculation alluded to above.

Two assumptions are notable about this formulation, as they serve to connect the nearly field-free conductivity spectroscopy measurements we discuss throughout this manuscript with the behavior of a device in which sizable electric fields are present. The first is that the fields inside a device must remain within the linear response regime of the material. This means that the mobility obtained by conductivity spectroscopy at near-zero field is equally capable of accounting for the conductivity when the material is subject to the fields present in the device. The second is that most modern solar cells contain large field-free regions that are responsible for absorbing most of the light, making the diffusion expression we have used to approximate *J*_SC,D_ appropriate.

The development and manufacturing of novel photovoltaic materials might be greatly accelerated by broad application of robust characterization tools that can collectively predict the power conversion efficiency of devices before they are completed. This is true at every phase of PV material development, from novel material synthesis and discovery, through to process and quality control on a PV manufacturing line. The analysis above indicates that microwave or terahertz measurements combined with absorption and photoluminescence can provide exactly that.

Recently, Crovetto published a significant advance in this area,^136^ providing a new photovoltaic figure of merit which can predict PCEs given a certain collection of measured material properties including: band gap, average absorption coefficient, absorption dispersion (band tailing), the Shockley–Reade–Hall (SRH) recombination rate constant, the carrier mobility, the doping density, the static dielectric constant, and the DOS effective mass. The difficulty with this approach is that many of the proposed measurements are time-consuming to conduct, and thus not very well suited to rapid material composition screening or process control. For instance, if one wants to obtain the value of the SRH recombination rate constant, a careful sequence of spectroscopic measurements and kinetic modeling is required. However, the measurements we suggest “contain” most of this information and it is possible that machine learning approaches might be employed to side step laborious parameter extraction and enable rapid feedback based on the raw data.^137^

Efforts are already underway to employ variants of TRMC in particular for materials discovery. Most notably, Saeki has built and demonstrated an automated solar materials synthesis robot capable of producing perovskite or organic active layers and automatically characterizing them using white light (Xe lamp) flash-TRMC, photoluminescence, and absorbance spectroscopy.^138,139^

Although this research is promising, the data sets available remain rather small by machine learning standards, and it is not yet clear exactly what combination of noncontact measurements are optimal for such work.^140^ Can conductivity spectroscopy indeed be combined with other noncontact measurements to empirically predict the PCE of the ultimate devices through a machine learning approach, and avoid the need for painstaking modeling of the transient kinetics? Under what circumstances can such a simplified approach succeed? Which instruments provide the most predictive value? Below, we employ a simple but physically comprehensive simulation approach to answer these questions.

We use a 0D kinetic scheme, grounded in detailed balance,^102,104^ that both predicts the PCE of devices, and measurement results for several selected spectroscopies for a given set of input parameters. The spectroscopies simulated are external radiative efficiency (ERE, i.e., PLQY), time-resolved photoluminescence (TRPL), white-light-biased TRPL (WL-TRPL), time-resolved microwave conductivity (TRMC), white-light-biased TRMC (WL-TRMC), and steady-state microwave conductivity (SSMC).

These choices are made for several reasons. First, we posit that TRMC is much more likely to be employed in this role than OPTP given its much lower implementation and maintenance costs. We also observed in the corresponding sections of this review that light-biased TRMC and SSMC provide unique insight into the operation of a solar cell at steady state, that may be harder to gain from unbiased TRMC or TRPL. ERE is an obviously important candidate given its role in measuring the quasi-Fermi level splitting (μ_F_) that limits the open-circuit voltage (*V*_OC_), while TRPL is complementary to TRMC and *very* commonly used in the literature. We exclude any measurement that requires contacts, and take it for granted that material thickness and absorption spectra will also be measured.

A training data set is produced by stochastically varying the material properties, simulating the ultimate device PCE and spectroscopic measurements. We use this data to train a random forest machine learning model to assess the predictive power of every possible combination of these spectroscopies toward ultimate PCE.

Our results show that lifetime-based metrics are of surprisingly low value, whether they are derived from photoconductivity (TRMC) or luminescence (TRPL) measurements. Rather, the most differentially predictive information is garnered from quantitative measurements of both the photoconductivity (yield-mobility product or absolute steady-state photoconductivity) and external radiative efficiency (ERE, PLQY). Nevertheless, the best predictive power is always obtained from the full suite of tools simulated here, suggesting that the “fingerprint” approach using several orthogonal measurement techniques will always be superior where time and laboratory budget allow.

### 5.1 A Kinetic Model Bound by Detailed Balance

The simulation approach we employ in this work is designed to meet two goals: it should be computationally cheap, and simultaneously present the diverse array of physical phenomena that can impact the performance of a photovoltaic absorber material. Specifically, the scheme and parameter range are designed to explore physical situations that are likely to confound any one spectroscopic measurement that might be expected to provide a simple correlation with device PCE. For instance, photoconductivity measurements will fail to correlate with PCE in at least two situations. First, when the electron and hole mobilities in a material are dramatically different from each other, TRMC and OPTP measurements will only observe the larger of the two mobilities. This results in large photoconductivity values but poor PCE if the lower carrier mobility is below a practical collection transport length. Conversely, a situation may also arise where where the mobilities vary significantly as a function of material composition but the lower carrier mobility is still large enough to provide good PCE. One can argue that this makes ERE the indispensable tool, as ERE must approach 1 for the PCE to approach the detailed-balance limit.^104^ While this is undeniably true, it is unlikely to be sufficient by itself. It is trivial to observe that many materials exist with near unity ERE, but which exhibit extremely poor photovoltaic performance because of limited mobility. We thus choose a very general kinetic model that is notably similar to one employed extensively already for modeling hybrid halide perovskite active layers.^65,73,141–144^ These equations are solved in zero dimensions and coupled to the ideal diode equation as per the original detailed-balance limit for solar cell efficiency.^104^ This allows us to explore a wide variety of behavior in a very simple but thermodynamically rigorous format. The equations that define our model are as follows, with all variables and parameters defined in Table 1. The kinetic scheme is given by

$$\frac{dp}{dt}=G+G_{0}-\left(\gamma_{r}+\gamma_{\mathrm{nr}}\right)pn-k_{\mathrm{ex}}\left(V\right)p-\gamma_{r}\left(N_{t}-p_{t}\right)p+k_{\mathrm{dt}}p_{t}$$ 32

33 $$\frac{dn}{dt}=G+G_{0}-\left(\gamma_{r}+\gamma_{\mathrm{nr}}\right)pn-k_{\mathrm{ex}}\left(V\right)p-\gamma_{\mathrm{nr}}p_{t}n$$

$$\frac{dp_{t}}{dt}=\gamma_{r}\left(N_{t}-p_{t}\right)p-k_{\mathrm{dt}}p_{t}-\gamma_{\mathrm{nr}}p_{t}n$$ 34

in which

35 $$k_{\mathrm{ex}}\left(V\right)=\left[\frac{k_{B}T}{6q}-\left(V-V_{\mathrm{bi}}\right)\right]\frac{\mu }{L^{2}}$$

36 $$k_{\mathrm{dt}}=k_{\mathrm{dt}0}e^{-qE_{t}/k_{B}T}$$

We calculate the current density in a device from steady-state solutions of the above equations using the expressions previously derived by Shockley and Queisser,^104^ adapted to our more elaborate kinetic model:

37 $$J\left(V\right)=\left[k_{\mathrm{ex}}\left(V\right)p-\frac{G_{0}}{\mathrm{ERE}}\left(-1+e^{qV/k_{B}T}\right)\right]qL$$

38 $$\mathrm{ERE}=\frac{\gamma_{r}pn}{G+G_{0}}$$

We also require that

39 $$k_{\mathrm{ex}}\left(V\right)p\leq G$$

The parameters that we vary stochastically to investigate different families of “material” are displayed in bold in Table 1. These include the electron and hole mobilities, the radiative and nonradiative recombination rate constants, the trap density, and the doping density. We do not vary extinction coefficient, film thickness, band gap, or band edge dispersion. It is already known that these material properties are vital and can be obtained in a fully noncontact manner via spectroscopic ellipsometry or simple transmission measurements with a second mechanism of measuring thickness. Instead, we assume that all the “materials” we “measure” are optically thick, with a step-edge band gap at 1.4 eV, and focus on measurements that address the electronic quality of a material.

**Table 1:** All Parameters and Variables Required to Define Our Kinetic Model

parameter or variable	description	units	values
*p*	hole concentration	cm^–3^	–
*p*_t_	trapped hole concentration	cm^–3^	–
*n*	electron concentration	cm^–3^	–
*G*	generation rate (solar or otherwise)	cm^–3^ s^–1^	–
*G*_0_	blackbody generation rate	cm^–3^ s^–1^	–
**γ_r_**	**radiative recombination rate constant**	cm^3^ s^–1^	1 × 10^–12^ to 1 × 10^–8^
**γ_nr_**	**nonradiative recombination rate constant**	cm^3^ s^–1^	1 × 10^–12^ to 1 × 10^–8^
*k*_ex_	charge extraction/injection rate constant	s^–1^	–
***N***_**t**_	**hole trap density**	cm^–3^	1 × 10^15^ to 1 × 10^18^
***N***_**e**_	**electron doping density**	cm^–3^	1 × 10^15^ to 1 × 10^18^
*k*_dt_	detrapping rate constant	s^–1^	–
*k*_dt0_	detrapping attempt rate constant	s^–1^	1 × 10^–5^
*T*	temperature	K	300
*E*_t_	trap energy below the band edge	eV	0.1
**μ** = min(μ_e_, μ_h_)	**the lesser of the two carrier mobilities**	cm^2^ V^–1^ s^–1^	1 × 10^–3^ to 5
*E*_g_	band gap	eV	1.4
*V*_bi_	built-in potential	V	0.8*E*_g_/*q*
*L*	film thickness	cm	100 × 10^–7^
*q*	elementary charge	C	1.602 × 10^–19^
*k*_B_	Boltzmann’s constant	J K^–1^	1.38 × 10^–23^

One subtle but important simplifying assumption employed above warrants brief discussion: we allow the bimolecular radiative rate constant to vary arbitrarily for a fixed material thickness and extinction coefficient. This is not strictly physical, since the extinction coefficient is intrinsically tied to the radiative rate constant in any material by two conditions: (1) the absorption and emission of radiation must balance at equilibrium with the blackbody radiation of the surroundings, and (2) the density of electrons in the conduction band must obey Boltzmann statistics.^102^ It turns out, however, that this consideration is unimportant for ensuring that the simulation reproduces the detailed-balance efficiency limit. This is because one can make the recombination rate constants arbitrarily small, and the result is that the equilibrium carrier density becomes arbitrarily large when the equations are solved. Thus, the rate of recombination at equilibrium still balances with incoming radiation. Importantly, it is the log of the ratio between this equilibrium carrier density and that under solar illumination that determines the voltage output of the device. Moreover, although this “arbitrarily high” equilibrium carrier density might appear to violate Boltzmann statistics; it does not. This is simply equivalent to allowing the density of states to float stochastically, as is appropriate for simulating a disparate family of materials. Certainly, lower radiative recombination rate constants should result in lower extinction coefficients and thus require thicker devices to effectively absorb all above-gap solar radiation. This would in turn shift the value of mobility needed to extract charge efficiently. However, by keeping these parameters fixed in the model we are able to dramatically reduce the parameter space needed for both parameter variation and machine learning. It means that the specific machine learning model we train will never be applicable to real world materials unlike the figure of merit equation presented by Crovetto,^136^ but it does not impact the goal of this study. As noted above, we take it as a given that absorbance spectra and thickness measurements will always be employed in combination with the electronically oriented spectroscopies studied here, which should provide ample information to account for these physics in an ML model.

### 5.2 Conductivity Spectroscopy is a Vital Materials Discovery Tool

Figure 9 shows example simulations of (a) *J*–*V* curves for a variety of different hole trap densities, (b) TRMC and TRPL transients with and without white-light bias for the highest trap density *J*–*V* curve in (a). The *J*–*V* curves illustrate the reasonable approximation provided by the model of how a device would be expected to respond to limited carrier extraction efficiency. The maximum power point at which we calculated PCE is marked for the most efficient curve. The comparison of TRMC and TRPL results illustrates two important features. First, the white-light bias significantly alters both the photoconductivity and luminescence dynamics, highlighting the fact that these raw “measurements” are providing qualitatively different information (though of course they arise from the same kinetic scheme). Second, the TRMC and TRPL dynamics also appear qualitatively different from each other. As already noted in an earlier section, this is commonplace: photoconductivity measurements give a signal that is proportional to the mobile carrier concentration in the film; the TRPL amplitude is proportional to the rate of carrier recombination and thus generally is the time-derivative of the TRMC signal. Note how the PL amplitude drops to zero in this case when the photoconductivity decay flattens out at around 300 ns. The present case is an extreme example: a high density of charge selective trap states is rapidly removing the population of mobile holes from the valence band which quenches radiative recombination. Meanwhile, the electrons remain mobile in the conduction band and contribute the slowly decaying tail of the photoconductivity.

**Figure 9:**
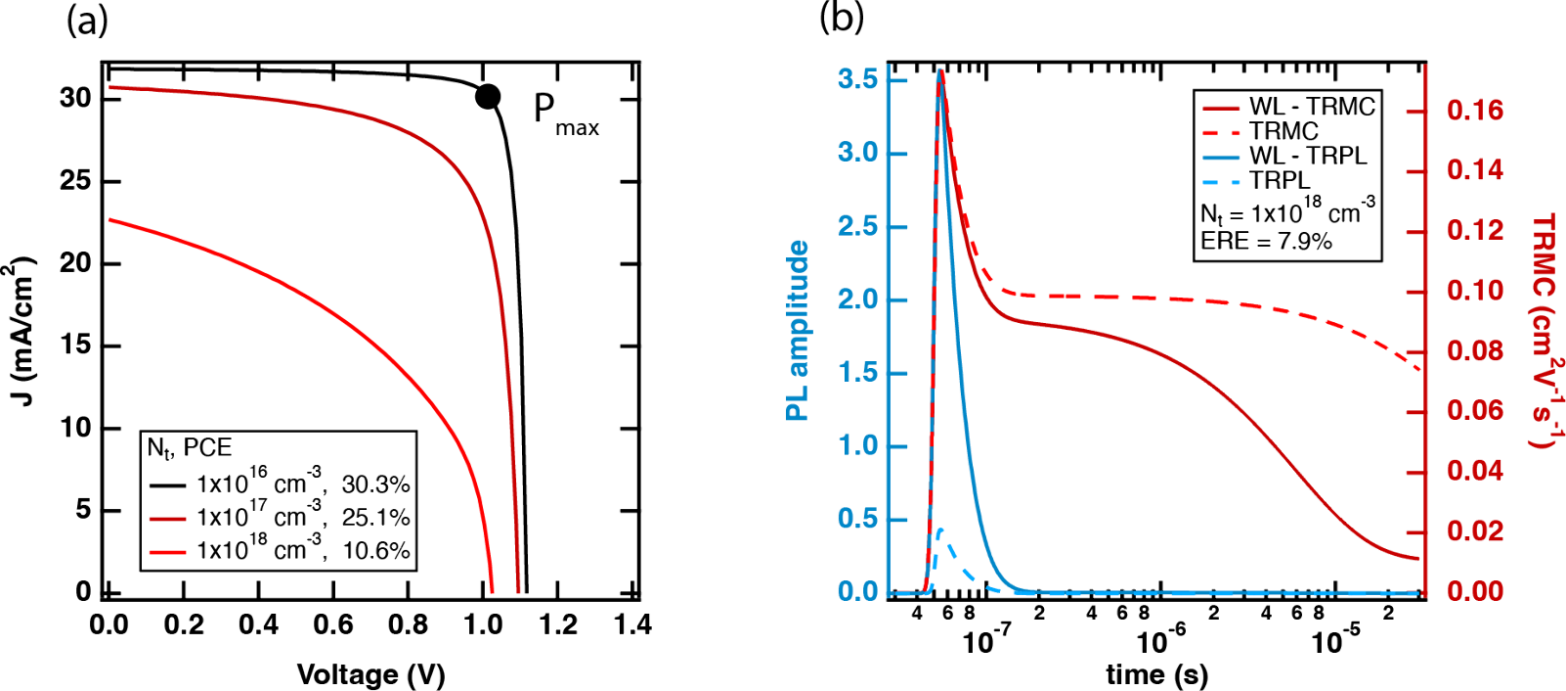
(a) Simulated *J*–*V* curves for a solar cell with varying trap density between 1 × 10^16^ and 1 × 10^18^ cm^–3^. (b) Simulated TRPL (blue, left axis) and TRMC (red, right axis) transients for the case with the most traps (1 × 10^18^ cm^–3^), showing both with (solid lines) and without (dashed lines) a 1-sun white-light bias. Other parameters: μ_e_ = μ_h_ = 0.1 cm^2^ V^–1^ s^–1^, *N*_e_ = 1 × 10^15^ cm^–3^, γ_r_ = 5.3 × 10^–11^ cm^3^ s^–1^, γ_nr_ = 1 × 10^–12^ cm^3^ s^–1^.

We used this model to generate data sets via random parameter variation over the ranges noted in Table 1, employing different limits to simulate either a materials discovery setting, where the parameters can be almost anything, or a materials optimization setting, where the baseline properties (particularly mobility and the relative radiative rate) are always quite good. We used 5000 iterations of this procedure (>500,000 solutions to the system of equations) to generate a data set suitable for machine learning. We trained a random forest ensemble model (SciKit Learn ensemble RandomForestRegressor with 1000 estimators) on the first 80% of each data set and scored its performance against the remaining 20%. Eight measurement metrics were simulated in addition to the device PCE: SSMC amplitude, ERE efficiency, TRMC amplitude (ϕΣμ), TRMC half-life (ϕΣμ τ_1/2_), TRPL half-life (PL τ_1/2_), and white-light-biased versions of each transient technique (WL-ϕΣμ, WL-ϕΣμ τ_1/2_, WL-PL τ_1/2_).

Figure 10 shows results from a combinatorial comparison of all possible combinations of measurement metrics. These grid plots show the prediction scores (*P* = *R* ^2^, color scale) of the machine learning model, trained on 80% of the data, and tested against the remaining 20%. Black indicates an empty value, where the corresponding measurement metric did not contribute to training that rows’ machine learning model. *P* = 1 corresponds to perfect correlation, and *P* = 0 corresponds to predicted values that are invariant in the independent variables (simulated measurements). Note the differing scaling of the three panels. The scale limits correspond to the minimum and maximum prediction scores. The column of numbers (*N*) on the left indicates how many measurement metrics were allowed to train the ML model in each region. There is one score value per row, and whether a given column corresponding to a specific measurement metric is colored depends on whether it contributed to training the ML model for the corresponding row.

**Figure 10:**
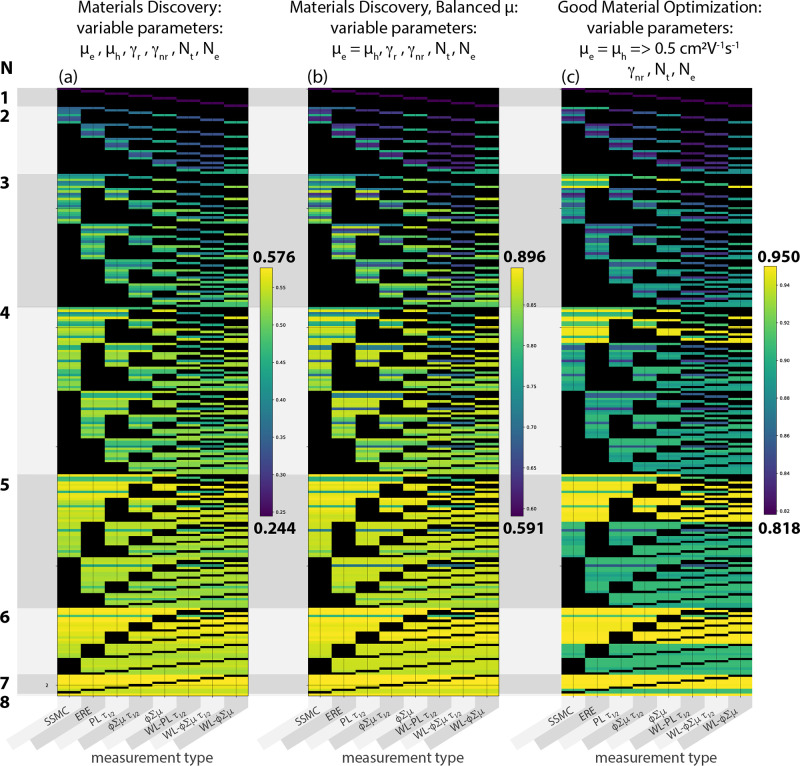
Results from a combinatorial comparison of all possible combinations of measurement metrics. These grid plots show the prediction score (*P*, color scale) of the machine learning model, trained on 80% of the data and tested against the remaining 20%. Black indicates an empty value, where the corresponding measurement metric did not contribute to training that row’s machine learning model. *P* = 1 corresponds to perfect correlation, and *P* = 0 corresponds to predicted values that are invariant in the independent variables (measurements).

The three columns (a–c) show results for three different situations corresponding to widely varying material properties similar to what might be found in a materials discovery setting (a), widely varying properties, but with all materials having balanced carrier mobilities (b), and investigation of a single near-optimal material with constant and large radiative rate constant (γ_r_ = 1 × 10^–9^ cm^3^/*s*) and large, balanced carrier mobility (0.5 cm^2^ V^–1^ s^–1^ ≥ μ_e_ = μ_h_ ≤ 5 cm^2^ V^–1^ s^–1^).

Figure 11a–c shows the mean prediction scores of random forest machine learning when only three measurement metrics are allowed. The averages are taken over all scores involving a particular measurement metric (*x* axis), allowing a comparison of how much each measurement approach is likely to contribute to predictivity. Results are shown for three different situations: widely varying material properties similar to what might be found in a materials discovery setting (a); widely varying properties, but with all materials having balanced carrier mobilities (b); and investigation of a single near-optimal material with constant and large radiative rate constant (γ_r_ = 1 × 10^–9^) and large, balanced carrier mobility (0.5 cm^2^ V^–1^ s^–1^ ≤ μ_e_ = μ_h_ ≤ 5 cm^2^ V^–1^ s^–1^). Figure 11d–f provides the correlation graphs and scores for the best three metrics under each of these conditions. Immediately we see that in all situations the white-light-biased yield mobility product (WL-ϕΣμ) provides, on average, the most individually valuable feedback to the model. Unbiased ϕΣμ is a close second, and which method comes in after that depends on the conditions. ERE becomes a particularly useful measurement when the mobility is always high (Figure 11c), as one might expect. Surprisingly, the lifetime measurements, whether derived from TRMC or TRPL, transients are some of the least useful contributors.

**Figure 11:**
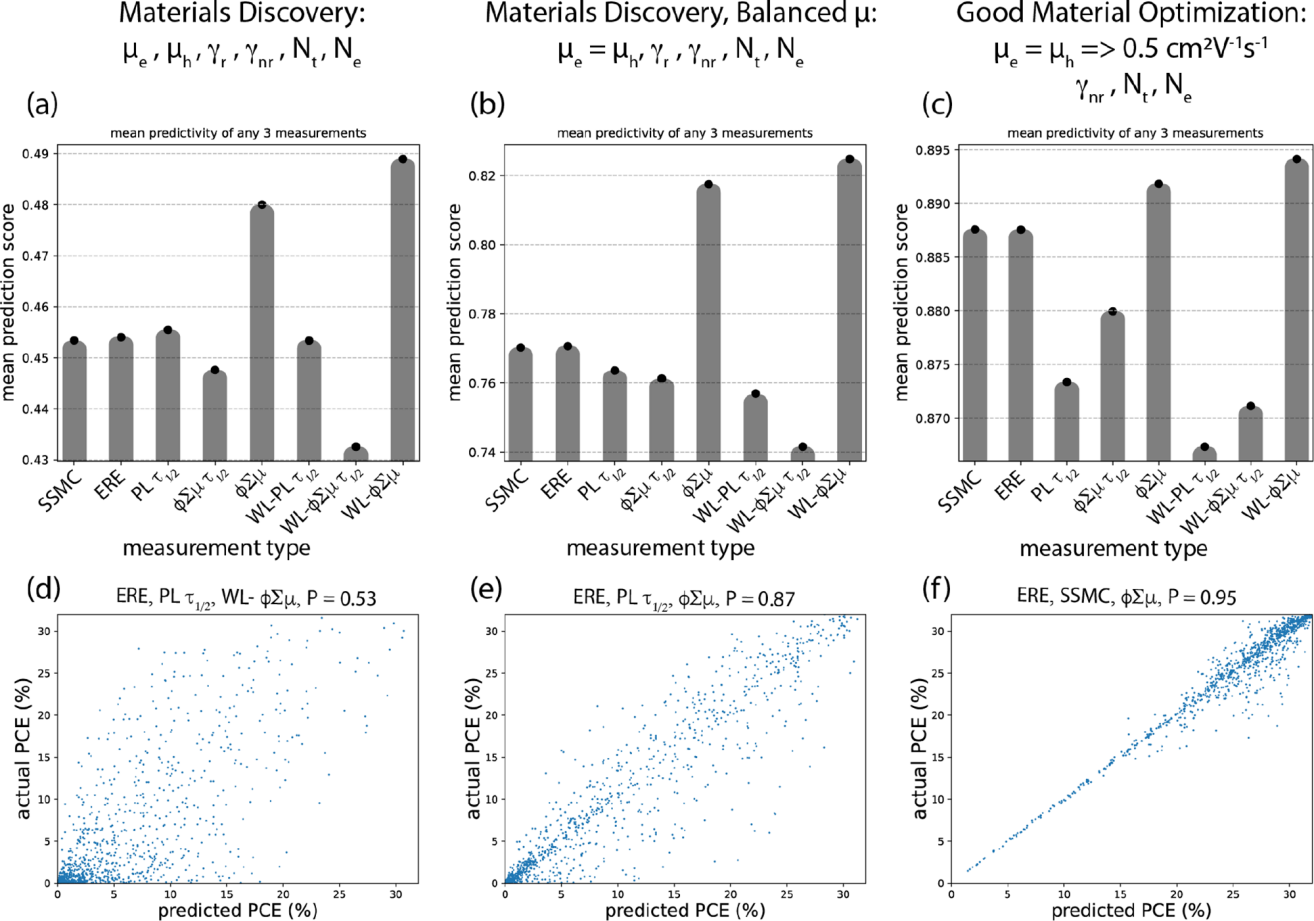
(a–c) Mean prediction scores of random forest machine learning when only three measurement metrics (simulated measurement types) are allowed. The averages are taken over all scores involving a particular measurement metric (*x* axis), allowing a comparison of how much each measurement approach is likely to contribute to predictivity. Results are shown for three ifferent situations: widely varying material properties similar to what might be found in a materials discovery setting (a); widely varying properties, but with all materials having balanced carrier mobilities (b); and investigation of a single near-optimal material with constant and large radiative rate constant (γ_r_ = 1 × 10^–9^) and large, balanced carrier mobility (0.5 cm^2^ V^–1^ s^–1^ ≤ μ_e_ = μ_h_ ≤ 5 cm^2^ V^–1^ s^–1^). (d–f) Correlation graphs and scores for the best three metrics under each of these conditions.

Another way of looking at this data set (where only three measurement types are allowed to train the model) is to examine the champion results. Which measurements contribute to the most predictive training process? Figure 11d–f gives these results. We find that for widely varying parameters, the most predictive ML model was trained using external radiative efficiency (ERE), photoluminescence half-life (PL τ_1/2_), and either white-light-biased or unbiased TRMC amplitude (WL-ϕΣμ or ϕΣμ). When better, less variable materials are simulated (Figure 11c,f), the steady-state microwave conductivity (SSMC) replaces PL τ_1/2_ in the trio.

### 5.3 Mobility Ratio is the Crucial Factor

We draw several major conclusions from this simple model. First, purely spectroscopic prediction of how well a given material could perform in a PV device is difficult, but feasible. Second, noncontact photoconductivity measurements like TRMC or OPTP are most valuable in combination with more common PL-based methods. They provide qualitatively different information about the sample than PL and while one is not “better” than the other (all individual measurements have their own prediction score weaknesses when used alone) they must be included in any high-throughput measurement suite, whether it is intended for materials discovery or process control.

The largest barrier to success is the possibility that new materials might have very unbalanced charge carrier mobilities. Note as well that even Crovetto’s proposed figure of merit derivation^136^ assumes *balanced* electron and hole mobilities. Finding a way of measuring the mobility ratio spectroscopically would dramatically improve the prospects of this approach for PV materials discovery, as illustrated by the very different maximum prediction scores in Figure 10a versus [Fig fig10]b, Figure 11a versus [Fig fig11]b, and Figure 11d versus [Fig fig11]e.

While conductivity spectroscopy cannot usually separate electron from hole contributions, there are ways to attack this problem. First, there are a variety of methods combining conductivity spectroscopy with electrically contacted devices that can provide insight, such as TOF measurements combined with microwave conductivity^128^ CELIV.^129^ Charge-modulation (or field-induced) methods^122–127,130^ may also be a viable way to do this. These are wonderful experiments, but anything that includes the construction of a device limits the high-throughput nature of fully noncontact evaluation of materials that we focus on here.

To this end, band-structure calculations can provide the ratio of effective masses for electrons and holes, and used to weight the mobility data obtained experimentally.^93,145^ It has also been shown in at least one case that very careful modeling of the fluence and wavelength-dependence of terahertz conductivity data can be used to extract electron and hole mobilities separately.^146^ However, we believe that the most promising approach for evaluating mobility ratio via noncontact experimental methods is the introduction of charge selective layers or the deliberate incorporation of traps. This has been demonstrated both for adventitious trapping and for intentionally introduced structures. For instance, it has been shown that when one carrier is trapped, it becomes possible to kinetically separate electron and hole contributions to the transient dynamics,^147,148^ and thus obtain individual electron and hole mobilities. The same thing is true when charge selective contact layers^48,144,149–151^ or chemically controlled trap-sites^152^ are introduced. While this approach adds additional experiments to the queue, and suitable contact layers or chemical dopants need to be identified for each material, it does offer the possibility of obtaining this vital mobility ratio information in a fully spectroscopic way.

Finally, the discussion above emphasizes research objectives based on discovering novel semiconductors for photovoltaics, but the same techniques, measurements, and combined approaches would be just as valuable for understanding the changes in materials over time, their stability. Degradation of properties may come from the stressful or long-term environments of operation or manufacturing. There is an obvious benefit to using device-proxy samples and conductivity spectroscopies to understand the expected operational failures in applied science, but less obvious is the value to fundamental science objectives where intrinsic and extrinsic degradation mechanisms are often an afterthought or “outside of scope” during novel semiconductor discovery. We envision another paradigm shift in the speed of functional technology development as automation, computational assistance, and conductivity spectroscopies converge toward understanding the changes in materials properties when stressed, ultimately enabling co-optimization of both performance and stability of new materials.

## 6 Conclusions

Every institution that engages in research on photovoltaic or photoelectrochemical materials *should* have a conductivity spectroscopy capability, in much the same way that they all have photoluminescence and time-resolved photoluminescence instruments. These are indispensable tools for both studying the fundamental processes of converting light into electricity in existing materials, and discovering new ones.

Photoconductivity spectroscopy directly reveals the yield-mobility and lifetime of photogenerated charges; its dark variants can measure doping density and dielectric constant, while steady-state and illumination-biased experiments reveal their behavior under solar-intensity operating conditions. All of these measurements combine to form a complete picture of the quality and/or potential efficiency of a photovoltaic absorber layer. Conductivity spectroscopy is the *most* predictive measurement we tested for evaluating the photovoltaic potential of an arbitrary sample in our simulated ML-driven high-throughput lab.

The past decade of research in this area has seen these tools grow into their full potential as a spectroscopic method of predicting solar cell efficiency from a minimal material sample, such as a powder or a poor-quality film. We recommend that the practice of combining dark, steady-state, and time-resolved data to predict quasi-Fermi level splitting and diffusion length in candidate materials be expanded, as right now these very useful analyses remain relatively rare. We note that if the method highlighted in this review for calculating the intrinsic carrier density from a calculated effective mass proves to be sufficiently accurate, and methods of obtaining the mobility ratio are employed, then conductivity spectroscopy can become a stand-alone tool for predicting limiting solar cell efficiencies spectroscopically (combined with extinction coefficient measurements). However, it is likely that combining conductivity spectroscopy with time-resolved photoluminescence, external radiative efficiency, and structural methods that can give the characteristic grain size will always provide greater predictive power toward ultimate photovoltaic efficiency.

The fact that Warman and Weber never collaborated to discover the photoconductivity of MAPbI_3_ was an enormous missed opportunity, which we must avoid repeating. As such, we urge the photovoltaic research community to adopt conductivity spectroscopy more broadly, particularly for materials discovery, composition optimization, and manufacturing process control in high-throughput automated environments.

## Supplementary Material



## Notes

Supplementary Python modules giving the code needed to reproduce many of the figures in the text and the Igor code used to calculate our kinetic model and compute detailed-balance solar cells (all cleaned and commented in detail to make them maximally useful) (PDF)
