# Supplementary material for: Conductivity Spectroscopy for Investigation and Discovery of Photovoltaic Materials
Source: Chem Rev. 2026 May 19;126(11):6283–310. doi: 10.1021/acs.chemrev.5c00986 (PMC13261790; doi:10.1021/acs.chemrev.5c00986)
Supplement: Supplementary file 1 [file cr5c00986_si_001.pdf]

Supporting Information: Conductivity Spectroscopy for Investigation and Discovery of Photovoltaic Materials

Obadiah G. Reid*,†,‡ and Bryon W. Larson†

†*Chemistry and Nanoscience Center, National Laboratory of the Rockies, Golden CO, 80401, USA*

‡*Renewable and Sustainable Energy Institute, University of Colorado Boulder, Boulder CO, 80309, USA*

E-mail: obadiah.reid@nlr.gov

Contents

1 Where to find the code **2**

2 Frozen Python Code: **2**

2.1 classical_charge_motion.py 2

2.2 transport_kmc.py 8

2.3 complex_conductivity_analytic.py 25

3 Frozen Igor Code: **35**

3.1 ODE_Equations.ipf 35

3.2 ODE_Solver.ipf 51

3.3 ODE_Image_Fit.ipf 51

1 Where to find the code

Live copies of the code can be found on a public GitHub repository created to support this article: https://github.com/obadiahreid/Conductivity-Spectroscopy-Code

2 Frozen Python Code:

2.1 classical_charge_motion.py

```
import numpy as np
from scipy.integrate import solve_ivp
import os
import matplotlib.pyplot as plt
enclosing_folder = os.path.dirname(os.path.abspath(__file__))

def sci_notation_to_str(number, precision=1):
    """
    Convert a number to a string in the format 'number x 10^exponent' with specified precision.
    Example: sci_notation_to_str(0.0001234, 2) -> '1.23 x 10^-4'
    """
    if number == 0:
        return f"0x10_0"
    exponent = int(np.floor(np.log10(abs(number))))
    mantissa = number / (10 ** exponent)
    return f"{mantissa:.{precision}f}x10_{exponent}"

def sci_notation_to_str_latex(number, precision=1):
    """
    Convert a number to a string in the format 'number x 10^exponent' with specified precision.
    Example: sci_notation_to_str(0.0001234, 2) -> '1.23 x 10^-4'
    """
    if number == 0:
        return f"0.0"
    exponent = int(np.floor(np.log10(abs(number))))
    mantissa = number / (10 ** exponent)
    return f"{mantissa:.{precision}f}x10$^{{{exponent}}}$"

def electron_motion_damped(t, y, E0, omega, e, m, tau, k):
    """Equation of motion for an electron with damping and restoring force,
    subject to an oscillating electric field.
    parameters:
        t = time (s)
        y = [x, v] where x is position and v is velocity
        E0 = electric field amplitude (V/m)
        omega = field angular frequency (rad/s)
        e = electron charge (C)
        m = electron mass (kg)
        tau = damping time constant (s)
        k = spring constant (N/m)
```

```python
    returns:
        [dx/dt, dv/dt]
    """
    x, v = y
    dxdt = v
    dvdt = (-m/tau * v - k * x - e * E0 * np.cos(omega * t)) / m
    return [dxdt, dvdt]

# Example parameters
E0 = 1.0       # Electric field amplitude (V/m)
omega = 1e10*2*np.pi    # Angular frequency (rad/s)
e = 1.602e-19 # Electron charge (C)
m = 9.109e-31 # Electron mass (kg)
tau = 1e-12#1e-9 # Damping time constant (s)
#gamma = m/tau #1e-28 # Damping coefficient (kg/s)
k = 0#1e-8 # Spring constant (N/m)

# Initial conditions: x(0) = 0, v(0) = 0
y0_electron = [0, 0]
t_span_electron = (0, 100e-12)
t_eval_electron = np.linspace(*t_span_electron, 200)

def lock_in_detection(input_signal, t_eval_electron, omega):
    """
    Lock-in detection algorithm to define in-phase (cos) and quadrature (sin) channels.
    Parameters:
    input_signal: The measured signal (e.g., velocity of the electron) as a function of time.
    t_eval_electron: The time points corresponding to the input_signal.
    omega: The angular frequency of the reference signal (drive frequency).
    Returns:
    X: In-phase component (cosine reference)
    Y: Quadrature component (sine reference)
    """
    N=int(round((len(t_eval_electron)-1)/2))
    ref_cos = np.cos(t_eval_electron*omega)
    ref_sin = np.sin(t_eval_electron*omega)

    # Mix input with reference
    X = -np.mean(input_signal * ref_cos)*2 # multiplied by 2 because the mean of the product of two sinusoids over one period is 0.5
    Y = -np.mean(input_signal * ref_sin)*2 # multiplied by 2 because the mean of the product of two sinusoids over one period is 0.5

    return X, Y

# Example usage:
# input_signal = ... (your measured signal)
# reference_signal = ... (your reference signal, same length as input_signal)
# sampling_rate = ... (Hz)
# X, Y = lock_in_detection(input_signal, reference_signal, sampling_rate)
def compute_lock_in_values(sol_electron, omega):
    """Compute lock-in values X and Y for the electron solution.
    Parameters:
    sol_electron: The solution object returned by solve_ivp for the electron motion.
    omega: The angular frequency of the reference signal (drive frequency).
    Returns:
    X: In-phase component (cosine reference)
    Y: Quadrature component (sine reference)"""
```

```python
    X, Y = lock_in_detection(
        sol_electron.y[1],
        sol_electron.t,
        omega
    )
    # R = np.sqrt(X**2 + Y**2)
    # theta = np.arctan2(Y, X)
    # real_part = R * np.cos(theta)
    # imag_part = R * np.sin(theta)
    return X, Y

#X, Y = compute_lock_in_values(sol_electron, omega)

# print(f"X (in-phase): {X}")
# print(f"Y (quadrature): {Y}")



def sweep_frequency_and_plot(w0, w1, num_points=10):
    """Sweep the drive frequency from w0 to w1, compute lock-in values, and plot the results.
    Parameters:
        w0: Starting angular frequency (rad/s)
        w1: Ending angular frequency (rad/s)
        num_points: Number of frequency points to evaluate
    Returns:
        None (plots the results)"""
    omegas = np.logspace(np.log10(w0), np.log10(w1), num=num_points)
    X_vals = []
    Y_vals = []
    #print(omegas)
    for omega in omegas:
        t_span_electron = (0, 10/(omega/2/np.pi))
        t_eval_electron = np.linspace(*t_span_electron, 1000)
        sol = solve_ivp(
            electron_motion_damped,
            t_span_electron,
            y0_electron,
            t_eval=t_eval_electron,
            args=(E0, omega, e, m, tau, k)
        )
        X, Y = compute_lock_in_values(sol, omega)
        X_vals.append(X)
        Y_vals.append(Y)
    #x_vals *=
    fig = plt.figure(figsize=(4, 4))  # Set overall figure size to 4x4 inches
    # Make the axes fill the entire figure (plot area is 4x4)
    ax = fig.add_axes([0.25, 0.2, 0.65, 0.65])
    ax.plot(omegas, X_vals, label='$\mu^{{\prime}}$ (in-phase)')
    ax.plot(omegas, Y_vals, label='$\mu^{{\prime\prime}}$ (out-of-phase)')
    ax.set_xlabel('Drive Frequency (rad/s)')
    ax.set_xscale('log')
    ax.set_ylabel('mobility (m$^2$/V/s)')
    ax.set_title('Mobility vs Drive Frequency')
    ax.legend(loc='lower left', framealpha=0.75)

    ax.set_xlim(omegas[0], omegas[-1])
```

```
    # Increase axes and tick line width
    lw = 1.5
    ax.spines['top'].set_linewidth(lw)
    ax.spines['right'].set_linewidth(lw)
    ax.spines['bottom'].set_linewidth(lw)
    ax.spines['left'].set_linewidth(lw)
    ax.tick_params(width=lw, length=6)
    #plt.tight_layout(pad=1)
    eps_path = os.path.join(enclosing_folder, "electron_mobility_tau_{}_k_{}.eps".format(sci_notation_to_str(tau), sci_notation_to_str(k)))
    plt.savefig(eps_path, format='eps')
    print(f"Figure saved to {eps_path}")
    plt.show()

# Example usage:
#sweep_frequency_and_plot(1e9, 10000e9, num_points=100)

def dipole_motion_damped(t, y, E0, omega, mu, I, tau, k):
    """
    Equation of motion for an electric dipole with damping and restoring force,
    subject to an oscillating electric field:
    I * d2p/dt2 + I/tau * dp/dt + k * p = mu*sin(p) * E0 * cos(omega * t)
    where:
        p = angle relative to the applied field
        I = effective angular moment of inertia
        tau = damping time constant
        k = restoring force constant
        mu = dipole moment
        E0 = electric field amplitude
        omega = field angular frequency
    y[0] = p, y[1] = dp/dt
    """
    p, dpdt = y
    d_pdt = dpdt
    d_dpdt = (-I/tau * dpdt - k * (p-np.pi/4) + mu*np.sin(p)* E0 * np.cos(omega * t)) / I
    return [d_pdt, d_dpdt]

# Example parameters for dipole motion
E0_dipole = 1.0      # Electric field amplitude (V/m)
omega_dipole = 1e10*2*np.pi   # Angular frequency (rad/s)
mu = 3.336e-30*10           # Dipole moment (C*m)
I = 1e-45          # Moment of inertia (kg*m^2)
tau = 1e-12#1e-9           # Damping time constant (s)
k_dipole = 1e-22#1e-8      # Restoring force constant (N*m/rad)

    # Initial conditions: p(0) = 0, dp/dt(0) = 0
y0_dipole = [np.pi/4, 0]
t_span_dipole = (0, 10/omega_dipole*2*np.pi)
t_eval_dipole = np.linspace(*t_span_dipole, 200)


#X, Y = compute_lock_in_values(sol_dipole, omega_dipole)
def sweep_frequency_and_plot_dipole(w0, w1, num_points=10):
    """Sweep the drive frequency for the dipole motion, compute lock-in values, and plot the results.
    Parameters:
        w0: Starting angular frequency (rad/s)
        w1: Ending angular frequency (rad/s)
```

```
        num_points: Number of frequency points to evaluate
    Returns:
        None (plots the results)"""

    omegas = np.logspace(np.log10(w0), np.log10(w1), num=num_points)
    X_vals = []
    Y_vals = []
    for omega in omegas:
        t_span_dipole = (0, 10/(omega/2/np.pi))
        t_eval_dipole = np.linspace(*t_span_dipole, 100)
        sol = solve_ivp(
            dipole_motion_damped,
            t_span_dipole,
            y0_dipole,
            t_eval=t_eval_dipole,
            args=(E0_dipole, omega, mu, I, tau, k_dipole)
        )
        X, Y = compute_lock_in_values(sol, omega)
        X_vals.append(-X)
        Y_vals.append(-Y)

    fig = plt.figure(figsize=(4, 4))  # Set overall figure size to 4x4 inches
    # Make the axes fill the entire figure (plot area is 4x4)
    ax = fig.add_axes([0.25, 0.2, 0.65, 0.65])
    ax.plot(omegas, X_vals, label='Real (in-phase)')
    ax.plot(omegas, Y_vals, label='Imaginary (out-of-phase)')
    ax.set_xlabel('Drive Frequency (rad/s)')
    ax.set_xscale('log')
    ax.set_ylabel('mobility (rad$^2$/V/s)')
    ax.set_title('Dipole Mobility vs Drive Frequency')
    ax.legend()
    ax.set_xlim(omegas[0], omegas[-1])
    # Increase axes and tick line width
    lw = 1.5
    ax.spines['top'].set_linewidth(lw)
    ax.spines['right'].set_linewidth(lw)
    ax.spines['bottom'].set_linewidth(lw)
    ax.spines['left'].set_linewidth(lw)
    ax.tick_params(width=lw, length=6)
    #plt.tight_layout(pad=1)
    eps_path = os.path.join(enclosing_folder, "dipole_mobility_tau_{}_k_{}.eps".format(sci_notation_to_str(tau), sci_notation_to_str(k_dipole)))
    plt.savefig(eps_path, format='eps')
    print(f"Figure saved to {eps_path}")
    plt.show()

sweep_frequency_and_plot_dipole(1e9, 10000e9, num_points=100)

# Save the last pyplot figure as an EPS file in the enclosing folder

tau_vals = [1e-12, 1e-9, 1e-6]
k_vals = [1e-8, 1e-10, 0]
w0 = 1e9
w1 = 10000e9
points = 100

# Sweep over all combinations of tau_vals and k_vals
```

```python
def sweep_all_tau_k(tau_vals, k_vals, w0, w1, points):
    """Sweep over all combinations of tau_vals and k_vals, compute lock-in values, and plot the results.
    Parameters:
        tau_vals: List of tau values to sweep
        k_vals: List of k values to sweep
        w0: Starting angular frequency (rad/s)
        w1: Ending angular frequency (rad/s)
        points: Number of frequency points to evaluate
    Returns:
        None (plots the results)"""

    for tau_val in tau_vals:
        for k_val in k_vals:
            global tau, k
            tau = tau_val
            k = k_val
            print(f"Running sweep for tau={tau}, k={k}")
            sweep_frequency_and_plot(w0, w1, num_points=points)

sweep_all_tau_k(tau_vals, k_vals, w0, w1, points)

# Solve electron_motion_damped for tau=1e6, k=0, omega=1e10
tau_plot = 1e-6
k_plot = 0
omega_plot = 1e10*2*np.pi

def plot_electron_velocity_and_field(E0, omega_plot, tau_plot, k_plot, y0_electron):
    """Plot the electron velocity and electric field as a function of time for given parameters.
    Parameters:
        E0: Electric field amplitude (V/m)
        omega_plot: Angular frequency of the electric field (rad/s)
        tau_plot: Damping time constant (s)
        k_plot: Spring constant (N/m)
        y0_electron: Initial conditions for the electron motion [x(0), v(0)]
    Returns:
        None (plots the results)"""

    t_span_plot = (0, 4/omega_plot*2*np.pi)
    t_eval_plot = np.linspace(*t_span_plot, 500)
    sol_plot = solve_ivp(
        electron_motion_damped,
        t_span_plot,
        y0_electron,
        t_eval=t_eval_plot,
        args=(E0, omega_plot, e, m, tau_plot, k_plot)
    )

    fig = plt.figure(figsize=(4, 4))  # Set overall figure size to 4x4 inches
    # Make the axes fill the entire figure (plot area is 4x4)
    ax = fig.add_axes([0.25, 0.2, 0.65, 0.65])
    ax.plot(sol_plot.t, sol_plot.y[1], label='v(t) electron')
    E_t_plot = -E0 * np.cos(omega_plot * sol_plot.t) * np.max(sol_plot.y[1])  # Scale for visibility
    ax.plot(sol_plot.t, E_t_plot, color='black', linewidth=1, label='E(t) field')
    ax.axhline(0, color='gray', linestyle='--', linewidth=2)
    ax.set_xlabel('t (s)', fontsize=12)
    ax.set_ylabel('Velocity / Electric Field', fontsize=12)
```

```
    ax.set_title(f'Velocity ($\\tau$={sci_notation_to_str_latex(tau_plot)}, k={sci_notation_to_str_latex(k_plot)},
    ↪  $\\omega$={sci_notation_to_str_latex(omega_plot)})', fontsize=10, fontweight='bold')
    ax.legend(loc='upper right', framealpha=0.95, prop={'weight': 'bold'})
    ax.set_xlim(sol_plot.t[0], sol_plot.t[-1])
    # Increase axes and tick line width
    lw = 1.5
    ax.spines['top'].set_linewidth(lw)
    ax.spines['right'].set_linewidth(lw)
    ax.spines['bottom'].set_linewidth(lw)
    ax.spines['left'].set_linewidth(lw)
    ax.tick_params(width=lw, length=6)
    #plt.tight_layout(pad=1)
    eps_path = os.path.join(enclosing_folder, "electron_velocity_field_tau_{}_k_{}.eps".format(sci_notation_to_str(tau_plot),
    ↪  sci_notation_to_str(k_plot)))
    plt.savefig(eps_path, format='eps')
    print(f"Figure saved to {eps_path}")
    plt.show()



plot_electron_velocity_and_field(E0, omega_plot, tau_plot, k_plot, y0_electron)

tau_plot = 1e-12
k_plot = 0
omega_plot = 1e10*2*np.pi

plot_electron_velocity_and_field(E0, omega_plot, tau_plot, k_plot, y0_electron)

tau_plot = 1e-12
k_plot = 1e-8
omega_plot = 1e10*2*np.pi

plot_electron_velocity_and_field(E0, omega_plot, tau_plot, k_plot, y0_electron)
```

2.2 transport_kmc.py

```
#!/usr/bin/env python3
# -*- coding: utf-8 -*-
"""
Created on Jan 1 2025
@author: oreid

A general 1D kMC code based on the Drude model to calculate charge carrier mobility and complex mobility from velocity autocorrelation functions.
"""

import numpy as np
import matplotlib.pyplot as plt
import time as time
#numba is awesome. It can compile python code to C speed.
# it is vital for this code to run fast enough to be useful.
from numba import jit
from numba.experimental import jitclass
import numba
import gc
```

```python
import os as os
from matplotlib.ticker import ScalarFormatter



# Calculate the velocity autocorrelation function

@jit(nopython=True)
def calcVelocityAutorCorrelationLog(v, t, Ct_span = 0.02e-9, dt_absmin = 1e-15, inc = 1.01, lag = 1):
    """Calculate the velocity autocorrelation function.
    Parameters
    ----------
    v : 1D array
        The velocity points of the trajectory (m/s).
    t : 1D array
        The time points of the trajectory (s).
    Ct_span : float
        The maximum time span to calculate the autocorrelation function (s).
    dt_absmin : float
        The minimum time step to use for the even time grid (s)."""

    # figure out how many points to use in the autocorrelation function
    N = round(len(v))

    dt_min = t[N-1]-t[0]
    for i in range(N-1):
        dt = t[i+1]-t[i]
        if dt < dt_min:
            dt_min = dt
        if dt_min < dt_absmin:
            dt_min = dt_absmin

    #print (dt_min)

    t_span = t[N-1]-t[0]
    # print(dt_min)
    # print(t_span)
    #figure out how many points to use in the autocorrelation function
    t_cor = Ct_span
    #inc = 1.01
    order_tmin = np.log(dt_min)
    order_tspan = np.log(t_cor)
    steps = abs(order_tspan - order_tmin)/np.log(inc) + 1
    CN = int(steps)
    #print(CN)
    #initialize the autocorrelation function arrays
    Ct = np.zeros(CN, dtype=np.float64)
    Ct[0] = 0
    t_total = np.float64(0.0)
    for i in range(1, CN):
        Ct[i] = dt_min*inc**i

    C = np.copy(Ct)
    Ci = np.copy(Ct)
    C[:]=0
    Ci[:]=0
```

```python
    N_intervals = np.floor(t_span/(t_cor*lag))
    if N_intervals > N:
        N_intervals = N
    interval_size= np.floor(N/N_intervals)

    # print(Ct)
    # calculate the autocorrelation function
    for i in range(N_intervals):
        ii = int(i*interval_size)
        if t[ii] > t_span-t_cor:
            #print(i)
            #print(t[i])
            break
        vi = v[ii]
        #print(4)

        k = 1
        for j in range(0, CN):

            if Ct[j] < t[ii+k]-t[ii]:
                k += 0
            else:
                k += 1


            ti_weight = t[ii+1]-t[ii]

            vj = v[ii+k-1]
            C[j] += vi*vj#*ti_weight

        t_total += ti_weight
        # Weighting by the time of each starting point is needed to correct for non-thermal VACF results when there are elastic scattering events
        # in the system.



    #C[:] /= t_total#np.float64((i-1)) # normalize the average for the autocorrelation function
    C[:] /= np.float64((i-1))
    # notice that the autocorrelation function is not normalized to 1 at t=0
    # this is because we are interested in the absolute value of the autocorrelation function
    # for calculating the complex mobility, not the normalized value. The peak value is
    # equal to the mean squared velocity.
    return C, Ct

# Calculate the complex mobility from the velocity autocorrelation function
@jit
def calcComplexMobility(C, t, w, MSD = False):
    """Calculate the complex mobility from the velocity autocorrelation function.
    Parameters
    ----------
    C : 1D array
        The velocity autocorrelation function (m^2/s^2).
    t : 1D array
        The time points of the autocorrelation function (s).
    w : float
        The angular frequency to calculate the complex mobility at (rad/s)."""
```

```python
    N = len(C)
    q = 1.602e-19
    kB = 1.381e-23
    T = 300
    mu = 0 + 0j
    # use the Kubo formula to calculate the complex mobility
    for i in range(N-1):
        mu += (q/(kB*T))*(C[i]+C[i+1])/2*np.exp(1j*w*t[i])*(t[i+1]-t[i])

    if MSD:
        mu *= w**2/2
    return mu # in m^2/Vs. (but ultimatly determined by the input units of C and t)

# Calculate the complex mobility over a range of frequencies
@jit
def calcComplexMobilityRange(C, t, wmin, wmax, Nw, MSD = False):
    """Calculate the complex mobility over a range of frequencies.
    Parameters
    ----------
    C : 1D array
        The velocity autocorrelation function (m^2/s^2).
    t : 1D array
        The time points of the autocorrelation function (s).
    wmin : float
        The minimum angular frequency to calculate the complex mobility at (rad/s).
    wmax : float
        The maximum angular frequency to calculate the complex mobility at (rad/s).
    Nw : int
        The number of frequency points to calculate the complex mobility at."""

    # use log spacing for the frequency points
    wlist = np.logspace(np.log10(wmin), np.log10(wmax), Nw)
    mulist = np.zeros(Nw, dtype=np.complex_)
    for i in range(Nw):
        w = wlist[i]
        mu = calcComplexMobility(C, t, w, MSD=MSD)
        mulist[i] = mu
    return wlist, mulist



# Begin code for drude model KMC

#define some helper functions for the drude model
@jit
def expRates(tau, dt):
    """
    Calculate an exponential distribution of rates for a given time constant (tau) and resolution (dt).
    Useful for generating scattering time distributions.

    Parameters
    ----------
    tau : float
        The time constant for the exponential decay.
    dt : float
        The time resolution for the distribution.
```

```
    Returns
    -------
    rates : 1D array
        The calculated rate (or probability) of transitions.
    time : 1D array
        The time points corresponding to the decay rate/probability.
    """
    N = int(tau/dt*10)
    rates = np.zeros(N, dtype=np.float64)
    time = np.linspace(0, N*dt, N)

    for i in range(N):
        rates[i] = np.exp(-time[i] / tau)
    return rates, time

@jit
def calcGaussRates(sigma, dv):
    """
    Calculate a Gaussian distribution of rates for a given standard deviation (sigma) and resolution (dv).
    Useful for generating velocity distributions. Or any gaussian probability distribution of a property.
    Parameters
    ----------
    sigma : float
        The standard deviation of the Gaussian distribution.
    dv : float
        The velocity resolution for the distribution.

    Returns
    -------
    rates : 1D array
        The calculated rate (or probability) of transitions.
    value : 1D array
        The velocity points corresponding to the rate/probability.
    """
    N = int(sigma/dv*10)
    rates = np.zeros((N,), dtype=np.float64)
    value = np.linspace(-N/2*dv, N/2*dv, N)
    #print(value[0], value[-1])
    mid = N/2
    for i in range(N):
        rates[i] = 1/np.sqrt(2 * np.pi * sigma**2) * np.exp(-0.5 * ((i-mid+1/2) * dv / sigma)**2)

    #print("the average velocity is: {}".format(np.mean(rates*value)))
    return rates, value

@jit
def jitZeros(shape):
    """
    A helper function to create a numpy array of zeros with a specified shape and float64 type.
    This is wrapped in a jit function to help numba determine the type more easily.
    Parameters
    ----------
    shape : tuple
        The shape of the desired array.
```

```
    Returns
    -------
    arr : 1D array
        A numpy array of zeros with the specified shape and float64 type.
    """
    arr = np.zeros(shape, dtype=np.float64)
    return arr

@jit
def chooseRate(rates):
        """
        Make a MC choice of which transition to execute. probability is controlled
        by the relative rate constant of each transition.
        Parameters
        ----------
        rates : 1D array of floats
            a list of all the possible rates for the current state

        Returns
        -------
        i : int
            the index of the transition/rate that has been chosen from the rate list
        rn : float
            a random number between (0,1] that was used to choose that transition/rate

        """
        # add up all the rates
        R = np.sum(rates)
        # get a random number on the interval (0, 1]
        # by default np.random.rand() returns a number on the interval [0,1)
        rn = (np.random.rand()-1)*(-1)

        # initalize a counter to sum up the rates
        rateSum = 0

        #iterate through the rates building up the value of rateSum
        for i in range(len(rates)):
            rateSum+= rates[i]
            # if the rn x total rate is less than the rate sum, choose the
            # transition that was just added to the sum.
            if(R*rn < rateSum):
                break

        return int(i), float(rn)

#specify the types used in the drudeWalker jitclass
spec = [
    ('dt', numba.float64),
    ('mass', numba.float64),
    ('tau', numba.float64),
    ('L', numba.float64),
    ('elastic', numba.boolean),
    ('R', numba.float64),
    ('v', numba.float64),
    ('x', numba.float64),
    ('t', numba.float64),
```

```
    ('t_free', numba.float64),
    ('kB', numba.float64),
    ('T', numba.float64),
    ('sigma', numba.float64),
    ('vDist', numba.float64[:]),
    ('vDist_v', numba.float64[:]),
    ('tScatterDist', numba.float64[:]),
    ('tScatterDist_t', numba.float64[:]),
    ('vtraj', numba.float64[:]),
    ('xtraj', numba.float64[:]),
    ('ttraj', numba.float64[:]),
    ('lfree', numba.float64),
    ('alpha', numba.float64),
    ]

#drude model walker class. A class structure is used as it is a natural way to keep track of internal state
@jitclass(spec)
class drudeWalker:
    """
    A class to simulate a single particle undergoing a Drude model random walk in 1D
    with scattering events that randomize the velocity according to a Gaussian distribution.
    The particle is confined to a box of length L with reflecting boundaries."""

    def __init__(self, dt = 1e-15, mass = 9.1e-31, tau = 100e-15, L=100e-9, elastic = False, R=1.0):
        """Initialize the walker with the given parameters.
        Parameters
        ----------
        dt : float
            The time step for the simulation (s). Just a starting point. timestep is variable in n-fold way kMC.
        mass : float
            The mass of the particle (kg).
        tau : float
            The mean time between scattering events (s).
        L : float
            The length of the box in which the particle is confined (m).
        elastic : bool
            If True, the particle reflects elastically off the walls. If False, it reflects inelastically.
        R : float
            The reflection coefficient at particle boundaries.
        Returns
        -------
        None
        """
        self.dt = dt
        self.mass = mass
        self.L = L
        self.elastic = elastic
        self.R=R
        self.v = 0.0
        self.x = 0.0
        self.t = 0.0
        self.t_free = 0
        self.kB = 1.381e-23
        self.T = 300
        self.sigma = np.sqrt(self.kB*self.T/self.mass) # 1/2kT for each dimension of motion. In this case its 1D. Nope. Gotta find that factor of 2
        ↪  elsewhere!
```

```
        self.vDist, self.vDist_v = calcGaussRates(self.sigma, self.sigma/10)
        self.tScatterDist, self.tScatterDist_t = expRates(tau, dt)
        # this is a trick that helps specify type in a jitclass.
        # wrapping np.zeros in a jit function (above) allows numba to determine the type more easily.
        self.vtraj = jitZeros((5,))
        self.xtraj = jitZeros((5,))
        self.ttraj = jitZeros((5,))
        self.lfree = np.sqrt(self.kB*self.T/self.mass)*tau # mean free path
        self.alpha = self.L/self.lfree # alpha.
        #print('the ratio of mean-free path to particle size is:{}'.format(self.alpha))


    def step(self):
        """Perform a single step of the random walk.
        The particle moves with its current velocity for a time drawn from the scattering time distribution,
        then its velocity is randomized according to the velocity distribution at a scattering site.
        If the particle hits the boundary of the box, it reflects off the wall.
        Returns
        -------
        x : float
            The new position of the particle (m).
        v : float
            The new velocity of the particle (m/s).
        t : float
            The new time (s)."""
        i, rn = chooseRate(self.tScatterDist)
        dt = self.tScatterDist_t[i]
        self.t_free = self.t+ dt
        vi = self.v
        j, rn = chooseRate(self.vDist)
        vj = self.vDist_v[j]
        dx = vi*dt
        xi = self.x
        xj = self.x + dx
        rfl = np.zeros((2,))
        rfl[0] = self.R
        rfl[1] = 1.0 - self.R
        rfi, rn = chooseRate(rfl)

        if xj > self.L/2:
            if(rfi==1):
                self.x = xj % (self.L/2)
                self.v = vj



            else:
                xt = abs(self.L/2-xi) # distance traveled to the wall
                dt = xt/abs(vi) # time taken to reach the wall
                self.x = self.L/2
                if self.elastic:
                    self.v = -vi # elastic backscattering
                else:
                    self.v = -abs(vj) # inelastic backscattering

        elif xj< -self.L/2:
            if(rfi==1):
```

```
                self.x = xj % (-self.L/2)
                self.v = vj

            else:
                xt = abs(-self.L/2-xi) # distance traveled to the wall
                dt = xt/abs(vi) # time taken to reach the wall
                self.x = -self.L/2
                if self.elastic:
                    self.v = -vi # elastic backscattering
                else:
                    self.v = abs(vj) # inelastic backscattering
        else:

            self.x += vi*dt
            # only scatter if the last scattering interval has passed.
            if self.t_free <= self.t+dt:
                self.v = vj
            else:
                self.v = vi




        self.t += dt
        return self.x, self.v, self.t

    def walk(self, N=1000):
        """Perform N steps of the random walk.
        Parameters
        ----------
        N : int
            The number of steps to perform.
            Returns
            -------
            xtraj : 1D array
                The trajectory of the particle's position (m).
            vtraj : 1D array
                The trajectory of the particle's velocity (m/s).
            ttraj : 1D array
                The trajectory of the time (s).
        """
        self.vtraj = jitZeros((N,))
        self.xtraj = jitZeros((N,))
        self.ttraj = jitZeros((N,))
        for i in range(N):
            self.step()
            self.vtraj[i] = self.v
            self.xtraj[i] = self.x
            self.ttraj[i] = self.t
        return self.xtraj, self.vtraj, self.ttraj

#End Drude model kMC code

# Begin test functions for the drude walker

def testDrudeWalkerN(NWalkers=1000, NSteps=10000):
    """Run the drude walker for NWalkers particles, each for NSteps steps.
```

```
    Parameters. Use parallelization to speed up the calculation.
    ----------
    NWalkers : int
        The number of walkers to simulate.
    NSteps : int
        The number of steps to perform for each walker.
    Returns
    -------
    x_all : 2D array
        The trajectory of the particle's position (m) for all walkers.
    v_all : 2D array
        The trajectory of the particle's velocity (m/s) for all walkers.
    t_all : 2D array
        The trajectory of the time (s) for all walkers.
    walkers : list of drudeWalker
        The list of drudeWalker objects for each walker."""
    kwargs = {'dt': 1e-15, 'mass': 9.1e-31, 'tau': 148.3e-15, 'L': 10e-9, 'elastic': True, 'R': 1}

    walkers = numba.typed.List()
    for n in range(NWalkers):
        walker = drudeWalker(**kwargs)
        walkers.append(walker)

    N = NSteps

    xs = numba.typed.List()
    vs = numba.typed.List()
    ts = numba.typed.List()
    for n in numba.prange(NWalkers):
        walker = walkers[n]
        x, v, t = walker.walk(N)
        xs.append(x)
        vs.append(v)
        ts.append(t)
    return xs, vs, ts, walkers

def plotDrudeWalkerN():
    """A test function to run the drude walker for many particles in parallel and plot the averaged results."""
    x_all, v_all, t_all, walkers = testDrudeWalkerN()
    # discard the first 1000 points to allow the walker to equilibrate
    N_discard = 1000
    NWalkers = len(x_all)
    x = np.zeros((NWalkers, len(x_all[0])-N_discard))
    v = np.zeros((NWalkers, len(v_all[0])-N_discard))
    t = np.zeros((NWalkers, len(t_all[0])-N_discard))
    for n in range(NWalkers):
        x[n,:] = x_all[n][N_discard:]
        v[n,:] = v_all[n][N_discard:]
        t[n,:] = t_all[n][N_discard:]
        t[n,:] -= t[n,0]  # reset time to start at zero
    # now average over all walkers

    Ct_all = []
    tCt_all = []
    for n in range(NWalkers):
        Ct, tCt = calcVelocityAutorCorrelationLog(v[n], t[n], Ct_span = 1000e-15, dt_absmin=0.001e-15, inc=1.001)
```

```python
        Ct_all.append(Ct)
        tCt_all.append(tCt)

    Ct_avg = np.zeros_like(Ct_all[0])
    tCt_avg = np.copy(Ct_avg)
    Ct_avg[:] = 0
    tCt_avg[:] = 0
    for n in range(NWalkers):
        Ct_avg += Ct_all[n]
        tCt_avg += tCt_all[n]
    Ct_avg /= NWalkers
    tCt_avg /= NWalkers

    plt.plot(tCt_avg, Ct_avg)
    plt.title('Velocity Autocorrelation Function')
    plt.show()

    # use the autocorrelation function to calculate the complex mobility
    wlist, mulist = calcComplexMobilityRange(Ct_avg, tCt_avg, 1e10, 1e15, 100)
    plt.plot(wlist, np.real(mulist))
    plt.plot(wlist, -np.imag(mulist))
    plt.xscale('log')
    plt.title('Complex Mobility from Kubo Formula')
    plt.show()



def testDrudeWalker():
    """A test function to run the drude walker and return the results."""
    kwargs = {'dt': 1e-15, 'mass': 9.1e-31, 'tau': 148.3e-15, 'L': 1e-9, 'elastic': False, 'R': 1}
    walker = drudeWalker(**kwargs)
    N = 100000000
    x, v, t = walker.walk(N)
    return x, v, t, walker



# A test function to plot the results of the drude walker
def plotDrudeWalker():
    x_all, v_all, t_all, walker = testDrudeWalker()
    # discard the first 1000 points to allow the walker to equilibrate
    N_discard = 10000
    x = x_all[N_discard:]
    v = v_all[N_discard:]
    t = t_all[N_discard:]
    t[:] -= t[0]  # reset time to start at zero

    print(walker.alpha)
    cores = 10# os.cpu_count()-1 #My M3 MacBook has 14 cores, but only 10 performance cores.
    # plot the input velocity distribution
    # plt.plot(walker.vDist_v, walker.vDist)
    # plt.show()

    # plot the raw position and velocity trajectories
    # plt.plot(t, x)
    # plt.show()
```

```
    # plt.plot(t, v)
    # plt.show()

    # plot the histograms of the scattering position and velocity distributions
    #make sure the velocity distribution matches the input distribution
    vdistout = plt.hist(v, bins=100, density=True)
    plt.plot(vdistout[1][1:], vdistout[0])
    plt.plot(walker.vDist_v, walker.vDist)
    plt.title('Histogram of Scattering Velocities')
    plt.show()

    # notice that a histogram of x does not provide the position distribution, but rather the frequency
    # of scattering events at each position. This leads to peaks in the distribution at the edges of the box
    # where the particle is reflected. The actual position distribution should be uniform across the box.
    xhistout = plt.hist(x, bins=100, density=True)
    plt.plot(xhistout[1][1:], xhistout[0])
    plt.title('Histogram of Scattering Positions')
    plt.show()

    # plot the histogram of the time intervals between scattering events. Make sure it matches the input distribution.
    dtarray = np.diff(t)
    thistout = plt.hist(dtarray, bins=100, density=True)
    plt.plot(thistout[1][1:], thistout[0])
    plt.title('Histogram of Scattering Time Intervals')
    plt.show()

    # calculate and plot the velocity autocorrelation function and complex mobility
    # calculate the autocorrelation function
    numba.set_num_threads(cores)
    #Ct, tCt = calcVelocityAutorCorrelation_paralell(v, t, Ct_span = 1000e-15, dt_absmin=0.1e-15, cores=cores)
    #Ct, tCt = calcVelocityAutorCorrelation(v, t, Ct_span = 100e-15, dt_absmin=0.1e-15)
    Ct, tCt = calcVelocityAutorCorrelationLog(v, t, Ct_span = 10000e-15, dt_absmin=0.01e-15, inc=1.001, lag=0.1)
    plt.plot(tCt, Ct)
    plt.axhline(0, color='gray', linestyle='--', linewidth=1)  # Add zero line
    plt.xscale('log')
    plt.title('Velocity Autocorrelation Function')
    plt.show()

    # use the autocorrelation function to calculate the complex mobility
    wlist, mulist = calcComplexMobilityRange(Ct, tCt, 1e12, 1e16, 100)
    plt.plot(wlist, np.real(mulist))
    plt.plot(wlist, -np.imag(mulist))
    plt.xscale('log')
    plt.title('Complex Mobility from Kubo Formula')
    plt.show()

enclosing_folder = os.path.dirname(os.path.abspath(__file__))

def sci_notation_to_str(number, precision=1):
    """
    Convert a number to a string in the format 'number x 10^exponent' with specified precision.
    Example: sci_notation_to_str(0.0001234, 2) -> '1.23 x 10^-4'
    """
    if number == 0:
        return f"0x10_0"
    exponent = int(np.floor(np.log10(abs(number))))
```

```
    mantissa = number / (10 ** exponent)
    return f"{mantissa:.{precision}f}x10_{exponent}"

def sci_notation_to_str_latex(number, precision=1):
    """
    Convert a number to a string in the format 'number x 10^exponent' with specified precision.
    Example: sci_notation_to_str(0.0001234, 2) -> '1.23 x 10^-4'
    """
    if number == 0:
        return f"0.0"
    exponent = int(np.floor(np.log10(abs(number))))
    mantissa = number / (10 ** exponent)
    return f"{mantissa:.{precision}f}x10$^{{{exponent}}}$"

def makeDrudeFigures():
    """Generate figures for the Drude model random walk and save them as EPS files."""

    #instantiate the drudeWalker class
    walker = drudeWalker(dt = 1e-15, mass = 9.1e-31, tau = 1000e-15, L=1000000e-9)

    # run it for a short time to get a trajectory plot
    N = 50
    x, v, t = walker.walk(N)




    fig = plt.figure(figsize=(5, 5))  # Set overall figure size to 5x5 inches
    # Make the axes fill the entire figure (plot area is 4x4)
    ax = fig.add_axes([0.15, 0.2, 0.65, 0.65])
    ax.plot(t, x, label=' position (m))')
    ax.yaxis.set_major_formatter(ScalarFormatter(useMathText=True))
    ax.ticklabel_format(style='sci', axis='y', scilimits=(0,0))
    ax.set_xlabel('time (s)', fontsize=14)
    ax.set_ylabel('position (m)', fontsize=14)
    ax.set_title('Drude Model Random Walk Trajectory')
    ax2 = ax.twinx()
    ax2.plot(t, v, color='orange', label='velocity (m/s)')
    ax2.set_ylabel('velocity (m/s)', fontsize=14)
    ax2.yaxis.set_major_formatter(ScalarFormatter(useMathText=True))
    ax2.ticklabel_format(style='sci', axis='y', scilimits=(0,0))
    #ax2.legend(loc='lower right', framealpha=0.75)

    #ax.legend(loc='upper right', framealpha=0.75)
    ax.set_xlim(t[0], t[-1])
    # Combine legends from both axes
    lines, labels = ax.get_legend_handles_labels()
    lines2, labels2 = ax2.get_legend_handles_labels()

    # Increase axes and tick line width
    lw = 1.5
    ax.spines['top'].set_linewidth(lw)
    ax.spines['right'].set_linewidth(lw)
    ax.spines['bottom'].set_linewidth(lw)
    ax.spines['left'].set_linewidth(lw)
    ax.tick_params(width=lw, length=6, labelsize=12)
```

```python
#plt.tight_layout(pad=1)

# Ensure the legend appears on top of all traces
leg = ax.legend(lines + lines2, labels + labels2, loc='upper right', framealpha=0.75, fontsize=14)
leg.set_zorder(1e9)  # Set a high z-order so legend is drawn above all plot elements
eps_path = os.path.join(enclosing_folder, "drude_position_and_velocity.eps")
plt.savefig(eps_path, format='eps')
print(f"Figure saved to {eps_path}")
plt.show()

# run it for a long time to get the velocity autocorrelation function
N = 1000000
x, v, t = walker.walk(N)

# calculate the autocorrelation function
Ct, tCt = calcVelocityAutorCorrelationLog(v, t, Ct_span = 10000e-15, dt_absmin=10e-15, lag = 0.1)
fig = plt.figure(figsize=(5, 5))  # Set overall figure size to 5x4 inches

# Make the axes fill the entire figure (plot area is 4x3)
ax = fig.add_axes([0.15, 0.2, 0.65, 0.65])
ax.plot(tCt, Ct, label='Velocity Autocorrelation')
ax.yaxis.set_major_formatter(ScalarFormatter(useMathText=True))
ax.ticklabel_format(style='sci', axis='y', scilimits=(0,0))
ax.set_xlabel('time (s)', fontsize=14)
ax.set_ylabel('Velocity Autocorrelation (m$^2$/s$^2$)', fontsize=14)
ax.set_title('Velocity Autocorrelation Function', fontsize=14)
ax.set_xlim(tCt[0], tCt[-1])
# Increase axes and tick line width
lw = 1.5
ax.spines['top'].set_linewidth(lw)
ax.spines['right'].set_linewidth(lw)
ax.spines['bottom'].set_linewidth(lw)
ax.spines['left'].set_linewidth(lw)
ax.tick_params(width=lw, length=6, labelsize=12)
#plt.tight_layout(pad=1)
ax.legend(loc='upper right', framealpha=0.75, fontsize=14)
eps_path = os.path.join(enclosing_folder, "drude_velocity_autocorrelation.eps")
plt.savefig(eps_path, format='eps')
print(f"Figure saved to {eps_path}")
plt.show()

# use the autocorrelation function to calculate the complex mobility
wlist, mulist = calcComplexMobilityRange(Ct, tCt, 1e9, 1e13, 100)

#Make the figure
fig = plt.figure(figsize=(5, 5))  # Set overall figure size to 5x4 inches
# Make the axes fill the entire figure (plot area is 4x3)
ax = fig.add_axes([0.15, 0.2, 0.65, 0.65])
ax.plot(wlist, np.real(mulist), label='Re[$\mu(\omega)$]')
ax.plot(wlist, np.imag(mulist), label='Im[$\mu(\omega)$]')
ax.set_xscale('log')
ax.yaxis.set_major_formatter(ScalarFormatter(useMathText=True))
ax.ticklabel_format(style='sci', axis='y', scilimits=(0,0))
ax.set_xlabel('Angular Frequency (rad/s)', fontsize=14)
ax.set_ylabel('Complex Mobility (m$^2$/Vs)', fontsize=14)
ax.set_title('Complex Mobility from Velocity Autocorrelation', fontsize=14)
```

```python
    ax.set_xlim(wlist[0], wlist[-1])
    # Increase axes and tick line width
    lw = 1.5
    ax.spines['top'].set_linewidth(lw)
    ax.spines['right'].set_linewidth(lw)
    ax.spines['bottom'].set_linewidth(lw)
    ax.spines['left'].set_linewidth(lw)
    ax.tick_params(width=lw, length=6, labelsize=12)
    #plt.tight_layout(pad=1)
    ax.legend(loc='upper right', framealpha=0.75, fontsize=14)
    eps_path = os.path.join(enclosing_folder, "drude_complex_mobility.eps")
    plt.savefig(eps_path, format='eps')
    print(f"Figure saved to {eps_path}")
    plt.show()


def makeConfinedDrudeFigures(calc = True):
    """
    Make figures showing the effect of confinement on the velocity autocorrelation function
    calc : bool
        If True, calculate new trajectories and save them to files.
        If False, load existing trajectories from files.
    """

    if calc:
        Npoints = 100000000
        # calcualte a drude walker in a small box
        kwargs = {'dt': 1e-15, 'mass': 9.1e-31, 'tau': 148.3e-15, 'L': 100e-9, 'elastic': False, 'R': 1}
        walker = drudeWalker(**kwargs)
        x0, v0, t0 = walker.walk(Npoints)
        traj0 = np.vstack((x0, v0, t0))
        traj0_path = os.path.join(enclosing_folder, "drude_trajectory_L100nm.txt")
        np.savetxt(traj0_path, traj0)
        print(f"Trajectory saved to {traj0_path}")

        # calcualte the Drude walker in a medium box
        kwargs = {'dt': 1e-15, 'mass': 9.1e-31, 'tau': 148.3e-15, 'L': 500e-9, 'elastic': False, 'R': 1}
        walker = drudeWalker(**kwargs)
        x1, v1, t1 = walker.walk(Npoints)
        traj1 = np.vstack((x1, v1, t1))
        traj1_path = os.path.join(enclosing_folder, "drude_trajectory_L500nm.txt")
        np.savetxt(traj1_path, traj1)
        print(f"Trajectory saved to {traj1_path}")

        # calcualte the Drude walker in a big box
        kwargs = {'dt': 1e-15, 'mass': 9.1e-31, 'tau': 148.3e-15, 'L': 10000e-9, 'elastic': False, 'R': 1}
        walker = drudeWalker(**kwargs)
        x2, v2, t2 = walker.walk(Npoints)
        traj2 = np.vstack((x2, v2, t2))
        traj2_path = os.path.join(enclosing_folder, "drude_trajectory_L10000nm.txt")
        np.savetxt(traj2_path, traj2)
        print(f"Trajectory saved to {traj2_path}")

    else:
        traj0_path = os.path.join(enclosing_folder, "drude_trajectory_L100nm.txt")
        traj0 = np.loadtxt(traj0_path)
```

```
    x0 = traj0[0,:]
    v0 = traj0[1,:]
    t0 = traj0[2,:]

    traj1_path = os.path.join(enclosing_folder, "drude_trajectory_L500nm.txt")
    traj1 = np.loadtxt(traj1_path)
    x1 = traj1[0,:]
    v1 = traj1[1,:]
    t1 = traj1[2,:]

    traj2_path = os.path.join(enclosing_folder, "drude_trajectory_L10000nm.txt")
    traj2 = np.loadtxt(traj2_path)
    x2 = traj2[0,:]
    v2 = traj2[1,:]
    t2 = traj2[2,:]


# calcualte the correlation functions:
# note that the results are quite sensitive to how long you compute the correlation function out to.
# too short, and truncation error gives spurious oscilations in the complex mobility.
# too long and a great deal of excess noise will appear.
Ct0, tCt0 = calcVelocityAutorCorrelationLog(v0, t0, Ct_span = 40000e-15, dt_absmin=1e-17, inc=1.0001, lag=0.1)
Ct1, tCt1 = calcVelocityAutorCorrelationLog(v1, t1, Ct_span = 40000e-15, dt_absmin=1e-17, inc=1.0001, lag=0.1)
Ct2, tCt2 = calcVelocityAutorCorrelationLog(v2, t2, Ct_span = 40000e-15, dt_absmin=1e-17, inc=1.0001, lag=0.1)

# plot the correlation functions together
fig = plt.figure(figsize=(5, 5))  # Set overall figure size to 5x4 inches
# Make the axes fill the entire figure (plot area is 4x3)
ax = fig.add_axes([0.15, 0.2, 0.65, 0.65])
ax.plot(tCt0, Ct0, label='L=100 nm')
ax.plot(tCt1, Ct1, label='L=500 nm')
ax.plot(tCt2, Ct2, label='L=10000 nm')
ax.yaxis.set_major_formatter(ScalarFormatter(useMathText=True))
ax.ticklabel_format(style='sci', axis='y', scilimits=(0,0))
ax.set_xlabel('time (s)')
ax.set_ylabel('Velocity Autocorrelation (m$^2$/s$^2$)')
ax.set_title('Velocity Autocorrelation Function, Confined')
ax.set_xlim(tCt0[0], tCt0[-1])
# Increase axes and tick line width
lw = 1.5
ax.spines['top'].set_linewidth(lw)
ax.spines['right'].set_linewidth(lw)
ax.spines['bottom'].set_linewidth(lw)
ax.spines['left'].set_linewidth(lw)
ax.tick_params(width=lw, length=6)
ax.set_xlim(1e-14, tCt0[-1])
ax.set_xscale('log')
#plt.tight_layout(pad=1)
ax.legend(loc='upper right', framealpha=0.75)
eps_path = os.path.join(enclosing_folder, "drude_velocity_autocorrelation_confined.eps")
plt.savefig(eps_path, format='eps')
print(f"Figure saved to {eps_path}")
plt.show()

#Calculate and plot the complex mobility for each case
wlist0, mulist0 = calcComplexMobilityRange(Ct0, tCt0, 1e11, 1e15, 100)
```

```python
    wlist1, mulist1 = calcComplexMobilityRange(Ct1, tCt1, 1e11, 1e15, 100)
    wlist2, mulist2 = calcComplexMobilityRange(Ct2, tCt2, 1e11, 1e15, 100)

    fig = plt.figure(figsize=(5, 5))  # Set overall figure size to 5x4 inches
    # Make the axes fill the entire figure (plot area is 4x3)
    ax = fig.add_axes([0.15, 0.2, 0.65, 0.65])
    ax.plot(wlist0, np.real(mulist0), label='Re[$\mu(\omega)$], L=100 nm', color='C0', linestyle='-')
    ax.plot(wlist0, np.imag(mulist0), label='Im[$\mu(\omega)$]', color='C0', linestyle='--')
    ax.plot(wlist1, np.real(mulist1), label='Re[$\mu(\omega)$], L=500 nm', color='C1', linestyle='-')
    ax.plot(wlist1, np.imag(mulist1), label='Im[$\mu(\omega)$]', color='C1', linestyle='--')
    ax.plot(wlist2, np.real(mulist2), label='Re[$\mu(\omega)$], L=10000 nm', color='C2', linestyle='-')
    ax.plot(wlist2, np.imag(mulist2), label='Im[$\mu(\omega)$]', color='C2', linestyle='--')
    ax.set_xscale('log')
    ax.yaxis.set_major_formatter(ScalarFormatter(useMathText=True))
    ax.ticklabel_format(style='sci', axis='y', scilimits=(0,0))
    ax.set_xlabel('Angular Frequency (rad/s)')
    ax.set_ylabel('Complex Mobility (m$^2$/Vs)')
    ax.set_title('Complex Mobility from Velocity Autocorrelation, Confined')
    ax.set_xlim(wlist0[0], wlist0[-1])
    # Increase axes and tick line width
    lw = 1.5
    ax.spines['top'].set_linewidth(lw)
    ax.spines['right'].set_linewidth(lw)
    ax.spines['bottom'].set_linewidth(lw)
    ax.spines['left'].set_linewidth(lw)
    ax.tick_params(width=lw, length=6)
    #plt.tight_layout(pad=1)
    ax.legend(loc='upper right', framealpha=0.75)
    eps_path = os.path.join(enclosing_folder, "drude_complex_mobility_confined.eps")
    plt.savefig(eps_path, format='eps')
    print(f"Figure saved to {eps_path}")
    plt.show()



#Testing the Drude kMC model. Uncomment to execute.
##################################
#calcGaussRates(1, 0.1)
#plotDrudeWalker()
#plotDrudeWalkerN()

# make the Drude kMC figures for the unconfined case.
makeDrudeFigures()

# compute/make the figures for Drude kMC with confinement
# make calc = True if you haven't already computed the kMC result.



#makeConfinedDrudeFigures(calc=False)
```

2.3 complex_conductivity_analytic.py

```
"""Complex conductivity analytic models for confined charge carriers in nanostructures.
Inclides:
                        - Confined diffusion model (Prins et al. 2006)
                        - Modified Drude-Smith model (Cocker et al. 2017)
                           - Semiclassical model (Kuzel & Nemec 2019, Ostatnicky 2019, Ostatnicky et al. 2018)
                           - Drude-Smith model (Smith 2001)
"""

from __future__ import annotations

from dataclasses import dataclass
from typing import Tuple

import numpy as np
import scipy as scipy
import math as math

_KB = 1.38e-23 #boltzmann's constant
_Q = 1.602e-19 #electron charge
_ME = 9.109e-31 #electron rest mass.

def mu_from_tau(tau: float, mer: float = 1.0) -> float:
        """Calculate mobility from scattering time using mu = q*tau/m.

        Parameters
        ----------
        tau : float
                Scattering time in seconds
        mer : float, optional
                Effective mass ratio (m*/m_e), default is 1.0

        Returns
        -------
        float
                Mobility in m²/V/s
        """
        m = _ME * mer
        mu = _Q * tau / m
        #print (mu)
        return mu

'''
'confined diffusion model' from:
 @article{Prins_Grozema_Schins_Siebbeles_2006, title={Frequency dependent mobility of charge carriers along polymer chains with finite length},
↪  volume={243}, DOI={10.1002/pssb.200562719}, abstractNote={The effect of the chain length of conjugated polymers on the real and imaginary
↪  components of the frequency dependent mobility of charge carriers is studied. A theoretical model for one-dimensional d...}, number={2},
↪  journal={physica status solidi (b)}, author={Prins, Paulette and Grozema, Ferdinand C and Schins, J M and Siebbeles, Laurens D A},
↪  year={2006}, month=feb, pages={382-386}, language={English} }

'''

def confined_diffusion(N: int, tau: float, mer: float, L: float, f: float, T: float = 300) -> complex:
        """Compute complex mobility using the Kubo summation.
        parameters:
```

```
            N: int - number of terms to include in the summation (truncation point)
            tau: float - scattering time in seconds
            mer: float - effective mass ratio (m*/m_e)
            L: float - characteristic length scale (e.g., grain size) in meters
            f: float - frequency in Hz
            T: float - temperature in Kelvin (default 300K)
    returns:
    -------
    mu_ac: complex - Complex mobility (m²/V/s)
    """
    mu = mu_from_tau(tau, mer) # calcualte mobility from scattering time.
    k = _KB
    e = _Q
    w = f * 2.0 * np.pi
    i=0
    mu_ac = 0+0j
    while i < N: #truncate at N terms of the infinite sum.
            Ck = 2.0 * np.pi * (i + 0.5)
            term = (Ck**2 * (k * T * mu * Ck**2 / (1j * e * w * L**2) + 1.0)) ** (-1)
            mu_ac += term * 8.0 * mu
            i+=1

    return mu_ac


@dataclass # a class to pack the data into.
class ConfinedDiffusionPlotData:
    frequency: np.ndarray
    mu_re: np.ndarray
    mu_im: np.ndarray


def calc_confined_diffusion(tau: float=0.1e-12, L: float=100e-9, mer: float=1, T: float = 300, n_points: int = 100, F0: float = 0.1e9, F1: float =
↪ 2000e9) -> ConfinedDiffusionPlotData:
    """Return data arrays for real/imag parts over frequency range.
    parameters:
            tau: float - scattering time in seconds
            L: float - characteristic length scale (e.g., grain size) in meters
            mer: float - effective mass ratio (m*/m_e)
            T: float - temperature in Kelvin (default 300K)
            n_points: int - number of frequency points
            F0: float - starting frequency in Hz
            F1: float - ending frequency in Hz
    returns:
            ConfinedDiffusionPlotData - dataclass containing frequency array and real/imag mobility arrays
    """
    N = 20


    frequencies= np.logspace(np.log10(F0), np.log10(F1), n_points) # use log-spaced frequencies
    mu_ac = np.empty_like(frequencies, dtype=complex)
    for i in range(n_points):
            mu_ac[i] = confined_diffusion(N, tau, mer, L, frequencies[i], T)
    return ConfinedDiffusionPlotData(
            frequency=frequencies,
            mu_re=np.real(mu_ac),
```

```python
            mu_im=np.imag(mu_ac),
    )


def plot_confined_diffusion_graph(tau: float, L: float, mer: float, n_points: int = 100) -> None:
    """Generate and display a matplotlib graph of confined diffusion complex mobility."""
    try:
        import matplotlib.pyplot as plt
    except ImportError as exc:  # pragma: no cover
        raise ImportError("matplotlib is required for plot_confined_diffusion_graph") from exc

    data = calc_confined_diffusion(tau, L, mer, n_points)

    fig, (ax1, ax2) = plt.subplots(1, 2, figsize=(14, 5))

    # Plot real part
    ax1.plot(data.frequency / 1e9, data.mu_re, 'b-', linewidth=2)
    ax1.set_xlabel('Frequency (GHz)', fontsize=12)
    ax1.set_ylabel('Re[_ac] (m²/V/s)', fontsize=12)
    ax1.set_title('Real Part of Complex AC Mobility (Confined Diffusion)', fontsize=13)
    ax1.set_xscale('log')
    ax1.set_yscale('log')
    ax1.grid(True, alpha=0.3)

    # Plot imaginary part
    ax2.plot(data.frequency / 1e9, data.mu_im, 'r-', linewidth=2)
    ax2.set_xlabel('Frequency (GHz)', fontsize=12)
    ax2.set_ylabel('Im[_ac] (m²/V/s)', fontsize=12)
    ax2.set_title('Imaginary Part of Complex AC Mobility (Confined Diffusion)', fontsize=13)
    ax2.set_xscale('log')
    ax2.grid(True, alpha=0.3)

    plt.tight_layout()
    plt.savefig('confined_diffusion_mobility.png', dpi=300, bbox_inches='tight')
    print("Saved plot to confined_diffusion_mobility.png")

    plt.show()

#plot_confined_diffusion_graph(tau =0.270e-12, L = 1e-7, mer=0.07, n_points = 200)

'''
modified drude-smith model from:
 @article{Cocker_Baillie_Buruma_Titova_Sydora_Marsiglio_Hegmann_2017, title={Microscopic origin of the Drude-Smith model}, volume={96}, ISSN={2469-9950, 2469-9969}, DOI={10.1103/PhysRevB.96.205439}, number={20}, journal={Physical Review B}, author={Cocker, T. L. and Baillie, D. and Buruma, M. and Titova, L. V. and Sydora, R. D. and Marsiglio, F. and Hegmann, F. A.}, year={2017}, month=nov, pages={205439}, language={en} }

'''

def mdrude_smith(
    f: np.ndarray | float,
    mer: float,
    tau: float,
    T: float,
    L: float,
) -> np.ndarray | complex:
```

```python
    """Modified Drude-Smith conductivity (SI units).
    parameters:
        f: float or ndarray - frequency in Hz
        mer: float - effective mass ratio (m*/m_e)
        tau: float - scattering time in seconds
        T: float - temperature in Kelvin
        L: float - characteristic length scale (e.g., grain size) in meters
    returns:
        complex or ndarray - complex mobility in m²/V/s
    """
    Vth = np.sqrt(_KB * T / (_ME * mer))
    tp = (1.0 / tau + 2.0 * Vth / L) ** (-1)
    t0 = L / Vth
    a = 12.0 / t0 * (tau / (t0 + 2.0 * tau))
    w = 2.0 * np.pi * f
    mu = (_Q**2* tp / (_ME * mer) / (1.0 - 1j * w * tp) * (1.0 - 1.0 / (1.0 - 1j * w / a))
    )

    mu /= _Q#**2 * tau / (_ME * mer)
    return mu

@dataclass
class MDSPlotData:
    frequency: np.ndarray
    mu_re: np.ndarray
    mu_im: np.ndarray

def calc_mdrude_smith(tau: float, L: float, mer: float = 1, n_points: int = 100, F0 = 0.1e9, F1 = 2000e9) -> MDSPlotData:
    """Return data arrays for real/imag parts of mDS mobility over frequency range.
    parameters:
        tau: float - scattering time in seconds
        L: float - characteristic length scale (e.g., grain size) in meters
        mer: float - effective mass ratio (m*/m_e)
        n_points: int - number of frequency points
        F0: float - starting frequency in Hz
        F1: float - ending frequency in Hz
    returns:
        MDSPlotData - dataclass containing frequency array and real/imag mobility arrays
    """
    #mer = 1
    T = 300.0
    frequencies= np.logspace(np.log10(F0), np.log10(F1), n_points)
    mu_mds = mdrude_smith(frequencies, mer, tau, T, L)
    return MDSPlotData(
        frequency=frequencies,
        mu_re=np.real(mu_mds),
        mu_im=np.imag(mu_mds),
    )

def plot_mDS_graph(tau: float, L: float, mer: float, n_points: int = 100) -> None:
    """Generate and display a matplotlib graph of modified Drude-Smith complex mobility."""
    try:
        import matplotlib.pyplot as plt
    except ImportError as exc:  # pragma: no cover
        raise ImportError("matplotlib is required for plot_mDS_graph") from exc
```

```
        data = calc_mdrude_smith(tau, L, mer, n_points)

        fig, (ax1, ax2) = plt.subplots(1, 2, figsize=(14, 5))

        # Plot real part
        ax1.plot(data.frequency / 1e9, data.mu_re, 'b-', linewidth=2)
        ax1.set_xlabel('Frequency (GHz)', fontsize=12)
        ax1.set_ylabel('Re[_mDS] (m²/V/s)', fontsize=12)
        ax1.set_title('Real Part of Complex AC Mobility (Modified Drude-Smith)', fontsize=13)
        ax1.set_xscale('log')
        ax1.grid(True, alpha=0.3)

        # Plot imaginary part
        ax2.plot(data.frequency / 1e9, data.mu_im, 'r-', linewidth=2)
        ax2.set_xlabel('Frequency (GHz)', fontsize=12)
        ax2.set_ylabel('Im[_mDS] (m²/V/s)', fontsize=12)
        ax2.set_title('Imaginary Part of Complex AC Mobility (Modified Drude-Smith)', fontsize=13)
        ax2.set_xscale('log')
        ax2.grid(True, alpha=0.3)

        plt.tight_layout()
        plt.savefig('mds_mobility.png', dpi=300, bbox_inches='tight')
        print("Saved plot to mds_mobility.png")

        plt.show()

#plot_mDS_graph(tau = 0.270e-12, L = 1e-7, mer=0.07, n_points = 200)


'''
"semiclassical model"
 @article{Kužel_Němec_2019, title={Terahertz Spectroscopy of Nanomaterials: a Close Look at Charge-Carrier Transport}, volume={4}, DOI={10.1002/adom.201900623}, journal={Advanced Optical Materials}, author={Kužel, Petr and Němec, Hynek}, year={2019}, month=jun, pages={1900623-23}, language={English} }
 @article{Ostatnický_2019, title={Linear THz conductivity of nanocrystals}, volume={27}, rights={© 2019 Optical Society of America}, ISSN={1094-4087}, DOI={10.1364/OE.27.006083}, abstractNote={We derive a simple, semi-classical formula for the calculation of the linear electron conductivity spectrum of semiconductor nanocrystals in the THz spectral range from the general quantum-mechanical model. We show that the reliability and range of applicability of the formula significantly exceed the scope of both the Drude-Smith model and the recently developed modified Drude-Smith model.}, number={5}, journal={Optics Express}, publisher={Optica Publishing Group}, author={Ostatnický, T.}, year={2019}, month=mar, pages={6083-6088}, language={EN} }
 @article{Ostatnický_Pushkarev_Němec_Kužel_2018, title={Quantum theory of terahertz conductivity of semiconductor nanostructures}, volume={97}, DOI={10.1103/PhysRevB.97.085426}, abstractNote={Efficient and controlled charge carrier transport through nanoelements is currently a primordial question in the research of nanoelectronic materials and structures. We develop a quantum-mechanical theory of the conductivity spectra of confined charge carriers responding to an electric field from dc regime up to optical frequencies. The broken translation symmetry induces a broadband drift-diffusion current, which is not taken into account in the analysis based on Kubo formula and relaxation time approximation. We show that this current is required to ensure that the dc conductivity of isolated nanostructures correctly attains zero. It causes a significant reshaping of the conductivity spectra up to terahertz or multiterahertz spectral ranges, where the electron scattering rate is typically comparable to or larger than the probing frequency.}, number={8}, journal={Physical Review B}, publisher={American Physical Society}, author={Ostatnický, T. and Pushkarev, V. and Němec, H. and Kužel, P.}, year={2018}, month=feb, pages={085426} }


'''
def semiclassical(
        f: np.ndarray | float,
        mer: float = 1.0,
        tau: float = 0.1e-12,
```

```
        T: float = 300.0,
        L: float = 1e-7,
) -> np.ndarray | complex:
        """Semiclassical (SC) model for complex AC conductivity (SI units).

        A classical approximation to a full quantum treatment of AC conductivity in semiconductor nanocrystals.

        Parameters
        ----------
        f : float or ndarray
                Frequency in Hz
        mer : float
                Effective mass ratio (m*/m_e)
        tau : float
                Scattering time in seconds
        T : float
                Temperature in Kelvin
        L : float
                Characteristic length scale (e.g., grain size) in meters

        Returns
        -------
        complex or ndarray
                Complex conductivity in S/m
        """
        gam = 1/tau
        m = _ME * mer

        #mu0 = _Q*tau/m
        D = _KB * T /(m*gam)

        Vth = np.sqrt(_KB * T /m)
        tth = L/(np.pi*np.sqrt(2)*Vth)
        tth = L*np.sqrt(m/(2*np.pi**2*_KB*T))
        gD = (D*np.pi**2)/(L**2)
        tD = 1/gD
        w = 2.0 * np.pi * f
        exce = (1-1j*w*tau)*tth/tau


        def ep(p):
                return (w + 1j*gam)*tth/p

        def erfcx(z):
                '''
                tried a few implementations here before I learned that there was a better scipy implementation.
                none of the earlier versions work for as wide a parameter range.
                '''
                #return np.exp(z**2)*(scipy.special.erfc(z))
                #return np.exp(z**2)*(1-scipy.special.erf(z))
                #return np.exp(z**2)*(1-math.erf(z))
                return scipy.special.erfcx(z)


        mu =0.0+0.0j
        p = 1
```

```
	while p<13:
		#from ostatnicky, 2018
		# mu +=( ep(p)/p**3
		# *(1-1/(1-1j*w/gam)
		# *1/(1-1j*w/p**2*gD))
		# *(1+1j*np.sqrt(np.pi)*ep(p)*erfcx(-1j*ep(p)))
		# )

		#from Kuzel 2020
		mu += (
			1/p**5
			*(exce-tth/tau*1/(1-1j*w*tD/p**2))
			*(p-np.sqrt(np.pi)*exce*erfcx(exce/p))
		)

		p+=2
	#from ostatnicky, 2018
	# mu *=-_Q*8*np.sqrt(2)*1j*L/(np.pi**3*m*Vth)*1e4
	#from Kuzel 2020
	mu*= 16*_Q*tth/(np.pi**2*m)
	return mu


#print (semiclassical(10e9, 1.0, 0.05e-12, 300.0, 1e-7))

@dataclass
class SemiclassicalData:
	frequency: np.ndarray
	mu_re: np.ndarray
	mu_im: np.ndarray



def calc_semiclassical(
	F0: float,
	F1: float,
	n_points: int,
	mer: float,
	tau: float,
	T: float,
	L: float,
) -> SemiclassicalData:
	"""Compute normalized semiclassical conductivity over log-spaced frequency.

	Parameters
	----------
	F0 : float
		Low frequency in Hz
	F1 : float
		High frequency in Hz
	n_points : int
		Number of frequency points
	mer : float
		Effective mass ratio (m*/m_e)
	tau : float
		Scattering time in seconds
	T : float
```

```
            Temperature in Kelvin
        L : float
            Characteristic length scale in meters

        Returns
        -------
        SemiclassicalData
            Frequency array and normalized real/imaginary conductivity
        """
        frequencies= np.logspace(np.log10(F0), np.log10(F1), n_points)

        mu = semiclassical(frequencies, mer, tau, T, L)
        return SemiclassicalData(
            frequency=frequencies,
            mu_re=np.real(mu),
            mu_im=np.imag(mu),
        )


def plot_semiclassical_graph(tau: float, L: float, mer: float, n_points: int = 100, F0 = 0.1e9, F1 = 20000e9) -> None:
        """Generate and display a matplotlib graph of semiclassical complex mobility."""
        try:
            import matplotlib.pyplot as plt
        except ImportError as exc:  # pragma: no cover
            raise ImportError("matplotlib is required for plot_semiclassical_graph") from exc

        SCdata = calc_semiclassical(F0, F1, n_points, mer, tau, 300.0, L)

        fig, (ax1, ax2) = plt.subplots(1, 2, figsize=(14, 5))

        # Plot real part
        ax1.plot(SCdata.frequency / 1e9, SCdata.mu_re, 'b-', linewidth=2)
        ax1.set_xlabel('Frequency (GHz)', fontsize=12)
        ax1.set_ylabel('Re[_SC] (m²/V/s)', fontsize=12)
        ax1.set_title('Real Part of Complex AC Mobility (Semiclassical)', fontsize=13)
        ax1.set_xscale('log')
        #ax1.set_yscale('log')
        ax1.grid(True, alpha=0.3)

        # Plot imaginary part
        ax2.plot(SCdata.frequency / 1e9, SCdata.mu_im, 'r-', linewidth=2)
        ax2.set_xlabel('Frequency (GHz)', fontsize=12)
        ax2.set_ylabel('Im[_SC] (m²/V/s)', fontsize=12)
        ax2.set_title('Imaginary Part of Complex AC Mobility (Semiclassical)', fontsize=13)
        ax2.set_xscale('log')
        ax2.grid(True, alpha=0.3)

        plt.tight_layout()
        plt.savefig('semiclassical_mobility.png', dpi=300, bbox_inches='tight')
        print("Saved plot to semiclassical_mobility.png")

        plt.show()

#plot_semiclassical_graph(tau = 0.27e-12, L = 100e-9, mer=0.07, n_points = 200)

'''
```

```python
Drude model
'''

'''
drude-smith model
'''

def drudeSmith(
        f: np.ndarray | float,
        mer: float = 1.0,
        tau: float = 0.1e-12,
        T: float = 300.0,
        c: float = 1,
) -> np.ndarray | complex:
        """Drude-Smith model for complex AC conductivity (SI units).

        Parameters
        ----------
        f : float or ndarray
                Frequency in Hz
        mer : float
                Effective mass ratio (m*/m_e)
        tau : float
                Scattering time in seconds
        T : float
                Temperature in Kelvin
        c : float
                Drude-Smith parameter (0 <= c <= 1)

        Returns
        -------
        complex or ndarray
                Complex conductivity in S/m
        """
        m = _ME * mer
        w = 2.0 * np.pi * f
        mu0 = _Q * tau / m
        muds = mu0/(1-1j*w*tau)*(1+c/(1-1j*w*tau))
        return muds


#print (semiclassical(10e9, 1.0, 0.05e-12, 300.0, 1e-7))

@dataclass
class dsData:
        frequency: np.ndarray
        mu_re: np.ndarray
        mu_im: np.ndarray


def calc_drudeSmith(
        F0: float,
        F1: float,
        n_points: int,
        mer: float,
        tau: float,
```

```
        T: float,
        c: float,
) -> dsData:
        """Compute drude-smith conductivity over log-spaced frequency.

        Parameters
        ----------
        F0 : float
                Low frequency in Hz
        F1 : float
                High frequency in Hz
        n_points : int
                Number of frequency points
        mer : float
                Effective mass ratio (m*/m_e)
        tau : float
                Scattering time in seconds
        T : float
                Temperature in Kelvin
        c : float
                Drude-Smith parameter (0 <= c <= 1)

        Returns
        -------
        dsData
                Frequency array and normalized real/imaginary conductivity
        """
        frequencies= np.logspace(np.log10(F0), np.log10(F1), n_points)

        mu = drudeSmith(frequencies, mer, tau, T, c)
        return dsData(
                frequency=frequencies,
                mu_re=np.real(mu),
                mu_im=np.imag(mu),
        )


def plot_all_graph(tau: float, L: float, mer: float, n_points: int = 100, F0 = 0.1e9, F1 = 200000e9) -> None:
        """Generate and display a matplotlib graph of semiclassical complex mobility."""
        try:
                import matplotlib.pyplot as plt
        except ImportError as exc:  # pragma: no cover
                raise ImportError("matplotlib is required for plot_semiclassical_graph") from exc
        T = 300
        SCdata = calc_semiclassical(F0, F1, n_points, mer, tau, T, L)
        mDSdata = calc_mdrude_smith(F0=F0, F1=F1, n_points=n_points, mer=mer, tau=tau, L=L)
        CDMdata = calc_confined_diffusion(tau, L, mer, T, n_points, F0, F1)
        DSdata = calc_drudeSmith(F0, F1, n_points, mer, tau, T, -1)
        drudedata = calc_drudeSmith(F0, F1, n_points, mer, tau, T, 0)

        fig, (ax1, ax2) = plt.subplots(1, 2, figsize=(14, 5))

        # Plot real part
        ax1.plot(SCdata.frequency / 1e9, SCdata.mu_re, 'b-', linewidth=2)
        ax1.plot(mDSdata.frequency / 1e9, mDSdata.mu_re, 'r-', linewidth=2)
        ax1.plot(CDMdata.frequency / 1e9, CDMdata.mu_re, 'g-', linewidth=2)
```

```
        ax1.plot(DSdata.frequency / 1e9, DSdata.mu_re, 'm-', linewidth=2)
        ax1.plot(drudedata.frequency / 1e9, drudedata.mu_re, 'k-', linewidth=2)
        #ax1.yaxis.set_major_formatter(ScalarFormatter(useMathText=True))
        ax1.set_xlabel('Frequency (GHz)', fontsize=12)
        ax1.set_ylabel('Re[$_{AC}$] (m$^2$/V/s)', fontsize=12)
        ax1.set_title('Real Part of Complex AC Mobility', fontsize=13)
        ax1.set_xscale('log')
        #ax1.set_yscale('log')
        ax1.grid(True, alpha=0.3)
        ax1.legend(['Semiclassical', 'Modified Drude-Smith', 'Confinded Diffusion', 'Drude-Smith', 'Drude'], fontsize=10)

        # Plot imaginary part
        ax2.plot(SCdata.frequency / 1e9, SCdata.mu_im, 'b-', linewidth=2)
        ax2.plot(mDSdata.frequency / 1e9, mDSdata.mu_im, 'r-', linewidth=2)
        ax2.plot(CDMdata.frequency / 1e9, CDMdata.mu_im, 'g-', linewidth=2)
        ax2.plot(DSdata.frequency / 1e9, DSdata.mu_im, 'm-', linewidth=2)
        ax2.plot(drudedata.frequency / 1e9, drudedata.mu_im, 'k-', linewidth=2)
        #ax2.yaxis.set_major_formatter(ScalarFormatter(useMathText=True))
        ax2.set_xlabel('Frequency (GHz)', fontsize=12)
        ax2.set_ylabel('Im[$_{AC}$] (m$^2$/V/s)', fontsize=12)
        ax2.set_title('Imaginary Part of Complex AC Mobility', fontsize=13)
        ax2.set_xscale('log')
        ax2.grid(True, alpha=0.3)
        ax2.legend(['Semiclassical', 'Modified Drude-Smith', 'Confined Diffusion', 'Drude-Smith', 'Drude'], fontsize=10)

        plt.tight_layout()
        plt.savefig('semiclassical_mobility.png', dpi=300, bbox_inches='tight')
        print("Saved plot to semiclassical_mobility.png")

        plt.show()

plot_all_graph(tau = 0.003e-12, L = 500e-9, mer=0.07, n_points = 200)
```

3 Frozen Igor Code:

3.1 ODE_Equations.ipf

```
        #pragma TextEncoding = "UTF-8"
#pragma rtGlobals=3                // Use modern global access method and strict wave access.

// these are the constants and equations used as input to the ODE solving and fitting functions.
// change these parameters to define your problem.
// adopt this procedure into your particular experiment, but not the others.
// this way your problem definiation remains local to the Igor experiment,
// but the solver machinery can continue to evolve independantly.

//Reuqired for all experiments:

StrConstant ksEqnNames = "h;e;ht;"//T3;" // write down the names of the species you want to track.
StrConstant ksParameters = "position;width;N0;cN0;mue;muh;gcr;gnr;Nt;Ne;Et;kdt0;g0;fc;gi;V;kex;L;T;Vbi;Eg;dEg;abs;solver;transient;injection;"//kT23;kT3G"        // write
                                                                                                                                                                     // these t
StrConstant ksParameterVals = "50e-9;2.25e-9;1e16;2.5e22;0.05;0.05;1e-10;1e-10;1e16;1e16;0.1;1e5;0;1;1e10;0;1e8;100e-7;300;1;1.1;0;0.01;3;0;0;"//5e9;1e8;" // write down i
```

```
StrConstant ksMDFR = "root:packages:OR_Functions:DiffEq" // path in which to store all the bits and pieces.
constant kNumPoints = 500 // number of data points per decade of log time.
constant ktRes = 10e-10 // time per point in seconds
Constant ktoffset = -10e-9 // beginning of time for the simulation. Choose this to be equal to or less than the most negative time point in your data. Cannot be zero, pos
Constant ktMax = 30000e-9//100000e-9  // end of time for the simulation. Chose this to be a equal to or later than the end of your data.
Constant kMagCoef = 6 // which coefficient index gives the total or largest number of particles involved. VITAL for all sumulations!
Constant kPositiveCoefs = 1; // Set to 1 if you want all the linear coefficeints to be positive for an image/slices fit.
Constant kLogTime = 0; // use log time if 1, linear time if 0.

//Specialized optional parameters for speific experiments (not TA or general streak camera trPL)
Constant kLtMax = 100000e-9 // long-time max. Only used for long-time simulations where you don't want to include ultrafast rate constants at 10s-100s of ns in time.
Constant kLtoffset = 50e-9 // time at which to splice the long-and short-time simulations. Needs to be equal to kTMax for these hybrid timescale simulations to work.
strConstant ksTransLocs = "1;2;3;" // write down which species (can be more than one), by number, you want to contribute to transients (only used for TRMC, TRPL, etc.).
Constant kMuCoef = 8; // which coefficeint gives the carrier mobility? (only used for TRMC).
Constant kCavTime = 10e-9; // what is the TRMC cavity response time? (Useful for TRMC and TREPR)
Constant kApplyCavResp = 1; // if this is set to 1 then all trajectories will be convolved with an exponential response function (Useful for TRMC and TREPR)
                                                                                // in the image fit code. kCavTime is used as the time constant of that function.
Constant kNormalize = 1;



Function eqn(pw, tt, yw, dydt)// these are the equations to solve. Make sure the names of the parameters match those in the string constants above.

        Wave pw
        Variable tt
        Wave yw
        Wave dydt

        Variable center = PW[%position] // format: localname = PW[%globalname]
        Variable width = PW[%width]
        Variable N0 = PW[%N0] // transient excitation density
        Variable gcr = PW[%gcr]
        Variable g0 = PW[%g0]
        Variable gi = pw[%gi]
        Variable V = pw[%V]
        variable kex = pw[%kex]
        Variable L = pw[%L]
        Variable fc0 = G0
        variable T = pw[%T]
        variable q = 1.602e-19
        variable k = 1.38e-23
        variable mue = pw[%mue]
        variable muh = pw[%muh]
        variable mu = min(mue,muh)
        variable D = mu*k*T/q
        variable Vbi = pw[%Vbi]
        variable transient = pw[%transient];
        variable kdt0 = pw[%kdt0]
        variable Nt = pw[%Nt]
        variable Et = pw[%Et]
        variable injection = pw[%injection]
        variable gnr = pw[%gnr]
        variable cN0 = pw[%cN0] // steady-state flux


        kex = (1/6*D/L^2-mu*(V-VBi)/L^2)*injection
```

```
	pw[%kex] = kex

	variable fc = pw[%fc]
	Gi = Fc0/fc*(-1+exp(V/(k*T/q)))*injection
	PW[%GI] = Gi
	variable kdt = kdt0*exp(-Et/(k*T/q))
	variable ci = 0

	Variable Pulse = (1/(sqrt(2*pi)*width))*exp(-(((tt-center))/width)^2/2) //Gaussian pulse profile.//Use a unit area gaussian, and multiply by the fluence
	if(transient == 1)
		Gi = 0
		Kex = 0
		ci = 0
	elseif(transient == 2)
		Gi = 0
		Kex = 0
		ci = 1
	elseif(transient < 1)
		pulse = 0
		ci = 1
	Endif

	dydt[0] = ci*cN0 + N0*pulse+G0-(gcr+gnr)*yw[0]*yw[1]-kex*yw[0]-gcr*(Nt-yw[2])*yw[0]+kdt*yw[2] //- kn*yw[1] //holes
	dydt[1] = ci*cN0 + N0*pulse+G0-(gcr+gnr)*yw[0]*yw[1]-kex*yw[0]-(gnr)*yw[2]*yw[1] //electrons
	dydt[2] = gcr*(Nt-yw[2])*yw[0]-kdt*yw[2]-(gnr)*yw[2]*yw[1] // trapped holes


End

Function CalcJV([Eg]) // solve the ODEs as a function of voltage.
	Variable Eg
	DFREF MDFR=$ksMDFR
	Wave pw = MDFR:ParameterVals		// The rate constants and other wave-defining coefficients go here
	Wave xw= MDFR:Time_Base// This is the time base for the transients, but might not be used.
	DFREF MDFR =$ksMDFR
	Wave species = MDFR:Species
	Species = 0;
	Wave Transfer = MDFR:Transfer
	Wave Time_Base = MDFR:Time_Base
	Variable NEqn = itemsinlist(ksEqnNames)
	variable timerid = startmstimer

	if(paramisdefault(Eg))
		Eg = pw[%Eg]
	endif

	variable dEg = pw[%dEg]
	variable NV = 100
	Variable Vf = Eg
	variable VBi = Eg*0.8
	Make/O/N=(NV) MDFR:JV
	WAVE JV = MDFR:JV
	JV = 0
	setscale/I x, 0, Vf, JV
	variable i =0
```

```
	variable Npnts = dimsize(species,0)
	variable JS = 0
	variable Ji = 0
	variable L = pw[%L]
	variable q = 1.602e-19
	variable t=0
	variable OD = pw[%abs]*L*1e7 // convert cm to nm. abs is in OD/nm
	variable FA = 1-10^(-OD)
	Variable G0 = real(BB_solar_flux(Eg-dEg,300,Pi))/100^2/L*FA // calculate G0 based on the generation rate from absorbing blackbody radiation
	Variable cN0 = real(BB_solar_flux(Eg,kSun_T,ksun_str*0.685))/100^2/L*FA // calculate N0 based on the generation rate from absorbing sunlight.
	Variable Ptot = imag(BB_solar_flux(0.01,kSun_T,ksun_str*0.685))/100^2
	pw[%cN0] = cN0
	pw[%G0] = G0
	pw[%VBi] = VBi

	Do
		pw[%V] = pnt2x(JV,I)
		//PRINT PW[%V]
		SolveODE(Time_base, "eqn", pw, species)
		//print stopmstimer(timerID)/1e6
		t=stopmstimer(timerID)/1e6
		//JV[i] = pw[%G0] + pw[%N0]-pw[%Gi]//-species[npnts-1][1]^2*pw[%gcr]+pw[%G0] + pw[%N0]
		Js = species[npnts-1][0]*pw[%kex]
		Ji = pw[%Gi]//species[npnts-1][0]*species[npnts-1][1]*pw[%gcr] + species[npnts-1][0]*species[npnts-1][1]*pw[%gnr] + (pw[%gcr]+pw[%gnr])*species[npnts-1][2
		if(js>pw[%cN0])
			Js = pw[%cN0]
		endif

		if(Ji > pw[%Gi])
			Ji = pw[%Gi]
		endif

		doupdate
		//JV[i] = (pw[%G0] - pw[%Gi] + Js)*L*q//species[npnts-1][1]*pw[%kex]
		JV[i] = (Js-Ji)*L*q
		if(JV[i] < -40e-3)
			break
		endif

		if(i > 1 && JV[i]*pnt2x(JV,i) < JV[i-1]*pnt2x(JV,i-1))
			//break
		endif
		i+=1
	While(i<nV)
	duplicate/O JV MDFR:PV
	WAVE PV = MDFR:PV
	PV[] = JV[p]*pnt2x(JV,p)
	wavestats/Q PV
	return V_max/ptot

End

Function PCE_vs_Eg()
	variable EgL = 0.1
	variable EGH = 3
	variable dEG = 0.2
```

```
        DFREF MDFR = $ksMDFR
        Variable N = ceil((EgH-Egl)/deg)
        Make/O/N=(N) MDFR:PCE_v_Eg
        WAVE PCE_v_Eg = MDFR:PCE_v_Eg
        setscale/P x, EGL, dEG, PCE_v_Eg
        PCE_v_Eg = CalcJV(Eg = x)

End

constant ksun_str = 6.85e-5 // solid angle subtended by the sun at the earths surface (str)
constant ksun_T = 6000 // temperature of the sun (K)
constant kh = 6.626e-34 // J-s
constant kc = 2.998e8 // m/s speed of light
constant kb = 1.38e-23 // boltzmann
constant kq = 1.602e-19 // electron charge

Function Blackbody(T,Lam)
        Variable T // temperature of body in K
        variable Lam // wavelength in nm at which an intenisty is requested
        variable dl=1 // 1 nm of bandwidth
        variable str = 1 // 1 str solid angle subtended

        Variable n = 1 // index of refraction of medium (air?)

        Variable/D E = kh*kc/(lam*1e-9) // photon energy in J
        variable/D dE = E-kh*kc/((lam+dl)*1e-9) // energy interval

        variable/D f = 2/kh^3*n^2/kc^2*E^2*exp(-E/(kb*T))*dE*str

        Return f

End

Function/C BB_solar_flux(Eg,T,str)
        Variable Eg // gap of semicondcutor
        variable T // temperature of body
        variable str // solid angle subtended
        Variable lam_res = 1 // nm
        Variable lam_max = 190 // nm
        variable lam = kh*kc/(Eg*kq)*1e9
        variable/D flux = 0
        variable P
        Do
                flux += Blackbody(T,Lam)*lam_res
                p+= Blackbody(T,Lam)*lam_res*kh*kc/(lam*1e-9)
                lam -= lam_res
                if(lam < lam_max)
                        break
                Endif

        While(1)
        variable/C flux_power = cmplx(flux*str, P*str)
        return flux_power
end

Function sigmoidn(h,xx,n)
```

```
        Variable h,xx,n

        return h^n/(xx^n+h^n)
end

Function sigmoidp(h,xx,n)
        Variable h,xx,n

        return xx^n/(xx^n+h^n)
end

Function saturation(m, s, xx, n,b)
        variable m, s, xx, n,b
        variable h
        h = s/m
        return (M*xx+b)*sigmoidn(h,xx,n) + sigmoidp(h*1.030,xx,n)*s
End

function/T calcTRMCTrans()

        DFREF MDFR = $ksMDFR
        Wave pw = MDFR:ParameterVals
        WAVE species = MDFR:species
        Variable N = dimsize(species,0)
        make/O/N=(N) MDFR:TRMC
        WAVE TRMC = MDFR:TRMC
        WAVE time_base = MDFR:time_base
        variable pulse_time = pw[%position]
        FindLevel/Q/P Time_Base, pulse_time
        variable pulse_point = floor(V_levelX*0.75)
        TRMC[] = (species[p][0]-species[pulse_point][0])*pw[%muh]+(species[p][1]-species[pulse_point][1])*pw[%mue]

        Return GetWavesDataFolder(TRMC,2)
End

function/T calcTRPLTrans()

        DFREF MDFR = $ksMDFR
        Wave pw = MDFR:ParameterVals
        WAVE species = MDFR:species
        WAVE Time_Base = MDFR:Time_Base
        Variable N = dimsize(species,0)
        make/O/N=(N) MDFR:TRPL
        WAVE TRPL = MDFR:TRPL
        variable pulse_time = pw[%position]
        FindLevel/Q/P Time_Base, pulse_time
        variable pulse_point = floor(V_levelX*0.75)

        TRPL = species[p][0]*species[p][1]*pw[%gcr] - species[pulse_point][0]*species[pulse_point][1]*pw[%gcr]

        Return GetWavesDataFolder(TRPL,2)
End

Function CalcHalfLife(inwave,time_base)
        WAVE inwave, Time_base
        wavestats/Q inwave
```

```
        variable peakloc = V_maxloc
        variable peak = v_max
        Variable N = dimsize(inwave,0)-1
        findLevel/Q/P/R=(peakloc,N) inwave, peak/2
        return time_base[V_levelX]-time_base[peakloc]
End

Function CalculateNife(transient, timebase,N)
        WAVE transient, timebase
        variable N // number to divide by.
        wavestats/Q transient
        Variable NTimes = dimsize(timebase,0)
        duplicate/O transient linTransient
        setscale/I x, timebase[0], timebase[NTimes-1], LinTransient
        LinTransient = interp(x, timebase, transient) // interpolate to a linear scaled wave so we won't get digital results from the half life search.
        variable t0 = timebase[V_maxloc]
        //findlevel/Q/P/Edge=2 transient, V_max/N;
        findlevel/Q/Edge=2 Lintransient, V_max/N;
        //Variable NTimes = dimsize(timebase,0)
        variable t = timebase[NTimes-1]

        if(!V_flag)
                //t = timebase[V_levelx]-t0
                t = V_levelx - t0
        endif

        return t
end

//Function calculate



Function RunModel() // run the model to calculate ERE, SSMC, TRPL, TRMC, and JV data.
        DFREF MDFR = $ksMDFR
        Wave pw = MDFR:ParameterVals

        // set the parameters to run a steady-state solution with no injection
        pw[%transient] = 0
        pw[%solver] = 3
        pw[%kex] = 0
        pw[%gi] = 0
        pw[%injection] = 0

        pw[%e] = 0 // set initial values to zero.
        pw[%h] = 0 //
        pw[%ht] = 0

        // generate local variables from the prameter wave
        variable L = pw[%L] // film thickness
        variable Eg = pw[%Eg] // optical gap
        variable dEg = pw[%dEg] // extra VOC deficiency
        variable OD = pw[%abs]*L*1e7 // convert cm to nm. abs is in OD/nm
        variable FA = 1-10^(-OD) // calculate fraction of light absorbed
        Variable G0 = real(BB_solar_flux(Eg-dEg,300,Pi))/100^2/L*FA // calculate G0 based on the generation rate from absorbing blackbody radiation
        Variable cN0 = real(BB_solar_flux(Eg,kSun_T,ksun_str*0.685))/100^2/L*FA // calculate N0 based on the generation rate from absorbing sunlight.
```

```
Variable Ptot = imag(BB_solar_flux(0.01,kSun_T,ksun_str*0.685))/100^2 // calculate total power incident from the sun (blackbody @ 6000 K)
variable Neq = sqrt(G0/pw[%gcr])
Variable fSolarAbs = cN0/(real(BB_solar_flux(0.05,kSun_T,ksun_str*0.685))/100^2/L*FA)
pw[%cN0] = 0
pw[%G0] = G0
pw[%e] = 0+pw[%Ne] // calculate the equilibrium carrier density due to blackbody generation, and add any doping to it.
pw[%h] = 0
pw[%ht] = 0
pw[%solver] = 1
WAVE species = $TestEqn() // run the model in the dark to find the steady-state charge densities.
doupdate
Variable N = dimsize(species,0)-1
pw[%cN0] = cN0 // set the injection density from illumination
variable ebb = species[N][1] // use the equilibrium carrier density found due to blackbody generation.
variable hbb = species[N][0] //
variable htbb = species[N][2]
pw[%e] = species[N][1] // use the equilibrium carrier density found due to blackbody generation.
pw[%h] = species[N][0] //
pw[%ht] = species[N][2]
Wave species = $TestEqn() // run the model to calculate steady-state trajectories.

variable h0 = species[N][0] // store the steady-state densities found for white light illumination
variable e0 = species[N][1]
variable ht0 = species[N][2]

variable ERE = species[N][0]*species[N][1]*pw[%gcr]/pw[%cN0] // calculate ERE as the fraction of incident light that is re-emmited.
variable SSMC = ((species[N][0]-species[0][0])*pw[%muh]+(species[N][1]-species[0][1])*pw[%mue])*kq // calculate SSMC as a steady-state photoconductivity in S/cm
pw[%transient] = 0 // set transient = 0 for steady-state simulation
pw[%solver] = 3 // set solver = 3 for faster steady-state simulations
pw[%injection] = 1 // set injection = 1 to enable calculation of injection currents
pw[%fc] = ERE // use the simulated ERE value to set the fraction of recombination that is radiative for purposes of JV modeling
Variable PCE = CalcJV() // calculate a JV curve, and return the power conversion efficiency
//print pw[%N0]

pw[%transient] = 1 // setup for transient solutions, without white light bias.
pw[%solver] = 1 // use solver = 1 for modeling transients
pw[%N0] = 1e16 // set the laser pulse fluence in photons/cm^2
pw[%injection] = 0



// set the initial densities equal to the results found for steady-state in the dark
pw[%h] = hbb
pw[%e] = ebb
pw[%ht] = htbb

Wave species = $TestEqn() // run the model for transient trajectories
WAVE TRMC = $CalcTRMCTrans() // calculate a TRMC transient from it in yield-mobility product
WAVE TRPL = $CalcTRPLTrans() // calculating a PL transient from it in counts.

duplicate/O TRMC MDFR:TRMC_w
WAVE TRMC_w = MDFR:TRMC_w
duplicate/O TRPL MDFR:TRPL_w
WAVE TRPL_w = MDFR:TRPL_w
TRPL_w = sqrt(abs(TRPL))
TRMC_w = sqrt(abs(TRMC))
```

```
doupdate
TRMC /= pw[%N0]
TRPL /= pw[%N0]
WAVE TimeBase = MDFR:time_base
variable TRMC_tau2 = CalculateNife(TRMC, timebase, 2)
variable TRPL_tau2 = CalculateNife(TRPL, timebase, 2)
Variable TRMC_tau10 = CalculateNife(TRMC, timebase, 10)
variable TRPL_tau10 = CalculateNife(TRPL, timebase, 10)
wavestats/Q TRMC
Variable TRMC_peak = v_max

// fit the TRMC and TRPL data to get those lifetime numbers
//Variable V_FitError, V_FitQuitReason
//CurveFit/N/Q dblexp_XOffset TRPL[65,26000] /X=timebase /D/W=TRPL_w/I=1
//WAVE w_coef
//variable TRPL_avg_tau = (w_coef[1]*w_coef[2]^2+w_coef[3]*w_coef[4]^2)/(w_coef[1]*w_coef[2]+w_coef[3]*w_coef[4])
//if(V_fitQuitReason != 0)
//        TRPL_avg_tau = NAN
//Endif

variable TRPL_avg_tau = FitTransients(TRPL, timebase, TRPL_w, 1)

//CurveFit/N/Q dblexp_XOffset TRMC[65,26000] /X=timebase /D /W=TRMC_w/I=1
//variable TRMC_avg_tau = (w_coef[1]*w_coef[2]+w_coef[3]*w_coef[4])/(w_coef[1]+w_coef[3])
//if(V_fitQuitReason != 0)
//        TRMC_avg_tau = NAN
//Endif

variable TRMC_avg_tau = FitTransients(TRMC, timebase, TRMC_w, 0)


pw[%transient] = 2 // setup for transient solutions, with white light bias.
pw[%solver] = 1 // use solver = 1 for modeling transients
pw[%N0] = 1e16 // set the laser pulse fluence in photons/cm^2

// set the initial densities equal to the results found for steady-state illumination for white light biased transients
pw[%h] = h0
pw[%e] = e0
pw[%ht] = ht0

Wave species = $TestEqn() // run the model for transient trajectories
WAVE TRMC = $CalcTRMCTrans() // calculate a TRMC transient from it in yield-mobility product
WAVE TRPL = $CalcTRPLTrans() // calculating a PL transient from it in counts.

duplicate/O TRMC MDFR:TRMC_w
WAVE TRMC_w = MDFR:TRMC_w
duplicate/O TRPL MDFR:TRPL_w
WAVE TRPL_w = MDFR:TRPL_w
TRPL_w = sqrt(abs(TRPL))
TRMC_w = sqrt(abs(TRMC))

doupdate
TRMC /= pw[%N0]
TRPL /= pw[%N0]
WAVE TimeBase = MDFR:time_base
```

```
variable TRMC_tau2_WL = CalculateNife(TRMC, timebase, 2)
variable TRPL_tau2_WL = CalculateNife(TRPL, timebase, 2)
Variable TRMC_tau10_WL = CalculateNife(TRMC, timebase, 10)
variable TRPL_tau10_WL = CalculateNife(TRPL, timebase, 10)
wavestats/Q TRMC
Variable TRMC_peak_WL = v_max

// fit the TRMC and TRPL data to get those lifetime numbers
//CurveFit/N/Q dblexp_XOffset TRPL[65,26000] /X=timebase /D /W=TRPL_w/I=1
//variable TRPL_avg_tau_WL = (w_coef[1]*w_coef[2]^2+w_coef[3]*w_coef[4]^2)/(w_coef[1]*w_coef[2]+w_coef[3]*w_coef[4])
//if(V_fitQuitReason != 0)
//        TRPL_avg_tau_WL = NAN
//Endif

variable TRPL_avg_tau_WL = FitTransients(TRPL, timebase, TRPL_w, 1)

//CurveFit/N/Q dblexp_XOffset TRMC[65,26000] /X=timebase /D /W=TRMC_w/I=1
//variable TRMC_avg_tau_WL = (w_coef[1]*w_coef[2]+w_coef[3]*w_coef[4])/(w_coef[1]+w_coef[3])
//if(V_fitQuitReason != 0)
//        TRMC_avg_tau_WL = NAN
//Endif

variable TRMC_avg_tau_WL = FitTransients(TRMC, timebase, TRMC_w, 0)


// print results
print "power conversion efficiency: " +  num2str(PCE*100) + "%"
print "steady-state fSmt: " + num2str(SSMC)
print "external radiative efficiency: " + num2str(ERE*100) + "%"
print "TRPL half-life: " + num2str(TRPL_tau2_WL) + " s"
print "TRPL avg Tau: " + num2str(TRPL_avg_tau_WL) + " s"
print "TRMC half-life: " + num2str(TRMC_tau2_WL) + " s"
print "TRMC avg tau: " + num2str(TRMC_avg_tau_WL) + " s"
print "TRMC fSm: " + num2str(TRMC_peak) + " cm^2/V/s"

WAVE measurements = MDFR:Measurements
if(!waveexists(measurements))
        Make/O/N=17 MDFR:Measurements
        WAVE measurements = MDFR:Measurements
        setDimLabel 0, 0, PCE, measurements
        setDimLabel 0, 1, SSMC, measurements
        setDimLabel 0, 2, ERE, measurements
        setDimLabel 0, 3, TRPL_2, measurements
        setdimlabel 0, 4, TRPL_aTau, measurements
        setDimLabel 0, 5, TRMC_2, measurements
        setdimlabel 0, 6, TRMC_aTau, measurements
        setDimLabel 0, 7, fsm, measurements
        setdimlabel 0, 8, Eg, measurements
        setDimLabel 0, 9, fSolarAbs, measurements
        setdimlabel 0, 10, L, measurements
        setDimlabel 0, 11, Ne, measurements
        setDimlabel 0, 12, TRPL_2_WL, measurements
        setDimlabel 0, 13, TRPL_aTau_WL, measurements
        setDimlabel 0, 14, TRMC_2_WL, measurements
        setDimlabel 0, 15, TRMC_aTau_WL, measurements
        setDimlabel 0, 16, fsm_WL, measurements
```

```
        endif

        measurements[%PCE] = PCE*100
        measurements[%ERE] = ERE*100
        measurements[%SSMC] = SSMC
        measurements[%TRPL_2] = TRPL_tau2
        measurements[%TRPL_aTau] = TRPL_avg_tau
        measurements[%TRMC_2] = TRMC_tau2
        measurements[%TRMC_aTau] = TRMC_avg_tau
        measurements[%fsm] = TRMC_peak
        measurements[%Eg] = Eg
        measurements[%fSolarAbs] = fSolarAbs
        measurements[%L] = L
        measurements[%Ne] = pw[%Ne]
        measurements[%TRPL_2_WL] = TRPL_tau2_WL
        measurements[%TRPL_aTau_WL] = TRPL_avg_tau_WL
        measurements[%TRMC_2_WL] = TRMC_tau2_WL
        measurements[%TRMC_aTau_WL] = TRMC_avg_tau_WL
        measurements[%fsm_WL] = TRMC_peak_WL



End

Function FitTransients(transient, timebase, weight, PLorMC)
        wave transient, timebase, weight
        variable PLorMc

        CurveFit/N/Q dblexp_XOffset transient[65,26000] /X=timebase /D /W=weight/I=1
        wave w_coef = w_coef
        variable atau = nan
        variable V_fitQuitReason, V_FitError
        if(PLorMC)
                atau = (w_coef[1]*w_coef[2]^2+w_coef[3]*w_coef[4]^2)/(w_coef[1]*w_coef[2]+w_coef[3]*w_coef[4])
        else
                atau = (w_coef[1]*w_coef[2]+w_coef[3]*w_coef[4])/(w_coef[1]+w_coef[3])
        endif

        if(V_fitQuitReason != 0)
                CurveFit/N/Q exp_XOffset transient[65,26000] /X=timebase /D /W=weight/I=1
                aTau = w_coef[2]
                //aTau = NAN
                if(V_fitQuitReason !=0)
                        aTau = NAN
                endif
        Endif
        return atau
end

// generate a random parameter exponentially distributed between a low and a high limit

Function RandomParameter(LowLimit, HighLimit)
        variable lowlimit, highlimit
        variable ran = (-enoise(0.5)+0.5)
        variable logLow = log(lowlimit)
        variable logHigh = log(highLimit)
```

```
	Variable LogRange = loghigh-loglow
	variable RanExp = LogRange*ran + logLow
	variable RanParam = 10^RanExp
	return ranParam
End

Function SetRandomParams()
	//string paramName
	DFREF MDFR = $ksMDFR
	Wave pw = MDFR:ParameterVals
	WAVE XSA = MDFR:XSA
	SetDimLabelsFromList(ksEqnNAmes+KsParameters, XSA)
	WAVE ParamNames = MDFR:ParameterNames

	pw[%mue] = RandomParameter(XSA[%Mue][0], XSA[%Mue][1])
	variable mue = pw[%mue]

	// make it possible to constrain the hole mobility to either be identical to, or within 10 x of the electron mobility
	if(XSA[%Muh][0] == 0 & XSA[%Muh][1] == 0)
		pw[%muh] = Mue
	elseif(XSA[%Muh][0] == 0)
		pw[%Muh] = RandomParameter(mue*0.1, Mue*10)
	else
		pw[%Muh] = RandomParameter(XSA[%Muh][0], XSA[%Muh][1])
	endif


	pw[%gcr] = RandomParameter(XSA[%gcr][0], XSA[%gcr][1])
	pw[%gnr] = RandomParameter(XSA[%gnr][0], XSA[%gnr][1])
	pw[%Nt] = RandomParameter(XSA[%Nt][0], XSA[%Nt][1])
	pw[%Ne] = RandomParameter(XSA[%Ne][0], XSA[%Ne][1])
	pw[%Eg] = RandomParameter(XSA[%Eg][0], XSA[%Eg][1])
End

Function RunRanSim(N)
	Variable N
	DFREF MDFR = $ksMDFR
	Wave AllParams = MDFR:AllParams
	SetRandomParams()
	RunModel()
	WAVE params = MDFR:ParameterVals
	WAVE results = MDFR:Measurements
	Variable NParams = dimsize(params,0)
	Variable NResults = dimsize(results,0)

	WAVE AllParams = MDFR:AllParams
	if(!waveexists(AllParams))
		Duplicate/O params MDFR:AllParams
		WAVE AllParams = MDFR:AllParams
		//AllParams = params
	endif

	WAVE AllResults = MDFR:AllResults
	if(!waveexists(AllResults))
		duplicate/O results MDFR:allResults
		WAVE AllResults = MDFR:AllResults
```

```
                //AllResults = params
        endif

        variable i = 0

        variable TotTime = 0
        variable estimatedtime = 0
        Do
                variable timer = startmstimer
                SetRandomParams()
                RunModel()
                concatenate {params}, allParams
                concatenate {results}, allresults
                TotTime +=  stopmstimer(timer)
                estimatedtime = totTime/i*N
                print "iteration " + num2str(i) + " of " + num2str(N)
                print "elapsed time: " + num2str(TotTime*1e-6/60) + " minutes"
                print "estimated time: " + num2str(estimatedTime*1e-6/60) + " minutes"
                i += 1
        While(i<N)

end

Function RunRanSimSet(N)
        Variable N
        DFREF MDFR = $ksMDFR
        WAVE XSA = MDFR:XSA


        // first setup for a brodaly varying simulation:
        XSA[%Eg][0] = 0.75
        XSA[%Eg][1] = 3
        XSA[%Mue][0] = 0.001
        XSA[%Mue][1] = 1
        XSA[%Muh][0] = 0.001
        XSA[%Muh][1] = 1
        XSA[%gcr][0] = 1e-11
        XSA[%gcr][1] = 1e-9
        XSA[%gnr][0] = 1e-12
        XSA[%gnr][1] = 1e-8
        XSA[%Nt][0] = 1e15
        XSA[%Nt][1] = 1e18
        XSA[%Ne][0] = 1e15
        XSA[%Ne][1] = 1e18

        //RunRanSim(N)

        // save these results and reset the results waves
        WAVE allResults = MDFR:AllResults
        WAVE AllParams = MDFR:AllParams
        Duplicate/O AllResults MDFR:AllResults_0
        Duplicate/O AllParams MDFR:AllParams_0
        Killwaves/Z AllResults
        KillWaves/Z AllParams

        // next make energy gap constant
```

```
XSA[%Eg][0] = 1.4
XSA[%Eg][1] = 1.4
//RunRanSim(N)

// save these results and reset the results waves
WAVE allResults = MDFR:AllResults
WAVE AllParams = MDFR:AllParams
Duplicate/O AllResults MDFR:AllResults_1
Duplicate/O AllParams MDFR:AllParams_1
Killwaves/Z AllResults
KillWaves/Z AllParams

// next narrow down to mobility ratios within 10 x
XSA[%Muh][0] = 0
XSA[%Muh][1] = 1
//RunRanSim(N)
// save these results and reset the results waves
WAVE allResults = MDFR:AllResults
WAVE AllParams = MDFR:AllParams
Duplicate/O AllResults MDFR:AllResults_2
Duplicate/O AllParams MDFR:AllParams_2
Killwaves/Z AllResults
KillWaves/Z AllParams

//then mobility ratios = 1
XSA[%Muh][0] = 0
XSA[%Muh][1] = 0
//RunRanSim(N)
// save these results and reset the results waves
WAVE allResults = MDFR:AllResults
WAVE AllParams = MDFR:AllParams
Duplicate/O AllResults MDFR:AllResults_3
Duplicate/O AllParams MDFR:AllParams_3
Killwaves/Z AllResults
KillWaves/Z AllParams

// then gcr is constant
XSA[%gcr][0] = 1e-10
XSA[%gcr][1] = 1e-10
//RunRanSim(N)
// save these results and reset the results waves
WAVE allResults = MDFR:AllResults
WAVE AllParams = MDFR:AllParams
Duplicate/O AllResults MDFR:AllResults_4
Duplicate/O AllParams MDFR:AllParams_4
Killwaves/Z AllResults
KillWaves/Z AllParams

// then Mu is constant good solar material with good transport.
XSA[%mue][0] = 1
XSA[%mue][1] = 1
//RunRanSim(N)
// save these results and reset the results waves
WAVE allResults = MDFR:AllResults
WAVE AllParams = MDFR:AllParams
Duplicate/O AllResults MDFR:AllResults_5
```

```
        Duplicate/O AllParams MDFR:AllParams_5
        Killwaves/Z AllResults
        KillWaves/Z AllParams

        // then Mu is random but larger: Good solar material with good transport
        XSA[%mue][0] = 0.5
        XSA[%mue][1] = 5
        RunRanSim(N)
        // save these results and reset the results waves
        WAVE allResults = MDFR:AllResults
        WAVE AllParams = MDFR:AllParams
        Duplicate/O AllResults MDFR:AllResults_6
        Duplicate/O AllParams MDFR:AllParams_6
        Killwaves/Z AllResults
        KillWaves/Z AllParams

end

Function OR_TriExp_IRF_Gauss(pw, yw, xw) : FitFunc

        //CurveFitDialog/ These comments were created by the Curve Fitting dialog. Altering them will
        //CurveFitDialog/ make the function less convenient to work with in the Curve Fitting dialog.
        //CurveFitDialog/ Independent Variables 1
        //CurveFitDialog/ x
        //CurveFitDialog/ Coefficients 11
        //CurveFitDialog/ w[0] = Pos
        //CurveFitDialog/ w[1] = irf_w
        //CurveFitDialog/ w[2] = A1
        //CurveFitDialog/ w[3] = t1
        //CurveFitDialog/ w[4] = A2
        //CurveFitDialog/ w[5] = t2
        //CurveFitDialog/ w[6] = A3
        //CurveFitDialog/ w[7] = t3

        Wave pw, yw, xw
        //SVAR ADFRs = root:OR_ADFR;
        //SVAR MDFRs = root:OR_MDFR;
        //DFREF  ADFR = $ADFRs;
        DFREF MDFR = $ksMDFR;
        variable N=500
        Wavestats/Q xw
        Make/D/O/N=(N) MDFR:Pulse, MDFR:Pulse2, MDFR:TriExp         //Make the fitting waves in the master data folder (MDF)
        WAVE Pulse = MDFR:Pulse;
        WAVE Pulse2 = MDFR:Pulse2;
        WAVE TriExp = MDFR:TriExp;                                        //Declare wave references to the newly made waves
        Variable dx = (V_max-V_Min)/N;
        SetScale/I X, V_min, V_max, Pulse;
        SetScale/I X, V_min, V_max, Pulse2;

        SetScale/I X, V_min, V_max, TriExp;



        //Variable B=22.9/10.2
        //Variable qe=1.602176487e-19
        //Variable i = 0;                                          // Decalre important constants
        //Variable Ni = dimsize(xw, 0)
```

```
        Pulse = exp(-(x-pw[0])^2/(2*pw[1]^2))/(sqrt(2*pi)*pw[1]);
        //Pulse = exp(-x/pw[2]);
        //convolve pulse2, pulse;
        redimension/N=(N) pulse;
        WaveStats/Q Pulse                                                                       // this and the next l
         Pulse/=V_Sum

         Triexp=pw[2]*exp(-x/pw[3]) + pw[4]*exp(-x/pw[5]) + pw[6]*exp(-x/pw[7])



         Duplicate/O Triexp MDFR:Triexp_temp
         WAVE TriExp_TEmp = MDFR:TriExp_Temp;
        Convolve Pulse, Triexp_temp
        Redimension/N=(N) Triexp_temp
        //Triexp_temp*=B*qe*pw[3]*pw[4]//1E18
        yw = Triexp_temp(xw[p])

End


function graphstyle()
        ModifyGraph log=1;
        ModifyGraph mode=2,lsize=1.5
        ModifyGraph mirror=1,fStyle=1,fSize=12,axThick=1.5
        ModifyGraph width=360,height=360
        ModifyGraph tick(bottom)=2
        ModifyGraph tick=2
end

Function DisplayAllResults()
        WAVE AR = root:packages:OR_Functions:DiffEq:allResults_6//root:packages:OR_Functions:DiffEq:allResults

        Variable N = dimsize(AR,1)
        if(N> 100)
                matrixtranspose AR
        endif

        Display AR[][0] vs AR[][1];
        Label left "PCE (%)";DelayUpdate;Label bottom "SSMC (S/cm)";
        graphstyle()

        Display AR[][0] vs AR[][2];
        Label left "PCE (%)";DelayUpdate;Label bottom "ERE (%)";
        graphstyle()

        Display AR[][0] vs AR[][3];
        Label left "PCE (%)";DelayUpdate;Label bottom "TRPL 1/2-life (s)";
        graphstyle()

        Display AR[][0] vs AR[][4];
        Label left "PCE (%)";DelayUpdate;Label bottom "TRPL avg tau (s)";
        graphstyle()

        Display AR[][0] vs AR[][5];
```

```
        Label left "PCE (%)";DelayUpdate;Label bottom "TRMC 1/2-life (s)";
        graphstyle()

        Display AR[][0] vs AR[][6];
        Label left "PCE (%)";DelayUpdate;Label bottom "TRMC avg tau (s)";
        graphstyle()

        Display AR[][0] vs AR[][7];
        Label left "PCE (%)";DelayUpdate;Label bottom "TRMC fSm (cm\S2\MV\S-1\Ms\S-1\M)";
        graphstyle()

        Display AR[][0] vs AR[][11];
        Label left "PCE (%)";DelayUpdate;Label bottom "doping density (cm\S-3\M)";
        graphstyle()

        Display AR[][0] vs AR[][12];
        Label left "PCE (%)";DelayUpdate;Label bottom "WL Bias TRPL 1/2-life (s)";
        graphstyle()

        Display AR[][0] vs AR[][13];
        Label left "PCE (%)";DelayUpdate;Label bottom "WL Bias TRPL avg tau (s)";
        graphstyle()

        Display AR[][0] vs AR[][14];
        Label left "PCE (%)";DelayUpdate;Label bottom "WL Bias TRMC 1/2-life (s)";
        graphstyle()

        Display AR[][0] vs AR[][15];
        Label left "PCE (%)";DelayUpdate;Label bottom "WL Bias TRMC avg tau (s)";
        graphstyle()

        Display AR[][0] vs AR[][16];
        Label left "PCE (%)";DelayUpdate;Label bottom "WL Bias TRMC fSm (cm\S2\MV\S-1\Ms\S-1\M)";
        graphstyle()

end

Function export()

        Save/J/DLIM=","/U={1,0,0,0} :packages:OR_Functions:DiffEq:AllParams_6 as "AllParams_6.txt"
        Save/J/DLIM=","/U={0,0,1,0} :packages:OR_Functions:DiffEq:AllResults_6 as "AllResults_6.txt"

end
```

3.2 ODE_Solver.ipf

3.3 ODE_Image_Fit.ipf

```
#pragma rtGlobals=3               // Use modern global access method and strict wave access.
// This code relies on the procedure: ODEs_191016
// it uses the ODE solver implemented there to fit transient absorption data.
// This code is designed for use with the .csv files exported from Surface Explorer, Ultrafast Systems
// It could readily be adapted for use with streak camera data for PL analysis.
```

```
Static StrConstant ksFileExt = ".dac" // file extension to look for when loading data.
Static Constant kTimeUnit = 1e-9
Static Constant kMaxGSBWL = 900 //[nm] limit GSB contributions so that they cannot show up red of this edge
Static Constant kGSB_Species = 0 // index of the GSB species

//Static StrConstant ksLDFR = "root:packages:OR_Functions:DiffEq:ImageFit" // path in which to store all the bits and pieces.

Menu "ODE_Image_Fitting" // define some menu buttons for commonly used functions.

	"Load Transient Images",/Q,  LoadImageByKeyPrompt();
	"Plot Selected",/Q,  PlotSelectedMats();
	"Graph Slices at A Cursor/1",/Q, PlotSlicesByACursor();
	"Cut Out Data Between A&B",/Q, RemoveTimeSliceAB();
	"Stitch Selected at WL A",/Q, Print "not added yet";
	"Add Timeslice to Fit at A Cursor/2",/Q, AddSliceToFit();
	"Clear Slices to Fit",/Q, ClearSlicesToFit();
	"Show ODE Guess, Fit to Slices/3",/Q,  print SolveODEAndFitSlices();
	"Fit ODE to Slices",/Q, FitODEToSlices();
	"Fit ODE Solution to Top Image",/Q, FitImageFromMenu();
	"Calculate Parameter Errors",/Q, CalcODEParamErrors();
End

Function AssignAllSpecies(species, Dest, PW, SpeciesSelect) // Copied from the ODE solver and modified. This function allows
// you to copy a specific set of species from the Species wave (result of ODE solver) to create an arbitrary linear combination
// of the individual species.
//Species - the 2D wave that stores the result of an ODE solution
// Dest - a 1D wave where a linear combination of the columns from Species will be stored
// PW - the parameters of the ODE model
// SpeciesSelect - a 1D wave consisting of either 1 or 0, indicating if a given species will be included in the output (1)
	WAVe Species, Dest, PW, SpeciesSelect;
	DFREF MDFR = $ksMDFR;
	Variable N = dimsize(PW,0);
	Variable i = 0;
	Dest = 0;
	Variable col =0;
	//Duplicate/O/R=[][0] Species MDFR:Temp
	//WAVE Temp = MDFR:Temp;

	Do
		//Temp[] = Species[p][i]
		//Wavestats/Q Temp;
		if(SpeciesSelect[i])
			Dest += species[p][i]*pw[i]//V_max
		Endif
		i+=1
	While(i<N)

End


Function FitSpeciesSum(pw, yw, xw) : FitFunc
	//Fit function that uses the species trajectories in a 2D wave to calculate a sum of species and fit it to transient data.
	Wave pw		// The rate constants and other wave-defining coefficients go here
	Wave xw		// This is the time base for the transients, but might not be used.
	Wave yw		// This will contain the simulated decay
```

```
        DFREF MDFR =$ksMDFR
        Wave species = MDFR:NormSpecies
        Wave Time_Base = MDFR:Time_Base
        WAVE SumTrans = MDFR:SumTransfer
        WAVE SpeciesSelect = MDFR:SpeciesSelect
        AssignAllSpecies(species, SumTrans,PW,SpeciesSelect)

        yw = interp(XW[p], Time_Base, SumTrans)  // Very important, as this line enables curvefit routine to function correctly, inclusing the cursors.

End

Function SimulateSpectrum(Species) // Code for testing purposes only. Given a set of ODE solutions, generate a random simulated TA spectrum
        WAVE Species
        Variable E0 = 0.5;
        Variable E1 = 3.5;
        Variable NE = 100;
        Variable NT = dimsize(Species,0)
        Variable EStep = (E1-E0)/NE;
        DFREF MDFR = $ksMDFR;
        Variable NSpecies = dimsize(Species,1);

        MAke/O/N = (Nt,NE)MDFR:SimImageSpec
        WAVE SIS = MDFR:SimImageSpec
        SetScale/P y, E0, EStep, SIS;

        WAVE TB = MDFR:Time_Base
        Variable tStep = TB[1]-TB[0];
        SetScale/P x, 0, tStep, SIS;
        Variable i =0; // iterate through energy points
        Variable j = 0; // iterate through species
        Variable Ec, Ew, Amp;
        Variable E = 0;
        Wavestats/Q Species
        Species/=V_max; // normalize the species transients;
        SIS = 0;
        Do
                Ec = (enoise(1)+1)/2*(E1-E0)+E0 // chose a random center that is inside our simulated wavelength range. This is the center position of the peak
                Ew = (enoise(1)+1)/2+0.1 // chose another random number that gives the width of the peak.
                amp = (enoise(1)+1)/2 // amplitude between 0 and 1;
                print "For Species " + num2str(j) + " w = " + num2str(Ew) + " and c = " + num2str(Ec) + " eV, with amplitude: " +num2str(amp)
                Do
                        E = i*EStep+E0;
                        SIS[][i] += species[p][j]*gauss(E, Ec, Ew)*amp+gnoise(0.01)

                i+=1;
                While(i<NE)

                i=0;
                j+=1;
        While(j<NSpecies)

End

Function/T GetWLSlice(Image, col) // Get a wavelength slice from the TA data. Image is a 2D matrix that is assumed to consist
// of time in the rows and wavelength in the columns
// Image - the 2D input data. note that I'm using a special Igor trick here. This 2D wave should have it's wavelenegth and time waves
```

```
// (1D numeric waves giving those values) specified in it's wave notes using a keyword value paring. See below.
// col - numeric specification of the column to cut out.
// Returns a string with the wave reference to the slice.

	WAVE Image
	Variable Col
	DFREF MDFR = $ksMDFR;
	//DFREF LDFs = GetWavesDataFolder(Image,1)

	Duplicate/O/R=[][col] Image MDFR:WLSlice
	WAVE DS = MDFR:WLSlice;
	REdimension/N=(dimsize(DS,0)) DS

	String xwave_s = GetNote(Image, "WL"); // getting the path to the wavelength values from the wavenote!
	String TruncXWave_s = xwave_s +"s"
	Duplicate/O $XWave_s $TruncXWave_s
	WAVE TXWAVE = $TruncXWave_s
	Deletepoints 0, 1, TXWave
	String NoteString = "WL="+TruncXWave_s;
	Note/K/NOCR DS, NoteString
	Return GetWAvesDataFolder(DS,2)
End

Function/T GetAvgWLSlice(Image, col,Navgs) // Get a wavelength slice from the TA data. Image is a 2D matrix that is assumed to consist
// of time in the rows and wavelength in the columns
// Image - the 2D input data. note that I'm using a special Igor trick here. This 2D wave should have it's wavelenegth and time waves
// (1D numeric waves giving those values) specified in it's wave notes using a keyword value paring. See below.
// col - numeric specification of the column to cut out.
// Returns a string with the wave reference to the slice.

	WAVE Image
	Variable Col
	Variable NAvgs// = 20;
	DFREF MDFR = $ksMDFR;
	//DFREF LDFs = GetWavesDataFolder(Image,1)
	Variable Cols = dimsize(image,1);
	Variable i = 0;


	Duplicate/O/R=[][col] Image MDFR:WLSlice
	WAVE DS = MDFR:WLSlice;
	REdimension/N=(dimsize(DS,0)) DS
	ds = 0;
	Do
		if(i+col>=cols)
			Break
		Endif
		Duplicate/O/R=[][col+i] Image MDFR:WLSliceTemp
		WAVE DSt = MDFR:WLSliceTemp;
		REdimension/N=(dimsize(DS,0)) DSt
		ds += DSt
		i+=1;
	While(i<NAvgs)

	ds/=i;
```

```
        String xwave_s = GetNote(Image, "WL"); // getting the path to the wavelength values from the wavenote!
        String TruncXWave_s = xwave_s +"s"
        Duplicate/O $XWave_s $TruncXWave_s
        WAVE TXWAVE = $TruncXWave_s
        Deletepoints 0, 1, TXWave
        String NoteString = "WL="+TruncXWave_s;
        Note/K/NOCR DS, NoteString
        Return GetWAvesDataFolder(DS,2)
End

Function/T GetTimeSlice(Image, row) // same as GetWLSlice, but for time.

        WAVE Image
        Variable row
        DFREF MDFR = $ksMDFR;
        //DFREF LDFs = GetWavesDataFolder(Image,1)

        Duplicate/O/R=[row][] Image MDFR:TimeSlice
        WAVE DS = MDFR:TimeSlice;
        Matrixtranspose DS
        REdimension/N=(dimsize(DS,0)) DS

        String xwave_s = GetNote(Image, "t");
        String TruncXWave_s = xwave_s +"s"
        Duplicate/O $XWave_s $TruncXWave_s
        WAVE TXWAVE = $TruncXWave_s
        if(dimsize(TxWAve,0)>Dimsize(DS,0))
                Deletepoints 0, 1, TXWave
        Endif
        String NoteString = "t="+TruncXWave_s;
        Note/K/NOCR DS, NoteString
        if(DS[0] ==0)
                FillFirstPoint(DS)
        Endif

        Return GetWAvesDataFolder(DS,2)
End

Function/T GetWeightSlice(Image, row) // same as GetWLSlice, but for time.

        WAVE Image
        Variable row
        DFREF MDFR = $ksMDFR;
        //DFREF LDFs = GetWavesDataFolder(Image,1)

        Duplicate/O/R=[row][] Image MDFR:WeightSlice
        WAVE WS = MDFR:WeightSlice;
        Matrixtranspose WS
        REdimension/N=(dimsize(WS,0)) WS


        Return GetWAvesDataFolder(WS,2)
End

Function/T GetAvgTimeSlice(Image, row, NAvgs) // same as GetWLSlice, but for time.
```

```
        WAVE Image
        Variable row
        Variable NAvgs// = 20;
        DFREF MDFR = $ksMDFR;

        //DFREF LDFs = GetWavesDataFolder(Image,1)
        Variable rows = dimsize(image,0);
        Variable i = 0;

        Duplicate/O/R=[row][] Image MDFR:TimeSlice
        WAVE DS = MDFR:TimeSlice;
        Matrixtranspose DS
        REdimension/N=(dimsize(DS,0)) DS
        DS = 0;

        Do
                if(i+row>= Rows || i+row <0)
                        Break
                Endif

                Duplicate/O/R=[row+i][] Image MDFR:TimeSliceTemp
                WAVE DSt = MDFR:TimeSliceTemp;
                Matrixtranspose DSt
                REdimension/N=(dimsize(DSt,0)) DSt
                DS += DSt
                i+=1;
        While(i<NAvgs)

        ds/=i;

        String xwave_s = GetNote(Image, "t");
        String TruncXWave_s = xwave_s +"s"
        Duplicate/O $XWave_s $TruncXWave_s
        WAVE TXWAVE = $TruncXWave_s
        Deletepoints 0, 1, TXWave
        String NoteString = "t="+TruncXWave_s;
        Note/K/NOCR DS, NoteString
        if(DS[0] ==0)
                FillFirstPoint(DS)
        Endif

        Return GetWAvesDataFolder(DS,2)
End

Function/T GetAvgSlice(Image, row, NAvgs, dim) // same as GetWLSlice, but for time.

        WAVE Image
        Variable row
        Variable NAvgs// = 20;
        //string suffix // string to append to the new wave as "_suffix"
        variable dim // dimension to slice through. 0 = rows, 1 = columns.
        DFREF MDFR = $ksMDFR;

        //DFREF LDFs = GetWavesDataFolder(Image,1)
        Variable rows = dimsize(image,dim);
        Variable i = 0;
```

```
        Variable type = str2num(GetNote(image,"type"))
        String SliceName = "AvgSlice_" + num2str(dim)
        if(dim == 0)
                Duplicate/O/R=[row][] Image MDFR:$SliceName
        Else
                Duplicate/O/R=[][row] Image MDFR:$SliceName
        Endif
        WAVE DS = MDFR:$SliceName

        if(dim == 0)
                Matrixtranspose DS
        Endif

        REdimension/N=(dimsize(DS,0)) DS
        DS = 0;

        Do
                if(i+row>= Rows || i+row <0)
                        Break
                Endif
                if(dim == 0)
                        Duplicate/O/R=[row+i][] Image MDFR:TimeSliceTemp
                Else
                        Duplicate/O/R=[][row+i] Image MDFR:TimeSliceTemp
                Endif

                WAVE DSt = MDFR:TimeSliceTemp;
                if(dim ==0)
                        Matrixtranspose DSt
                Endif
                REdimension/N=(dimsize(DSt,0)) DSt
                DS += DSt
                i+=1;
        While(i<NAvgs)

        ds/=i;

        String kw
        String xwave_s
//        if(type == 1 && dim == 0)
//                kw = "t"
//        Elseif(type == 1 && dim > 0)
//                kw = "WL"
        //Elseif(type > 1 && dim == 0)
//                kw = "WL"
//        Elseif(type > 1 && dim >0)
//                kw = "t"
//        Endif

if(dim == 0)
                kw = "t"
        Elseif(dim > 0)
                kw = "WL"
Endif

        xwave_s = GetNote(Image, kw);
```

```
	String TruncXWave_s = xwave_s +"s"
	Duplicate/O $XWave_s $TruncXWave_s
	WAVE TXWAVE = $TruncXWave_s
	Deletepoints 0, 1, TXWave

	String NoteString = kw+"="+TruncXWave_s+",type="+num2str(type);
	Note/K/NOCR DS, NoteString
	if(DS[0] ==0)
		FillFirstPoint(DS)
	Endif

	Return GetWAvesDataFolder(DS,2)
End

Function FillFirstPoint(Inwave)
	WAVE InWave
	Duplicate/O/R=[1,4] Inwave TempWave
	CurveFit/N/W=2 line Tempwave
	WAVE w_coef
	//print w_coef
	Inwave[0] = w_coef[0]
	//print inwave;
	killwaves/Z tempwave;
End

Function/T FitSlice(slice,Species, Coef, eps, SpeciesSelect, [weight]) // Fit that slice!
	// Slice - a 1D wave containing a time slice
	// Species - the results of an ODE solution
	// Coef - the linear coeffcients to describing fitting the ODE solution to the data. This is NOT the ODE model parameters
	// eps - epsilon wave to go with coef
	//SpeciesSelect - a 1D wave with the same length as the number of columns in Species. A 0 indicates that that species
	// shall not be included in the linear combination.
	// Returns The result of the fit as a string wave reference.

	WAVE Slice, Species, Coef,eps,SpeciesSelect;
	WAVE weight
	Variable weighted = 1;
	if(paramisdefault(weight))
		weighted = 0
	Endif

	DFREF MDFR = $ksMDFR;
	WAVE SumTrans = MDFR:SumTransfer;
	//Variable V_fitoptions = 4 // supress progress window
	Variable V_FitError = 0
	Wavestats/Q Slice
	WAVE timewave = $getnote(slice, "t");
	Variable Ncoef = dimsize(coef,0)
	Coef = V_max/NCoef; // set the initial guess.
	WAVE Constraints = $MakePositiveConstraints(Coef,SpeciesSelect) // constrain all the linear coeffcients to be positive
	String HoldStr = MakeHoldStr(SpeciesSelect) // you have to hold coeffcients for species that do not contribute
	 ZeroGuessIfHeld(HoldStr,Coef) // the initial guess for held coeffcients has to be zero in this case

	If(weighted)
		if(WaveExists(timewave))
```

```
				if(waveexists(constraints))
					FuncFit/N=1/NTHR=0/Q/H=HoldStr FitSpeciesSum coef  slice/X=timewave /D/E=eps/C=Constraints/I=1/W=weight
				else
					FuncFit/N/Q/H=HoldStr/W=2 FitSpeciesSum coef  slice/X=timewave /D/E=eps/I=1/W=weight
				Endif
			Else
				if(WaveExists(Constraints))
					FuncFit/N=1/NTHR=0/Q/H=HoldStr FitSpeciesSum coef  slice /D/E=eps/C=Constraints/I=1/W=weight
				Else
					FuncFit/N=1/NTHR=0/Q/H=HoldStr FitSpeciesSum coef  slice /D/E=eps/I=1/W=weight
				Endif
			Endif

		Else
			if(WaveExists(timewave))
				if(waveexists(constraints))
					FuncFit/N=1/NTHR=0/Q/H=HoldStr FitSpeciesSum coef  slice/X=timewave /D/E=eps/C=Constraints
				else
					FuncFit/N/Q/H=HoldStr/W=2 FitSpeciesSum coef  slice/X=timewave /D/E=eps
				Endif
			Else
				if(WaveExists(Constraints))
					FuncFit/N=1/NTHR=0/Q/H=HoldStr FitSpeciesSum coef  slice /D/E=eps/C=Constraints
				Else
					FuncFit/N=1/NTHR=0/Q/H=HoldStr FitSpeciesSum coef  slice /D/E=eps
				Endif
			Endif
		Endif

		Return GetWavesDAtaFolder(SumTrans,2)
End

Function/T MakePositiveConstraints(Coef, SpeciesSelect) // make a constraints wave that requires all coefficients to be positive
//Coef - the linear coefficents for fitting the ODE solutions to the data
//SpeciesSelect - a 1D numeric wave that determins whether a given species will be included (1)
// Returns a 1D text wave with the nececary constrain expressions.

		WAVE Coef,SpeciesSelect
		Variable N = dimsize(Coef,0);
		Variable i = 0;
		DFREF MDFR = $ksMDFR
		Make/T/O/N=(N) MDFR:SumCoefConstraints
		WAVE/T SCC = MDFR:SumCoefConstraints
		Variable SCC_i =0

		if(!kPositiveCoefs)
			SCC = ""
			Return ""//GEtWavesdataFolder(SCC,2)
		Endif

		Do
			if(SpeciesSelect[i]>0.5)
				SCC[scc_i] = "K" +num2str(i) + ">1e-32"
				scc_i+=1;
			Else
				deletepoints scc_i, 1, SCC
```

```
			Endif
			//SCC[i+1] = "K" +num2str(i) + "< 1"
			i+=1//2
		While(i<N)//*2-1)
		Return GEtWavesDataFolder(SCC,2)
End

Function ZeroGuessIfHeld(HoldStr,Coef)// If a coeffcient is being held (per the hold string), set it to zero.
		String HoldStr // the hold string required by CurveFit
		WAVE Coef // the linear fit coefficients for fitting the ODE model to the data
		Variable N = dimsize(Coef,0)
		Variable i = 0;
		Do
			if(str2num(holdstr[i])>0)
				Coef[i] = 0
			Endif
			i+=1
		While(i<N)
End

Function/T MakeHoldStr(SpeciesSelect) // Make a hold string based on the species that are going to be included. If it's not included, hold it.
		Wave SpeciesSelect // The numeric wave specify whether a given ODE species will be included in the fit.
		// Returns a hold string (e.g "111001") that can be fed to CurveFit or FuncFit to hold specified coefficients
		Variable N = dimsize(SpeciesSelect,0)
		Variable i = 0
		String HoldStr = ""
		Do
			If(SpeciesSelect[i])
				HoldStr += "0"
			Else
				HoldStr += "1"
			Endif

			i+=1;
		While(i<N)

		Return HoldStr
End

Function FitImage(Image [Species]) // Do the fit on a whole image of data.
		WAVE Image, Species; // the input image data
		DFREF MDFR = $ksMDFR
		if(paramisdefault(Species))

			Wave Species=MDFR:Species; // the solution of the ODE model
		Endif
		// Returns ChiSq, the sum of all the variances
		// Creates an image of the fit, and of the residuals
		// creates a set of species associated spectra giving the coeffcients as a function of wavelength for each species.
		Variable NE = dimsize(Image, 0);
		Variable i = 0;

		Variable NSpecies = dimsize(Species, 1);
		Make/O/N=(NE, NSpecies) MDFR:Species_Associated_Spectra
		WAVE SAS = MDFR:Species_Associated_Spectra
		String xpath = GetNote(image, "WL")
```

```
	SetNote(SAS, xpath, "WL")
	WAVE Coef =  MDFR:SpeciesSumCoef;
	Duplicate/O Species MDFR:NormSpecies
	WAVE NormSpecies = MDFR:NormSpecies;


	Duplicate/O Image MDFR:ImageFit
	WAVE ImF = MDFR:ImageFit;
	Duplicate/O Image MDFR:ImageResid
	WAVE ImResid = MDFR:ImageResid
	WAVE TB = MDFR:Time_Base


	ConvolveEachCol(NormSpecies, TB) // this should only be used for EPR/TRMC data where an exponentially modified gaussian is required for the IRF.
	NormalizeEachCol(NormSpecies)

	WAVE Slice = $GetTimeSlice(Image, i);

	Duplicate/O Slice MDFR:SliceFit
	WAVE SliceFit = MDFR:SliceFit;

	Variable TimerVar = StartMSTimer
	xpath = GetNote(image, "t")
	WAVE Xwave = $xpath
	Duplicate/O Coef MDFR:SumFitEps
	WAVE Eps = MDFR:SumFitEps
	WAVE SpeciesSelect = MDFR:SpeciesSelect
	Eps = 1e-6
	WAVE WL = $GetNote(Image, "WL")
	Variable GSBBool = SpeciesSelect[kGSB_Species]
	Do
		WAVE Slice = $GetTimeSlice(Image, i);

		if(WL[i] > kMaxGSBWL && GSBBool)
			SpeciesSelect[kGSB_Species] = 0
		Endif

		WAVE ST = $FitSlice(slice,NormSpecies, Coef, Eps,SpeciesSelect)
		SAS[i][] = Coef[q]
		SliceFit[] = interp(xwave[p], TB, ST);
		ImF[i][] = SliceFit[q];
		//ImResid = SliceFit[q]-Slice[q];

		i+=1;
	While(i<NE)
	SpeciesSelect[kGSB_Species] = GSBBool
	imResid = ImF-Image
	print stopmstimer(TimerVar)

	Duplicate/O ImREsid MDFR:ImVar
	WAVE Var = MDFR:ImVar
	Var = ImResid^2
	Wavestats/Q Var
	Variable ChiSq = V_sum
	Return ChiSq
End
```

```
Function FitImageFromMenu()
        WAVE image = csrwaveref(A)
        If(WaveExists(Image) && dimsize(image,0) > 0 && dimsize(image,1) > 0)
                FitImage(Image)
                DFREF MDFR = $ksMDFR
                PlotMatByNote(MDFR:ImageFit)
                PlotMatByNote(MDFR:ImageResid)
                PlotMatByNote(MDFR:Species_Associated_Spectra)
        Endif

End

Function/C FitSlices(Slices, Species, SpeciesSelectSlices, weights) // A variant of FitImage, but modified to do some special things with manually
// selected time slices. Here, you can Specify which species contribute to each slice, allowing you to include information not otherwise
// available to the algorithm. For example, if you know where a certain species is spectrally, and it's cleanly seperated from the others
// you can make that explicit in the fit to good result. The is especially useful for ground-state bleach contributions.
        WAVE Slices, Species; // same as for ImageFit
        WAVE/T SpeciesSelectSlices; // a 1D text wave containing semiconoln seperated binary lists, indicating whether a given species
        Wave Weights
        //should be included in the fit for a corresponding time slice.
        // Returns ChiSq, the quality of the fit.
        Variable NE = dimsize(Slices, 0);
        Variable i = 0;
        DFREF MDFR = $ksMDFR
        Variable NSpecies = dimsize(Species, 1);
        Make/O/N=(NE, NSpecies) MDFR:Species_Associated_Spectra
        WAVE SAS = MDFR:Species_Associated_Spectra
        WAVE SL = MDFR:SpeciesLinkage

        //String xpath = GetNote(Slices, "WL")
        //SetNote(SAS, xpath, "WL")
        WAVE Coef =  MDFR:SpeciesSumCoef;
        Duplicate/O Species MDFR:NormSpecies
        WAVE NormSpecies = MDFR:NormSpecies;


        Duplicate/O Slices MDFR:SlicesFit
        WAVE SlicesFit = MDFR:SlicesFit;
        Duplicate/O Slices MDFR:SlicesResid
        WAVE SlicesResid = MDFR:SlicesResid
        WAVE TB = MDFR:Time_Base


        ConvolveEachCol(NormSpecies, TB) // this should only be used for EPR/TRMC data where an exponentially modified gaussian is required for the IRF.
        //NormalizeEachCol(NormSpecies)

        WAVE Slice = $GetTimeSlice(Slices, i);
        WAVE Weight = $GetTimeSlice(Weights,i);

        Duplicate/O Slice MDFR:SliceFit
        WAVE SliceFit = MDFR:SliceFit;

        Variable TimerVar = StartMSTimer
        String xpath = GetNote(Slices, "t")
        WAVE Xwave = $xpath
```

```
	String TN
	Duplicate/O Coef MDFR:SumFitEps
	WAVE Eps = MDFR:SumFitEps
	Eps = 1e-6
	//WAVE SpeciesSelect = MDFR:SpeciesSelect
	String SS_s = ksMDFR + ":SpeciesSelect"
	WAVE SS_w = $SS_s

	SumLinkedSpecies(NormSpecies, SS_w, SL) // sum up any trajectories that are identified as being spectrally linked, and don't fit to the higher number trajectories
	// were include din this sum.
	if(kNormalize >0)
		NormalizeEachCol(NormSpecies) // normalize the trajectories to either + or -1.
	Endif

	Do
		WAVE Slice = $GetTimeSlice(Slices, i);
		WAVE Weight = $GetWeightSlice(Weights,i);
		WAVE/T SpeciesSelect =$ODEs#List2Wave(SpeciesSelectSlices[i], SS_s)
		WAVE ST = $FitSlice(slice,NormSpecies, Coef, eps,SpeciesSelect, Weight=weight)
		SAS[i][] = Coef[q]
		SliceFit[] = interp(xwave[p], TB, ST);
		SlicesFit[i][] = SliceFit[q];
		//ImResid = SliceFit[q]-Slice[q];
		TN = "Fit_" + num2str(i);
		PlotifRows(SlicesFit, "STF_Graph", "t", i, TN);
		ModifyGraph lstyle($TN)=8
		i+=1;
	While(i<NE)

	SlicesResid = (Slices-SlicesFit)
	print stopmstimer(TimerVar)

	Duplicate/O SlicesResid MDFR:SlicesVar
	WAVE Var = MDFR:SlicesVar
	Variable w = 1//1.16e-4 // test weight value.
	Var = (SlicesResid/weights)^2
	Wavestats/Q Var
	Variable ChiSq = V_sum/V_npnts
	Return cmplx(V_sum,V_npnts)
End



Function SumLinkedSpecies(Species, SpeciesSelect, SpeciesLinkage) // this function operates on the species wave, the SpeciesSelect wave, and the SpeciesLinkage wave.
// use the SpeciesLinkage wave to link two or more species so that the same spectrum will be used to fit both of them.
// This is done by summing all the indicated species together into one trajectory,then setting SpeciesSelect to 0 for all but one of the involved trajectories
	WAVE Species, SpeciesSelect, SpeciesLinkage;
	Variable SpeciesIndex
	Variable LinkVar
	Variable NSpecies = dimsize(species,1)
	Variable i = 0;
	variable j = 1;
	Do
		do
			if(SpeciesLinkage[i] == SpeciesLinkage[j])
				Species[][i] += Species[p][j]
```

```
                        SpeciesSelect[j] = 0;
                Endif
                j+=1;
            while(j+1<NSpecies)
            i+=1; j=i+1;
        While(i+1<NSpecies)
End


Function SetLinkedSpeciesSelect(SpeciesSelect, SpeciesLinkage) // this function operates on the species wave, the SpeciesSelect wave, and the SpeciesLinkage wave.
// use the SpeciesLinkage wave to link two or more species so that the same spectrum will be used to fit both of them.
// This is done by summing all the indicated species together into one trajectory,then setting SpeciesSelect to 0 for all but one of the involved trajectories
        WAVE SpeciesSelect, SpeciesLinkage;
        Variable SpeciesIndex
        Variable LinkVar
        Variable NSpecies = dimsize(SpeciesSelect,0)
        Variable i = 0;
        variable j = 1;
        Do
            do
                if(SpeciesLinkage[i] == SpeciesLinkage[j])
                        SpeciesSelect[j] = 0;
                Endif
                j+=1;
            while(j+1<NSpecies)
            i+=1; j=i+1;
        While(i+1<NSpecies)
End

Function FiniteSecondDiff(Iny, Inx) // Use finite differences to calculate the second derivitive using three or more data points
        WAVE Iny, Inx;

        If(dimsize(InY,0) != Dimsize(Inx,0) || Dimsize(InY,0) < 3)
            Print "error in 'FiniteSecondDiff': InY and InX must be equal in length, and at least three points long"
        Endif

        DFREF MDFR = $ksMDFR
        Variable N = dimsize(Iny,0)-1
        Make/O/N=(N) MDFR:FirstD
        WAVE FD = MDFR:FirstD
        Make/O/N=(N-1) MDFR:SecondD
        WAVE SD = MDFR:SecondD;
        Variable i = 0;

        Do
            FD[i] = (iny[i+1]-Iny[i])/(inx[i+1]-Inx[i])
            i+=1;
        While(i<N)

        i = 0;

        Do
            SD[i] = (FD[i+1]-FD[i])/(inx[i+1]-Inx[i])
            i+=1;
        While(i<N-1)
```

```
	Wavestats/Q SD

	Return V_avg
End

Function CalcHessianDiag(ParamIndex)
	variable ParamIndex
	DFREF MDFR = $ksMDFR
	Variable FiniteDifference = 0.1 // fractional change to make to each parameter about its fitted value for calculation of the second derivitive with respect to cha
	WAVE Params = MDFR:ParameterVals
	Variable NPoints = 3
	Make/N=(NPoints)/O MDFR:ChiSqVals, MDFR:ParamVar;
	WAVE CSV = MDFR:ChiSqVals
	WAVE PV = MDFR:ParamVar;
	Variable i = 0;
	Variable j = 0;
	Variable NParams = dimsize(Params,0)
	Variable OptVal = Params[ParamIndex]
	Variable ValNow = 0

		Do

				ValNow = OptVal/FiniteDifference


			PV[i] = ValNow
			Params[ParamIndex] = ValNow
			CSV[i] = real(SolveODEAndFitSlices())
			i+=1;
		While(i<NPoints)
		Params[ParamIndex] = OptVal

	Variable secondDeriv = FiniteSecondDiff(CSV, PV)
	Return SecondDeriv//2*(OptVal*FiniteDifference)^2;
End


Function CalcODEDeriv(ParamIndex, FDVal)
	variable ParamIndex, FDVal
	DFREF MDFR = $ksMDFR
	Variable FiniteDifference = FDVal// fractional change to make to each parameter about its fitted value for calculation of the second derivitive with respect to ch
	WAVE Params = MDFR:ParameterVals
	Variable NPoints = 2
	Make/N=(NPoints)/O MDFR:ChiSqVals, MDFR:ParamVar;
	WAVE CSV = MDFR:ChiSqVals
	WAVE PV = MDFR:ParamVar;
	Variable i = 0;
	Variable j = 0;
	Variable NParams = dimsize(Params,0)
	Variable OptVal = Params[ParamIndex]
	Variable ValNow = 0;
	Variable/C ChiSq
		Do

			ValNow = OptVal+OptVal*FiniteDifference*i
```

```
                PV[i] = ValNow
                Params[ParamIndex] = ValNow
                ChiSq = SolveODEAndFitSlices()
                CSV[i] = real(ChiSq)//Imag(ChiSq)
                i+=1;
            While(i<NPoints)
            Params[ParamIndex] = OptVal

        Variable Deriv = (CSV[1]-CSV[0])/(PV[1]-PV[0])
        Return Deriv
End

Function CalcODEDeriv2(Pi1, Pi2, FDVal)
        Variable Pi1, Pi2, FDVal
        DFREF MDFR = $ksMDFR
        Variable FiniteDifference = FDVal // fractional change to make to each parameter about its fitted value for calculation of the second derivitive with respect to c
        WAVE Params = MDFR:ParameterVals
        Variable NPoints = 2
        Make/N=(NPoints)/O MDFR:ChiSqVals2, MDFR:ParamVar2;
        WAVE CSV = MDFR:ChiSqVals2
        WAVE PV = MDFR:ParamVar2;
        Variable i = 0;
        Variable j = 0;
        Variable NParams = dimsize(Params,0)
        Variable OptVal = Params[Pi2]
        Variable ValNow = 0;

            Do

                ValNow = OptVal+OptVal*FiniteDifference*i

                PV[i] = ValNow
                Params[Pi2] = ValNow
                CSV[i] = CalcODEDeriv(Pi1, FiniteDifference)
                i+=1;
            While(i<NPoints)
            Params[Pi2] = OptVal

        Variable Deriv = (CSV[1]-CSV[0])/(PV[1]-PV[0])
        Return Deriv
End

Function/T CalcODEErrorMatrix()
        DFREF MDFR = $ksMDFR
        WAVE Params = MDFR:ParameterVals
        WAVE ParamHolds = MDFR:ParameterHolds
        String Free_Param_Names = GetFreeParams(Params, ParamHolds)
        WAVE HessPerams = $StringFromList(0,Free_Param_Names,";")
        WAVE HessPeramIndexes = $StringFromList(1,Free_Param_Names,";")
        Variable NFree = Dimsize(HessPerams,0);
        Make/O/N=(NFree,NFree) MDFR:ODE_Hessian
        WAVE Hession = MDFR:ODE_Hessian
        Hession = 0;
        Variable FDVal = 0.08

        Variable i = 0;
```

```
	Variable j = 0;
	Variable Diff2

	Do
		Do
			Diff2 = CalcODEDeriv2(HessPeramIndexes[i], HessPeramIndexes[j], FDVal)
			Hession[i][j] = Diff2
			i+=1
		While(i<NFree)
		i=0;
		j+=1;
	While(j<NFree)

	Variable/C ChiSq = SolveODEAndFitSlices() // recalculate the best fit so that we can use the variance to calculate a good value of reduced-chisq

	WAVE SlicesVar = MDFR:SlicesVar // variance of the fits
	WAVE TimeBase = $GetNote(SlicesVar,"t") // experimental time base
	findlevel/P/Q TimeBase, 1e-13; // find out where the time crosses the lowest reasonable time point. e.g. 100 fs for TA, 100 ps for TRMC/trEPR
	Variable FirstGoodPoint = V_LevelX
	Variable Rows =dimsize(SlicesVar,0)
	Variable Cols = dimsize(SlicesVar,1)
	Duplicate/O/R=[0,Rows-1][FirstGoodPoint, Cols] SlicesVar MDFR:SlicesVar_Trunc // truncate the varaince wave to include only good time points were the fit is expec
	WAVE SVTrunc = MDFR:SlicesVar_Trunc
	Wavestats/Q SVTrunc;
	Variable rChiSq = V_sum/(V_npnts-NFree)//Real(ChiSq)/Imag(ChiSq) // calculate-reduced ChiSq from the variance wave.
	//print "rChiSq = " + num2str(rChiSq);
	Duplicate/O Hession MDFR:ODECovariance
	Hession/=2 // division of the Hession by 2 is conventional. I have not found an explaination for why.
	WAVE covar = MDFR:ODECovariance
	MatrixInverse/O Covar // calculate the covariance as the inverse matrix of the Hessian.
	Covar *= rChiSq		// Multiply the co-variance wave by reduced-ChiSq. This is vital to obtain properly scaled errors if weights are not used in the fit.
						// This is equivlent to using the square-root of the average variance between the data and the model as a constant
						// If the model fits the data well, it is equivelent to using the average standard deviation associated with measu
						//
	Duplicate/O Covar MDFR:ODEError
	Wave Error = MDFR:ODEError
	Error = sqrt(covar) // calculate the error matrixas the square root of the scaled covariance matrix. The diagonal elements are the parameter error estimates.

	Return GetWavesDAtaFolder(Error,2) + ";" + GetWavesDAtaFolder(HessPeramIndexes,2)
End

Function/T CalcODEParamErrors()
	String ErrorDataNames = CalcODEErrorMatrix()
	WAVE ErrorMatrix = $STringFromList(0,ErrorDataNames,";")
	WAVE ParamIndexes = $STringFromList(1,ErrorDataNames,";")
	DFREF MDFR = $ksMDFR;
	WAVE 	Params = MDFR:ParameterVals

	Duplicate/O Params MDFR:ParameterErrors
	WAVE Errors = MDFR:ParameterErrors;
	Errors = nan;
	Variable N = dimsize(ParamIndexes,0)
	Variable i = 0;

	Do
		Errors[ParamIndexes[i]] = ErrorMatrix[i][i]
```

```
		i+=1
	While(i<N)

	i=0;

	String TableName = "ActiveParameters"
	dowindow/F $TableName // check to see if the coefficients table exists and bring it to the front if it does.
if(V_flag) // only create a new one if it does not.
	String TableInfoSTr = tableInfo(TableName,-2)
	String ColumnsStr = StringbyKey("COLUMNS", TableInfoStr, ";", ":")
	Variable Columns = str2num(ColumnsStr)
	String ColPath = ""//StringbyKey("WAVE" TableInfoStr, ";", ":")
	Do
		TableInfoStr= tableInfo(TableName,i)
		ColPath = StringbyKey("WAVE", TableInfoStr, ";", ":")
		if(StringMatch(ColPath, GetWavesDataFolder(Errors,2)))
			Return GetWavesDAtaFolder(Errors,2)
		Endif
		i+=1;
	While(i<Columns)

	AppendToTable/W=$TableName Errors

	Else
	Edit/N=$TableName Params, Errors

Endif

	Return GetWavesDAtaFolder(Errors,2)
End

Function/T GetFreeParams(Params, ParamHolds)
	WAVE Params, ParamHolds
	Variable N = dimsize(Params,0)
	Variable i = 0;
	Variable Count = 0;
	DFREF MDFR = $ksMDFR
	Make/O/N=1 MDFR:HessPerams, MDFR:HessPeramIndex
	WAVE HP = MDFR:HessPerams
	WAVE HPI = MDFR:HessPeramIndex
	STring Dimlabel

	Do
		if(PAramHolds[i] == 0)
			Redimension/N=(count+1) HP, HPI;
			HP[count] = Params[i]
			dimlabel = Getdimlabel(Params, 0, i)
			Setdimlabel 0, count, $dimlabel, HP
			HPI[count] = i
			COunt+=1;
		Endif
		i+=1
	While(i<N)

	Return GetWavesDataFolder(HP,2) + ";" + GetWavesDataFolder(HPI,2)
End
```

```
Function NormalizeEachCol(Matrix) // Normalizes each column of the input matrix (2D wave) preserving their sign.
	WAVE Matrix
	DFREF MDFR = $ksMDFR;
	Duplicate/O/R=[][0] Matrix MDFR:Temp
	WAVE Temp = MDFR:Temp;

	Variable i = 0;
	Variable N = dimsize(Matrix,1)

	Do
		Temp[] = Matrix[p][i]
		Wavestats/Q Temp;

		if(V_sum>0) // normalize, but don't change the sign. Negative trajectories normalize to -1
			Temp/=V_max
		Else
			Temp/=abs(V_min)
		Endif

		Matrix[][i] = Temp[p];
		i+=1;
	While(i<N)

	killwaves/z temp

End

Function ConvolveEachCol(Species, Time_Base) // numerically convolves each column with an expontential function, using the cavity time constant defined in ODEs.
	WAVE Species, Time_Base
	if(!kApplyCavResp)
		Return 0
	Endif
	Variable CavTime = kCavTime
	DFREF MDFR = $ksMDFR;
	Duplicate/O/R=[][0] Species MDFR:Temp
	WAVE Temp = MDFR:Temp;

	Variable i = 0;
	Variable N = dimsize(Species,1)
	Variable NPnts = dimsize(Species,0)

	Do
		Temp[] = Species[p][i]
		Duplicate/O temp MDFR:TempExp
		WAVE TempExp = MDFR:TempExp
		TempExp = exp(-Time_Base/CavTime)
		Wavestats/Q TempExp
		TempExp/= V_sum
		Convolve TempExp, Temp;
		Redimension/N=(NPnts) Temp

		Species[][i] = Temp[p];
		i+=1;
	While(i<N)
```

```
	killwaves/z temp
	killwaves/z tempExp
End

Function/T LoadImagePath(path, keyword, extStr)			//This function will load all the text files from a specified folder who's names contain the keywor
											//The file will be loaded as a single matrix
											//Path is an igor path
											// ExtStr is a string giving the text file extensi

	String path, keyword, ExtStr;
	String filename, OutputName, OutPath;
	String OutList = ""
	Variable index, index2, length, ColumnIndex;

	index = 0;
	index2=0;


	do
		filename = IndexedFile($path, index, ExtSTr);			//Set the value of the string variable "filename" to the name of the ith text

		if(strlen(filename) == 0)						//Check to see that the the file name is real (non zero), so that the fun
			//if(index == 0
			Print "********Search and Load Complete*********"
			Print "Files Searched:"
			Print index
			Print " "
			Print "Files loaded:"
			Print index2
			Print "************************************************"

			break;
		endif

		OutputName = RemoveFileExt(Filename)
		OutPutName = cleanupname(OutPutName,0)
		//OutPutName = ShortenNameBy(OutPutName,3)
		OutPath = "root:" + OutPutName


		if (strsearch(filename, keyword, 0) != -1)			//Search "filename" for the keyword, and continue if it is found anywhere in the string.
				//String S_WaveNames
				OR_MakeFolderTree(OutPath)
				LoadWave/K=1/Q/M/O/N=$OutputName/J/L={0, 0, 0, 0, 0}/P=$path filename; //Load the file as a matrix with all columns
				OutPutName = OutPAth +":" + OutPutNAme
				WAVE NowWave = $stringfromlist(0,S_wavenames, ";")
				Duplicate/O NowWave $OutPutName
				KillList(S_WaveNames, ";")
				OutList += OutPutName + ";"
			index2 = index2 + 1;					//Incriment the loaded files counter
		endif

		ColumnIndex = 0;
		index = index + 1;						//Incriment the searched files counter

	While(1)
```

```
        Return OutList;

End

//*******************************************************************************************************************************************************************************
Function/T ShortenNameBy(Str,N) // take an input string and reduce it's length to N less than the legally allowed limit by cutting characters from the end.
        String Str
        Variable N
        Variable LegalLen = 30
        Variable Len = strlen(Str)
        Variable AllowedLen = 30-N

        Do
                Str = removeending(Str)
        While(StrLen(str)>AllowedLen)

        Return Str
ENd



Function LoadImageByKey(keyword, type) // give it a keyword that is in the filenames you want to load, then point it at the right folder when prompted
// It will load the data as a single matrix, and assuming that the first column is time and the first row is wavelength, create those
// 1D waves. It will store references to those waves in key=value format in the wavenotes for later retreival.

        String keyword;
        Variable type // type of image. 1 == TA, 2 == TRPL
        //Variable columns;

        Newpath path;
        pathinfo path;
        if(!V_flag)
                REturn 1
        Endif

        String LoadedImages = LoadImagePath("path", keyword, ksFileExt);

        if(itemsinlist(LoadedImages,";") > 0)
                SplitXYList(LoadedImages,Type)
        Endif

End

Function LoadImageByKeyPrompt()
        String keyword
        variable type
        prompt keyword, "keyword";
        prompt type, "type of image", popup, "TA;TRPL"
        doprompt/HELP="All files in the target folder with the right file extension (csv) will be loaded if they have the keyword (case sensitive) in their name" "Enter a
        if(V_flag)
                return 1
        Else

                        LoadImageByKey(keyword, type)

        Endif
```

```
End

Function/T RemoveFileExt(FilenameStr) // takes a string, removes any pesky file extension that may be there.
	String FilenameSTr
	Variable N = itemsinlist(FileNameStr, ".")
	String OutStr = removelistitem(N-1, FileNameStr, ".")
	OUtStr = removeEnding(OUtSTr)
	Return OutStr
End

Function KillList(Inlist, sepstr) // kill all waves found in the input list of wave reference strings
	STring InList, sepstr
	Variable N = itemsinlist(Inlist, Sepstr)
	Variable i = 0;

	Do
		WAVE NowWave = $StringFromList(i, inlist, sepstr)
		KillWaves/Z NowWave
		i+=1
	While(i<N)

End

Static Function OR_MakeFolderTree(DFTreeList) // Given a string containing an Igor folder path, this function will create it if it does not exist
// and do nothing if it does.

	String DFTreeList;
	String DFName; // Declare a string variable that will contain the name of each sucessive wave on which operations can be performed.
	Variable i, N;
	DFREF  CDFR = GetDataFolderDFR();
	i = 0;
	DFName = StringFromlist(0,DFTreeList, ":")

	if(stringmatch(DFName, "root"))
		setdatafolder root:
	endif

	DFTreeList = removefromlist("root", DFTreeList, ":")

	N = ItemsInList(DFTreeList, ":")

	Do
		DFName = StringFromlist(i,DFTreeList, ":")
		NewDataFolder/O/S $DFName
		i += 1;
	While(i<N)

	SetDataFolder CDFR;
End

Function SplitXY(InMAt,type) // given an input matrix, this function attempts to split out seperate time and wavelength waves for later use
	WAVE InMat // take an input matrix who's first row is time and who's first column is wavelength. Split these out into seperate 1D waves, and include wavenotes giv
	Variable type
	STring SourcePath = GetWavesDAtaFolder(InMat,2)

	STring Out_t = SourcePath +"_t"
```

```
	String OUt_WL = SourcePath+"_WL"

	if(type == 1)
		Duplicate/O/R=[0][] InMat $Out_t
		Duplicate/O/R=[][0] InMAt $Out_WL
	Else
		Duplicate/O/R=[][0] InMat $Out_t
		Duplicate/O/R=[0][] InMAt $Out_WL
	Endif

	WAVE Ot = $Out_t
	WAVE OWL = $Out_Wl

	if(type ==1)
		MatrixTranspose Ot
	Else
		MatrixTranspose OWL
		MatrixTranspose InMat
	Endif

	Variable Nt = dimsize(Ot,0)
	Variable NWL = dimsize(OWL,0)
	Redimension/N=(Nt) Ot
	Redimension/N=(NWL) OWL
	OWL[0] = OWL[1] - (OWL[2]-OWL[1])
	//Deletepoints 0, 1, Ot, OWL
	Deletepoints/m=0 0, 1, InMat
	Deletepoints/M=1 0, 1, InMat
	Ot*=kTimeUnit // convert units, for TA this is often 10^-12
	FillFirstPoint(Ot)

	String NoteString = "WL=" + Out_WL +",t=" + Out_t + ",type=" + num2str(type)
	Note/K/NOCR InMat, NoteString;

End

Function SplitXYList(ImageList,type) // Call SplitXY for a list of input images
	String ImageList // string containing a semicolon seperated list of wave references
	Variable type
	Variable N = itemsinlist(ImageList);
	Variable i =0;

	Do
		WAVE NowIm = $StringfromList(i, ImageList)
		SplitXY(NowIm,type)
		i+=1
	While(i<N)
End

static Function/T GetNote(Matrix, keystring) // retrieve the value of a wavenote in Matrix based on the keystring
	 WAVE MAtrix;
	 String keystring
	 string wavenote = note(matrix);
	 String desired = stringbykey(keystring, wavenote, "=", ",")
	 REturn desired
 End
```

```
 Function SetNote(Inwave, NoteStr, KeyString) // write a new wavenote, overwriting any that may already be present.
        WAVE InWAve // wave we are adding the note to
        String NoteStr // string containing the note content
        String KeySTring // string giving the keyword by which said note can be retrieved.
        String NoteString = KeyString+"="+NoteStr;
       Note/K/NOCR Inwave, NoteString

End

 Function SetNoteByKey(Inwave, NoteStr, KeyString) // write a new wavenote, overwriting any that may already be present.
        WAVE InWAve // wave we are adding the note to
        String NoteStr // string containing the note content
        String KeySTring // string giving the keyword by which said note can be retrieved.

        string wavenote = note(Inwave);
        String desired = stringbykey(keystring, wavenote, "=", ",")
        String ModWaveNote = ""
        if(strlen(desired)>0)
                ModWaveNote = ReplaceStringByKey(KeyString, WaveNote, NoteStr, "=", ",")
        Else
                Modwavenote = WaveNote+ "," + KeySTring +"=" + NoteStr;
        Endif

       Note/K/NOCR Inwave, ModWaveNote

End

 Function PlotMatByNote(Matrix) // attempt to plot an image from the given matrix, using the wavenotes to retrieve the X and Y waves with which to scale the axes
        WAVE Matrix
        String WL  = GetNote(Matrix, "WL")
        string t = GetNote(Matrix, "t")
 //        variable type = str2num(getNote(Matrix, "type"))

        if(!Waveexists($WL) && !WaveExists($t))
                Print "Either the time or wavelength waves were missing... abort!"
                Return -1
        Endif

        if(waveexists($WL) && WaveExists($t)) // if there is both x and y waves plot it as an image.
        WAVE WLW = $WL
        WAVE tW = $t
        Variable NWL = dimsize(WLW,0)
        Variable Nt = dimsize(tw,0)
        Variable NRow = Dimsize(Matrix,0)
        Variable NCol = dimsize(Matrix,1)
        if(NWL-1 == NRow && Nt-1 == Ncol)// && type == 1)
                Display;AppendImage Matrix vs {$WL,$t}
        //Elseif(NWL-1 == NCol && Nt-1 == NRow)
        //        Display;AppendImage Matrix vs {$t,$WL}
        Else
                Print "either or both x-waves are the wrong length for the matrix!"
                return -1
        Endif
                ModifyImage $nameofwave(Matrix) ctab= {*,*,Geo,0}
```

```
                ModifyGraph log(left)=1
                ModifyGraph width=360,height=360
        Elseif(WaveEXists($WL)) // if there are only x-waves plot it as multiple traces.
                WAVE wavelength = $WL
                Variable N = dimsize(Matrix,1)
                Variable i = 0

                Do
                        PlotifCols(Matrix, "Species_Associated_Spectra", "WL", i, "species_"+num2str(i))
                         i+=1;
                While(i<N)
        Endif


End

Function PlotSelectedMats() // Look at what is selected in the data browser and try to plot them all.

        Variable i = 0;
        Do
                String Path = GetBrowserSelection (i)

                if(WaveExists($Path))
                        if(dimsize($path,1) >1)
                                PlotMatByNote($path)
                        Else
                                 Plot1DByNote($path)
                        Endif

                Else
                        Break
                Endif
                i+=1
        While(1)

End

Function Plot1DByNote(Inwave) // same as PlotMatByNote, but for 1D waves that may have x-values referenced in their notes.
        WAVE Inwave
        Variable type = str2num(GetNote(inwave,"type"))

        Variable PlotSuccess = Plotif(Inwave, "Time_Slice", "t")
                if(type == 1)
                        dowindow/F Time_Slice
                        ModifyGraph log(bottom)=1
                Endif
                ModifyGraph tick=2,mirror=1,fStyle=1,fSize=12;DelayUpdate
                Label left "\\F'Symbol'D\\F]0OD";DelayUpdate
                Label bottom "time (s)"
                ModifyGraph zero(left)=1


        If(!PlotSuccess)
                PlotSuccess = Plotif(inwave, "WL_Slice", "WL")
                ModifyGraph tick=2,mirror=1,fStyle=1,fSize=12;DelayUpdate
                Label left "\\F'Symbol'D\\F]0OD";DelayUpdate
```

```
		Label bottom "wavelength (nm)"
		ModifyGraph zero(left)=1

	Endif

	if(!PlotSuccess)
		print ""
		Plotif(InWave, "Unknown_Slice", "")
		ModifyGraph tick=2,mirror=1,fStyle=1,fSize=12;DelayUpdate
		Label left "unknown";DelayUpdate
		Label bottom "unknown"
	Endif
End

Function Plotif(Inwave, WindowName, key) // Plot the input wave if it does not alrady exist in teh named window, using key to specify a wave note that may contain an
// x-wave reference.
		Wave Inwave
		String WindowName, key;
		//Variable dim, index;
		//String IndexStr = ConstructWaveIndex(Inwave, Dim, index);
		//String IndexedWaveRef = GetWavesDataFolder(Inwave,2)
	//	  if(!wavedims(inwave)<2)
		//	    IndexedWaveRef = GetWavesDataFolder(Inwave,2)+IndexStr;
		//Endif


		WAVE xwave = $GetNote(InWave, key)

		if(!Waveexists(XWave) && strlen(key)>0)
			Return 0
		Endif

		Dowindow/F $WindowName

		if(!V_flag)
			Display/N=$WindowName
		Endif

		CheckDisplayed/W=$WindowName inwave // need to change this so that it checks the x-wave and replots as needed.
		String TN = CheckDisplayedWave(WindowName,GetWavesDataFolder(Inwave,2))

		WAVE XWaveNow = XWaveRefFromTrace(WindowName, TN)

		if(WaveExists(XWaveNow) && WaveExists(XWave) && !stringmatch(GetWavesDataFolder(XWaveNow,2), GetWavesDataFolder(Xwave,2))) // if the x-waves differ get ri
			//print tracenamelist(WindowName, ",", 1)
			RemoveFromGraph/W=$WindowName $TN
			V_flag = 0;
		Endif

		if(!V_flag)

			if(strlen(key) <1)
				Appendtograph/W=$WindowName Inwave
			Else
				Appendtograph/W=$WindowName InWave vs xwave
			Endif
```

```
		Endif

		Return 1
End

Function PlotifRows(Inwave, WindowName, key, index, TN) // same as plotif, but for 2D waves where we want to plot a specific row and give it a unique tracename
		Wave Inwave // input 2D wave
		String WindowName // Name of the window where the plot should be
		String key; // keyword that should specify an x-wave in the inwave wavenotes
		String TN; // desired name of the new trace in the graph
		Variable index;

		WAVE xwave = $GetNote(InWave, key)

		if(!Waveexists(XWave) && strlen(key)>0)
			Return 0
		Endif

		Dowindow/F $WindowName

		if(!V_flag)
			Display/N=$WindowName
			Legend/C/N=text0/A=MC
		Endif

		//CheckDisplayed/W=$WindowName inwave
		Variable TraceisOnGraph = CheckDisplayedTrace(WindowName, TN)
		WAVE XWaveNow = XWaveRefFromTrace(WindowName, TN)

		if(!stringmatch(NAmeofWave(XWaveNow), NameofWave(Xwave))&&TraceIsOnGraph) // if the x-waves differ get rid of the trace
			RemoveFromGraph/W=$WindowName $TN
			TraceIsOnGraph = 0;
		Endif

		if(!TraceIsOnGraph)
			String color = GetColorFromName(TN)//65535/index
			Variable R = Str2num(stringfromlist(0,color))
			Variable G = Str2num(stringfromlist(1,color))
			Variable B = Str2num(stringfromlist(2,color))
			if(R+G+B == 0)
				color = GetColorFromIndex(index)
				R = Str2num(stringfromlist(0,color))
				G = Str2num(stringfromlist(1,color))
				B = Str2num(stringfromlist(2,color))
			Endif

			if(strlen(key) <1)
				Appendtograph/C=(R,G,B)/W=$WindowName Inwave[index][]/TN=$TN
			Else
				Appendtograph/C=(R,G,B)/W=$WindowName InWave[index][]/TN=$TN  vs xwave
			Endif
			ModifyGraph lsize($TN)=2

		Endif
```

```
		Return 1
End

Function/T GetColor(WL) // given a wavelength, this function will return a list of RGB values to use.
	Variable WL
	String Color = ""
	Variable RCent = 650
	Variable GCent = 532
	Variable BCent = 450
	Variable width = 50
	Variable height = (1/(sqrt(2*pi)*width))*exp(-(((0))/width)^2/2)

	Variable R = (1/(sqrt(2*pi)*width))*exp(-(((WL-RCent))/width)^2/2)/height*65535
	Variable G = (1/(sqrt(2*pi)*width))*exp(-(((WL-GCent))/width)^2/2)/height*65535
	Variable B = (1/(sqrt(2*pi)*width))*exp(-(((WL-BCent))/width)^2/2)/height*65535



	color = num2str(R)+";"+num2str(G)+";"+num2str(B)+";"
	REturn Color
End

Function/T GetColorFromIndex(index)
	Variable Index
	Variable WL = 350 + index*20
	Return GetColor(WL)
End

Function/T GetColorFromName(Name)
	string name
	String WLStr = stringfromlist(1,name,"_");
	Variable WL = str2num(WLStr)
	STring Color
	if(WL >0)
		Color = GetColor(WL)
	Else
		Color = "0;0;0;"
	Endif

	Return Color
End


Function PlotifCols(Inwave, WindowName, key, index, TN) // same as plotif, but for 2D waves where we want to plot a specific column and give it a unique tracename
		Wave Inwave // input 2D wave
		String WindowName // Name of the window where the plot should be
		String key; // keyword that should specify an x-wave in the inwave wavenotes
		String TN; // desired name of the new trace in the graph
		Variable index;

		WAVE xwave = $GetNote(InWave, key)

		if(!Waveexists(XWave) && strlen(key)>0)
			Return 0
		Endif
```

```
		Dowindow/F $WindowName

		if(!V_flag)
			Display/N=$WindowName
			Legend/C/N=text0/A=MC
		Endif

		//CheckDisplayed/W=$WindowName inwave
		Variable TraceisOnGraph = CheckDisplayedTrace(WindowName, TN)
		WAVE XWaveNow = XWaveRefFromTrace(WindowName, TN)

		if(!stringmatch(NAmeofWave(XWaveNow), NameofWave(Xwave))&&TraceIsOnGraph) // if the x-waves differ get rid of the trace
			RemoveFromGraph/W=$WindowName $TN
			TraceIsOnGraph = 0;
		Endif

		if(!TraceIsOnGraph)
			variable color = 65535/index
			if(strlen(key) <1)
				Appendtograph/C=(color,65535-color,65535-color/2)/W=$WindowName Inwave[][index]/TN=$TN
			Else
				Appendtograph/C=(color,65535-color,65535-color/2)/W=$WindowName InWave[][index]/TN=$TN  vs xwave
			Endif
			ModifyGraph lsize($TN)=2

		Endif

		Return 1
End

Function CheckDisplayedTrace(WN, TN) // check if tracename (TN) apears in the named window (WN). If it does, return 1. If not, return 0;
	String WN, TN;
	STring TL = TraceNameList(WN, ";", 1)
	Variable N = itemsinlist(TL,";")
	Variable i =0;
	String TNN

	Do
		TNN = Stringfromlist(i,TL, ";")
		If(stringmatch(TN, TNN))
			REturn 1
		Endif
		i+=1;
	While(i<N)
	Return 0;
End

Function/T CheckDisplayedWave(WN,WR) // check if wavere reference (WR) apears in the named window (WN). If it does, return the tracename. If not, return "";
	String WN, WR;
	STring TL = TraceNameList(WN, ";", 1)
	Variable N = itemsinlist(TL,";")
	Variable i =0;
	String TNN

	Do
		TNN = Stringfromlist(i,TL, ";")
```

```
			WAVE WaveNow = TraceNameToWaveRef(WN, TNN)
			If(WaveExists(WaveNow) && stringmatch(WR, GetWavesDataFolder(WaveNow,2)))
				REturn TNN
			Endif
			i+=1;
		While(i<N)
		Return "";
End

Function/T GetAvgTimeSliceByCsr(Matrix)
		WAVE Matrix
		Variable t = qcsr(A)
		Variable WL = pcsr(A)

		Variable Navgs_t = 1
		Variable Navgs_WL = 1

		if(strlen(csrinfo(B))>0)
			Navgs_t = abs(pcsr(A)-pcsr(b))
			Navgs_WL = abs(qcsr(A)-qcsr(B))
		Endif

		if(Navgs_t<1 || stringmatch(num2str(Navgs_t), "NAN"))
			Navgs_t = 1
		Endif

		if(Navgs_WL<1|| stringmatch(num2str(Navgs_WL), "NAN"))
			Navgs_WL = 1
		Endif


			WAVE TS = $GetAvgSlice(Matrix, WL, Navgs_WL, 0)

			Return Getwavesdatafolder(TS,2)
End


Function PlotSlicesByACursor() // plot both time and wavelength slices, using the A cursor in the top graph as the target
		WAVE Matrix = csrwaveref(A)
		String WindowName = WinName(0, 1)

		if(!WaveExists(MAtrix) || dimsize(Matrix,0) <2 || Dimsize(Matrix,1) < 2)
			Print "An image needs to be in the top graph with the A-cursor on it somewhere!"
			Return -1
		Endif
		String type_s = getnote(Matrix,"type");
		Variable Type = str2num(type_s)
		Variable WL = qcsr(A)
		Variable t = pcsr(A)

		Variable Navgs_t = 1
		Variable Navgs_WL = 1

		if(strlen(csrinfo(B))>0)
			Navgs_t = abs(pcsr(A)-pcsr(b))
			Navgs_WL = abs(qcsr(A)-qcsr(B))
```

```
			if(pcsr(A) > pcsr(B)) // make sure that the lower of the two indicies is the first for purposes of runnning through averages.
				t = pcsr(B)
			endif

			if(qcsr(A) > qcsr(B))
				WL = qcsr(B)
			endif

		Endif

		if(Navgs_t<1 || stringmatch(num2str(Navgs_t), "NAN"))
			Navgs_t = 1
		Endif

		if(Navgs_WL<1|| stringmatch(num2str(Navgs_WL), "NAN"))
			Navgs_WL = 1
		Endif



		if(type == 1)
			WAVE TS = $GetAvgSlice(Matrix, WL, Navgs_WL, 1)//GetAvgTimeSlice(Matrix, t,3)
			Plot1DByNote(TS)
			WAVE WLS = $GetAvgSlice(Matrix, t, Navgs_t, 0)//GetAvgWLSlice(Matrix, WL, 3)
			Plot1DByNote(WLS)
		Else
			WAVE TS = $GetAvgSlice(Matrix, t, Navgs_t, 0)//GetAvgWLSlice(Matrix, t,3)
			Plot1DByNote(TS)
			WAVE WLS = $GetAvgSlice(Matrix, WL, Navgs_WL, 1)//GetAvgTimeSlice(Matrix, WL, 3)
			Plot1DByNote(WLS)
		Endif
		DoWindow/F $WindowName
End

Function AddSliceToFit() // use the A cursor in the top graph to extract a time slice that is added to the list of things to fit.
		WAVE Matrix = csrwaveref(A)
		//Variable t = qcsr(A)
		if(!WaveExists(MAtrix) || dimsize(Matrix,0) <2 || Dimsize(Matrix,1) < 2)
			Print "An image needs to be in the top graph with the A-cursor on it somewhere!"
			Return -1
		Endif

		Variable WL = pcsr(A)
		String ImageWinName = WinName(0,1)
		WAVE WLWave = $GetNote(MAtrix,"WL")
		Variable WL_Val = round(WLWave[WL])
		WAVE TS = $GetAvgTimeSliceByCsr(Matrix)//GetTimeSlice(Matrix, WL)
		//Plot1DByNote(TS)
		STring tnote = getnote(Matrix,"t")
		DFREF MDFR = $ksMDFR
		Variable N = dimsize(Ts,0)
		WAVE STF = MDFR:SlicesToFit
		WAVE/T SSS = MDFR:SpeciesSelectSlices
		WAVE SS = MDFR:SpeciesSelect
		WAVE SL = MDFR:SpeciesLinkage
		WAVE WW = MDFR:SlicesToFit_Weights
```

```
	SetLinkedSpeciesSelect(SS, SL)
	If(!Waveexists(STF)|| !WaveExists(SSS) ||!WaveExists(WW))
		Duplicate/O/R=[0][] Matrix MDFR:SlicesToFit
		WAVE STF = MDFR:SlicesToFit
		STF[0][] = TS[q]
		Make/T/O/N=(1) MDFR:SpeciesSelectSlices
		WAVE/T SSS = MDFR:SpeciesSelectSlices
		SSS[0] = ODEs#Wave2List(SS)
		Duplicate/O STF MDFR:SlicesToFit_Weights
		WAVE WW = MDFR:SlicesToFit_Weights;
		WW = 1//sqrt(STF);

	Else
		Variable M = dimsize(STF,0)
		Redimension/N=(M+1,N) STF
		redimension/N=(M+1,N) WW
		STF[m][] = TS[q]
		WW[m][] = 1//sqrt(TS[q]^2)
		Redimension/N=(M+1) SSS
		SSS[m] = ODEs#Wave2List(SS)
	Endif

	M = Dimsize(STF,0)
	PlotifRows(STF, "STF_Graph", "t", M-1, "Slice_"+num2str(WL_Val))
	ModifyGraph log(bottom)=1
	ModifyGraph tick=2,mirror=1,fStyle=1,fSize=12;DelayUpdate
	Label left "\\F'Symbol'D\\F]0OD";DelayUpdate
	Label bottom "time (s)"
	ModifyGraph tickUnit=1

	Dowindow/F Species_Selection
	if(!V_flag)
		Edit/N=Species_Selection SSS
	Endif

	SetNote(STF, tnote,"t")
	SetNote(WW, tnote,"t")
	DoWindow/F $ImageWinName
End

Function AddTransToFit() // use the A cursor in the top graph to extract a time slice that is added to the list of things to fit. But this is for 1D waves like TRPL or TR
	WAVE TS = csrwaveref(A)
	WAVE twave = csrXWaveRef(A)

	if(!WaveExists(TS) || dimsize(TS,0) <2 || Dimsize(TS,1) >1)
		Print "A 1-D plot needs to be the top graph with the A-cursor on it somewhere to select the data!"
		Return -1
	Endif

	if(!WaveExists(twave))
		String TName = getwavesdatafolder(TS,2) + "_t"
		Duplicate/O TS $TName
		WAVE twave = $Tname
		twave = x
	Endif
	//Variable t = qcsr(A)
```

```
	//Variable Index = csrInfo((A)
	String TransWinName = WinName(0,1)
	//WAVE WLWave = $GetNote(MAtrix,"WL")
//		Variable WL_Val = round(WLWave[WL])
	//WAVE TS = $GetAvgTimeSliceByCsr(Matrix)//GetTimeSlice(Matrix, WL)
	//Plot1DByNote(TS)
	STring tnote = GetWavesDAtaFolder(twave,2);
	String TraceName = stringbykey("TNAME",csrinfo(A), ":")
	DFREF MDFR = $ksMDFR
	Variable N = dimsize(Ts,0)
	WAVE STF = MDFR:SlicesToFit
	WAVE/T SSS = MDFR:SpeciesSelectSlices
	WAVE SS = MDFR:SpeciesSelect
	WAVE SL = MDFR:SpeciesLinkage
	WAVE WW = MDFR:SlicesToFit_Weights
	SetLinkedSpeciesSelect(SS, SL)
	If(!Waveexists(STF)|| !WaveExists(SSS) ||!WaveExists(WW))
		Duplicate/O TS MDFR:SlicesToFit
		WAVE STF = MDFR:SlicesToFit
		MatrixTranspose STF
		STF[0][] = TS[q]
		Make/T/O/N=(1) MDFR:SpeciesSelectSlices
		WAVE/T SSS = MDFR:SpeciesSelectSlices
		SSS[0] = ODEs#Wave2List(SS)
		Duplicate/O STF MDFR:SlicesToFit_Weights
		WAVE WW = MDFR:SlicesToFit_Weights;
		WW = 1//sqrt(STF);
	Else
		Variable M = dimsize(STF,0)
		Redimension/N=(M+1,N) STF
		redimension/N=(M+1,N) WW
		STF[m][] = TS[q]
		WW[m][] = 1//sqrt(TS[q]^2)
		Redimension/N=(M+1) SSS
		SSS[m] = ODEs#Wave2List(SS)
	Endif

	SetNote(STF, tnote,"t")
	SetNote(WW, tnote,"t")

	M = Dimsize(STF,0)
	PlotifRows(STF, "STF_Graph", "t", M-1, tracename)
	ModifyGraph log(bottom)=1
	ModifyGraph tick=2,mirror=1,fStyle=1,fSize=12;DelayUpdate
	Label left "\\F'Symbol'D\\F]0OD";DelayUpdate
	Label bottom "time (s)"

	Dowindow/F Species_Selection
	if(!V_flag)
		Edit/N=Species_Selection SSS
	Endif

	SetNote(STF, tnote,"t")
	SetNote(WW, tnote,"t")
```

```
	DoWindow/F $TransWinName
End

Function ClearSlicesToFit() // clear the list of slices to fit. Use this when you want to start over.
	DFREF MDFR = $ksMDFR;
	WAVE STF = MDFR:SlicesToFit
	WAVE Fits = MDFR:SlicesFit
	WAVE SS = MDFR:SpeciesSelect
	Dowindow/K STF_Graph
	Killwaves/Z STF, Fits;

	Dowindow/K Species_Selection
	Killwaves/Z SSS
End

Function/C SolveODEAndFitSlices() // solve the ODEs and the linear combination of the solutions to the set of chosen slices.

	DFREF MDFR=$ksMDFR
	Wave pw = MDFR:ParameterVals		// The rate constants and other wave-defining coefficients go here
	Wave xw= MDFR:Time_Base// This is the time base for the transients, but might not be used.
	DFREF MDFR =$ksMDFR
	Wave species = MDFR:Species
	Wave Transfer = MDFR:Transfer
	Wave Time_Base = MDFR:Time_Base
	Variable NEqn = itemsinlist(ksEqnNames)

	Species = 0;
	SolveODE(Time_base, "eqn", pw, species)
	WAVE STF = MDFR:SlicesToFit
	WAVE SpeciesSelectSlices = MDFR:SpeciesSelectSlices
	WAVE Weights = MDFR:SlicesToFit_Weights
	Variable/C ChaiSq = FitSlices(STF, Species,SpeciesSelectSlices, weights)
	Return ChaiSq
End

Function MeritSlices(w, xw) : FitFunc // Like SolveODEAndFitSlices, but this is the specific function format required by the Optimize function, which will actually fit th
// ODE parameters to the data.

	Wave w// This is a wave containing the parameter index of the corresponding xw value
	WAVE XW // these are the subset of parameters we want to fit
	DFREF MDFR=$ksMDFR
	Wave Params = MDFR:ParameterVals		// The rate constants and other wave-defining coefficients go here
	Wave species = MDFR:Species
	Wave Transfer = MDFR:Transfer
	Wave Time_Base = MDFR:Time_Base
	Variable NEqn = itemsinlist(ksEqnNames)

	Species = 0;
	ReadXW(Xw, w, Params)
	SolveODE(Time_base, "eqn", Params, species)
	WAVE STF = MDFR:SlicesToFit
	WAVE SpeciesSelectSlices = MDFR:SpeciesSelectSlices
	WAVE Weights = MDFR:SlicesToFit_Weights
	Variable/C ChiSq = FitSlices(STF, Species,SpeciesSelectSlices, weights)
	Doupdate
	Return Real(ChiSq)/Imag(ChiSq)
```

```
End


Function/T SetXW(Params, HoldWave, XSA) // take the ODE parameters and a holdwave with 1 or 0 to indicate whether a parameter should be held (1). Construct the x vector
// of values to be fit by the optimize function. Limiting the input XW list seems to be the only way to hold parameters.
	WAVE Params;
	WAVE HoldWave;
	WAVE XSA;
	Variable N = dimsize(Params, 0)
	Variable i = 0;
	DFREF MDFR = $ksMDFR;
	MAke/D/O/N=1 MDFR:XVec, MDFR:ParamInXVec
	MAke/O/D/N=(1,2) MDFR:XSAVec;
	WAVE XVec = MDFR:XVec;
	WAVE PIXVec  = MDFR:ParamInXVec
	WAVE XSAVec = MDFR:XSAVec;
	Variable XVec_i = 0

	Do
		if(!HoldWave[i])
			Redimension/N=(XVec_i+1) XVec, PIXVec
			Redimension/N= (XVec_i+1,2) XSAVec
			Xvec[XVec_i] = Params[i]
			XSAVec[XVec_i][] = XSA[i][q]
			PIXVec[XVec_i] = i
			XVec_i +=1;
		Endif
		i+=1;
	While(i<N)

	Return GetWavesDataFolder(XVec,2)+";"+GetWavesDataFolder(PIXVec,2)+";"+GetWavesDataFolder(XSAVec,2)+";"
end

Function ReadXW(XVec, PIXVec, Params)
	WAVE XVEc, PIXVec, Params; // read values out of the xvec and put them back into the parameter wave
	Variable N = dimsize(XVec, 0)
	Variable i = 0;

	Do
		Params[PIXVec[i]] = XVec[i]
		i+=1;
	While(i<N)

End



Function SolveODEAndFitImage(image) // Like SolveODEAndFitSlices(), but for the whole image. Call this when you have a good fit
// to a set of slices, and want to generate species associated spectra, and see if there are regions of the data that your model does not fit well.

	Wave Image
	DFREF MDFR=$ksMDFR
	Wave pw = MDFR:ParameterVals		// The rate constants and other wave-defining coefficients go here
	Wave xw= MDFR:Time_Base// This is the time base for the transients, but might not be used.
	DFREF MDFR =$ksMDFR
	Wave species = MDFR:Species
	Wave Transfer = MDFR:Transfer
```

```
	Wave Time_Base = MDFR:Time_Base
	Variable NEqn = itemsinlist(ksEqnNames)

	Species = 0;
	SolveODE(Time_base, "eqn", pw, species)
	//WAVE  = MDFR:SlicesToFit
	Variable ChaiSq = FitImage(Image, Species=Species)
End

Function FitODEToSlices() // Do the ODE model fit to the selected slices!
	DFREF MDFR = $ksMDFR
	WAVE eps = MDFR:Epsilon
	Wave Params = MDFR:ParameterVals
	WAVE HoldWave = MDFR:ParameterHolds
	WAVE XSA = MDFR:XSA
	Variable ApproxFuncValue = 0.01
	Variable FitMethod = 3 // sets the fit method. 3 = simulated annealing.
	String Vecs = SetXW(Params, HoldWave, XSA)
	WAVE XVec = $StringFromList(0,Vecs)
	WAVE PIXVec = $StringFromList(1,Vecs)
	WAVE XSAVec = $StringFromList(2,Vecs)
	Duplicate/O XVec MDFR:SimAnnBestXVec
	WAVE BXVec = MDFR:SimAnnBestXVec;
	if(FitMethod == 3)
		Optimize/I={100, 10, 5}/DSA = BXVec/D=4/XSA=XSAVec/T={10,0.0001}/M={3,0}/A=0/R=XVec/X=XVec/F=1e-2/Y=1e-9/T={8.53618e-7, 7.28664e-13} MeritSlices, PiXVec
		ReadXW(BXVec, PIXVec, Params)
		SolveODEAndFitSlices()
	Else
		Optimize/D=4/M={FitMethod,1}/A=0/R=XVec/X=XVec/F=1e-2/Y=(ApproxFuncValue)/T={8.53618e-7, 7.28664e-13} MeritSlices, PiXVec
		ReadXW(XVec, PIXVec, Params)
	Endif

End

Function/T StitchImagesByRow(Image1, Image2, stitchVal, StitchKey) // stitch two images together concatenate along one axis and interpolate along the other.
	WAVE Image1, Image2;
	Variable StitchVal; // x or y value at which to stitch the data. Say, 800 nm, for example, to stitch together NIR and VIS data.
	String stitchkey; // keyword identifying the x or y axis. In the present case this can be "WL" or "t"

	// 1. Find out if the stitch point is vaild for both images. Return an error if it isn't
	Variable StitchPoint1 = floor(CheckStitchPoint(Image1, StitchKey, StitchVal))
	Variable StitchPoint2 = Ceil(CheckStitchPoint(Image2, StitchKey, StitchVal))

	if(StitchPoint1 <0 || StitchPoint2 < 0)
		Print "error: either or both images do not contain the specified stitch location on the specified axis"
		Return ""
	Endif

	// 2. Figure out how many rows (or columns) are to be taken from each image

	Variable N1 = Stitchpoint1 + 1;
	Variable N2 = dimsize(Image2,0)-(Stitchpoint2+1);
	Variable N = N1+N2;
	Variable M = dimsize(Image1,1);

	// 3. Make a new image with all the required dimensions
```

```
	DFREF TargetFolder = GetWavesDAtaFolderDFR(Image1)
	String Ext = "_sti"
	String TargetName = nameofwave(Image1) + Ext
	Duplicate/O Image1, TargetFolder:$TargetName
	WAVE Target = TargetFolder:$TargetName
	Redimension/N=(N,M) Target; // by copying and redimensioning this wave it retains the attached notes with reference to the time and WL waves. The latter will need
	Target = 0;
	// 4. Copy Image1 into the new wave, stopping just before the stitch point

	CopyRowByRow(Image1, Target, 0, StitchPoint1, 0);

	// 5. Linearly interpolate rows or columns of image2 to match the x (or y) scaling of image1, and copy the interpolated columns into the new image

	InterpRowByRow(Image2, Target, StitchPoint2, dimsize(image2,0), Stitchpoint1)

	// 6. Make a new x or y wave as appropriate, and add these to the wavenotes of the new image so that it can be readily plotted.

	Wave WL1 = $GetNote(Image1, "WL")
	WAVE WL2 = $GetNote(Image2,"WL")

	WAVE WL3 = $SpliceAtPoint(StitchPoint1, Stitchpoint2, WL1, WL2, ext)

	SetNoteByKey(Target, GetWavesDataFolder(WL3,2), "WL")

End

Function CheckStitchPoint(Image, StitchKey, StitchPoint) //Take transiant spectrum image (image) and a keyword (stitchkey) identifying the axis to be checked.
																	// Find out if the stitchpoint exists along the in
	WAVE Image;
	STring StitchKey
	Variable StitchPoint
	WAVE Xwave = $GetNote(Image, StitchKey)
	FindLevel/Q/P XWave, StitchPoint
	if(V_flag)
		Return -1
	Endif

	Return V_LevelX

ENd

Function CopyRowByRow(SourceWave, TargetWave, start, stop, TargetSTart) //Copy one wave into another over a range of rows. TargetWave must be at least as large as SourceW
	WAVE SourceWave, TargetWAve; // data source and target
	//Variable Dim // dimension to cycle through and copy
	Variable Start, Stop; // starting and stopping indecies for source wave
	Variable TargetStart;// starting point in the target wave.
	Variable N= Stop-Start // number of things to copy.
	Variable i = 0;

	Do
		TargetWave[i+Targetstart][] = SourceWave[i+Start][q]
		i+=1;
	While(i<N)
End
```

```
Function InterpRowByRow(SourceWave, TargetWave, start, stop, TargetSTart) //Copy one wave into another over a range of rows. TargetWave must be at least as large as Sourc
	WAVE SourceWave, TargetWAve; // data source and target
	//Variable Dim // dimension to cycle through and copy
	Variable Start, Stop; // starting and stopping indecies for source wave
	Variable TargetStart;// starting point in the target wave.
	DFREF MDFR = $ksMDFR;
	Variable N= Stop-Start // number of things to copy.
	Variable i = 0;
	WAVE TargetTime = $GetNote(TargetWave,"t") // get time wave associated with the target.
	Wave SourceTime = $GetNote(SourceWave,"t") // get the time wave associated with the source.

	Do
		Duplicate/O/R=[i+Start][] SourceWave, MDFR:InterpTemp
		WAVE Temp = MDFR:InterpTemp
		matrixTranspose Temp
		Redimension/N=(Dimsize(Temp,0)) Temp;
		TargetWave[i+Targetstart][] = interp(TargetTime[q], SourceTime, Temp)
		i+=1;
	While(i<N)
	Killwaves/Z Temp;
End

Function/T SpliceAtPoint(StitchPoint1, Stitchpoint2, Wave1, Wave2, ext)
	Variable StitchPoint1, StitchPoint2;
	WAVE WAve1, Wave2;
	STring ext;
	Variable N = Stitchpoint1 + 1 + Dimsize(Wave2,0)-Stitchpoint2-1;
	DFREF TargetFolder = getwavesdataFolderDFR(Wave1)
	String TargetName = NameofWave(Wave1)+ ext
	MAke/O/N=(N) TargetFolder:$TargetName;
	WAVE Target = Targetfolder:$TargetName;
	Variable i = 0;

	Do
		if(i<=StitchPoint1)
			Target[i] = Wave1[i]
		Else
			Target[i] = Wave2[i-StitchPoint1-1+StitchPoint2]
		Endif

		i+=1;
	While(i<N)

	REturn GetWavesDataFolder(Target,2)

End

Function RemoveTimeSliceAB()
	WAVE Matrix = csrwaveref(A)
	Variable t1 = qcsr(A)
	Variable t2 = qcsr(B)
	Variable WL1 = pcsr(A)
	Variable WL2 = pcsr(B)
	Variable dWL = ABS(WL1-WL2)
	Variable dt = abs(t1-t2)
	Variable i = 0;
```

```
	Variable WL = WL1;
	Variable t = t1

	if(WL1>WL2)
		WL = WL2
	Endif

	if(t1>t2)
		t = t2
	Endif

	if(dWL >= dt)
		Do
			matrix[WL+i][] = 0
			i+=1;
		While(i<dWL)
	Else

		Do
			matrix[][t+i] = 0
			i+=1;
		While(i<dt)

	Endif
End

Function CalcFluence(FWHM, energy, WL)
	Variable FWHM // of laser spot in m
	variable energy // per pulse in J
	Variable WL // of pulse in nm
	Variable h = 6.626e-34 // plancks constant
	variable c = 2.998e8 // speed of light
	Variable E = h*c/(WL*1e-9) // photon, in J
	Variable N = energy/E // number of photons
	Variable F = N/(Pi*(FWHM/2*1e2)^2)

	Return F
End

Function FormatSASGraph()
	//ModifyGraph muloffset(species_0)={0,-1}
	ModifyGraph tick=2,mirror=1,fSize=12,axThick=2,notation=1;DelayUpdate
	Label left "OD";DelayUpdate
	Label bottom "wavelength (nm)"
	ModifyGraph width=200,height=200
	ModifyGraph fStyle=0,axThick=1
	Label left "OD x10\\S-4\\M";DelayUpdate
	ModifyGraph prescaleExp(left)=4
End

Function FormatSTFGraph()
	ModifyGraph axThick=2,notation=1;DelayUpdate
	Label left "OD";DelayUpdate
	Label bottom "time (s)"
	ModifyGraph width=200,height=200
	ModifyGraph fStyle=0,axThick=1
```

```
        Label left "OD x10\\S-4\\M";DelayUpdate
        ModifyGraph prescaleExp(left)=4
        ModifyGraph margin(right)=72
        Legend/C/N=text0/J/X=0.00/Y=0.00
End

Function FormatImageGraph()
        ModifyGraph tick=2,mirror=1,fSize=12,axThick=2;DelayUpdate
        Label bottom "wavelength (nm)"
        Label left "time (s)"
        ModifyGraph fStyle=1
        ModifyGraph width=200,height=200
        ModifyGraph fStyle=0,axThick=1
        SetAxis left 1e-14,5e-09
End
```
